# Supplementary material for: Feasibility and safety of targeted focal microwave ablation of the index tumor in patients with low to intermediate risk prostate cancer: Results of the FOSTINE trial
Source: PLoS One. 2021 Jul 14;16(7):e0252040. doi: 10.1371/journal.pone.0252040 (PMC8279354; doi:10.1371/journal.pone.0252040)
Supplement: S1 File — (DOCX) [file pone.0252040.s002.docx]

**RAPPORT D’ANALYSE STATISTIQUE**

| Étude évaluant la faisabilité et la tolérance d’un traitement focal transrectal par micro-ondes de la tumeur index des patients ayant un cancer de la prostate à risque faible ou risque intermédiaire bas de progression.  **FOSTINE** |
| --- |

**Investigateur coordonnateur :** Nicolas BARRY DELONGCHAMPS

**Protocole** : P160301 / 2016-A01065-46 (Version n° 4.0 du 09/07/2018)

**Le 15 juillet 2019**

Rédigé par Léa JILET (biostatisticienne)

URC/CIC Necker Cochin - Site Tarnier

89 rue d'Assas - 75006 Paris

01 58 41 33 85

PAGE DE SIGNATURE DU RAPPORT D’ANALYSE STATISTIQUE

Code de la Recherche : **P160301**

Protocole version n° 4.0 du : 09/07/2018

Plan d’analyse statistique version n° 1.0 du : 24/06/2019

|  | **DATE** | **SIGNATURE** |
| --- | --- | --- |
| **Investigateur Coordonnateur**  Nom : Nicolas BARRY DELONGCHAMPS |  |  |
| **Responsable biostatistique**  Nom : Hendy ABDOUL |  |  |
| **Biostatisticien de l’étude**  Nom : Léa JILET |  |  |
| **Référent projet de l’URC Necker-Cochin**  Nom : Guillaume MASSON |  |  |

Table des matières

[1. Introduction 4](#_Toc13843027)

[2. Objectifs de la recherche 4](#_Toc13843028)

[2.1. Objectif principal 4](#_Toc13843029)

[2.2. Objectifs secondaires 4](#_Toc13843030)

[3. Plan expérimental 4](#_Toc13843031)

[4. Critères d’évaluation 4](#_Toc13843032)

[4.1. Critère de jugement principal 4](#_Toc13843033)

[4.2. Critères de jugement secondaires 5](#_Toc13843034)

[5. Nombre de sujets nécessaire 5](#_Toc13843035)

[6. Participants à l’étude 6](#_Toc13843036)

[7. Analyse statistique 7](#_Toc13843037)

[7.1. Analyse descriptive 7](#_Toc13843038)

[7.1.1. Caractéristiques démographiques 7](#_Toc13843039)

[7.1.2. Examen clinique 7](#_Toc13843040)

[7.1.3. Biopsies 8](#_Toc13843041)

[7.1.4. Questionnaire 10](#_Toc13843042)

[7.1.5. Intervention 11](#_Toc13843043)

[7.2. Analyse principale 12](#_Toc13843044)

[7.3. Analyses secondaires 13](#_Toc13843045)

[7.3.1. Tolérance sexuelle 13](#_Toc13843046)

[7.3.2. Tolérance urinaire 16](#_Toc13843047)

[7.3.3. Évolution carcinologique 19](#_Toc13843048)

[7.4. Analyses complémentaires 21](#_Toc13843049)

# Introduction

Ce rapport d’analyse statistique s’appuie sur la version n° 4 du protocole P160301 / 2016-A01065-46 datant du 9 juillet 2018 et sur le plan d’analyse statistique version n° 1.0 signé le 24 juin 2019.

# Objectifs de la recherche

## Objectif principal

L’objectif principal est d’évaluer la faisabilité du traitement transrectal ablatif focal par micro-ondes d’une tumeur prostatique index identifiée par IRM multiparamétrique, chez les patients ayant un cancer de la prostate à faible risque de progression selon la classification de d’Amico.

## Objectifs secondaires

Les objectifs secondaires sont :

- d’évaluer le degré d’extension du traitement transrectal focal par micro-ondes de la tumeur index prostatique ;
- d’évaluer la tolérance sexuelle du traitement transrectal focal par micro-ondes de la tumeur index prostatique ;
- d’évaluer la tolérance urinaire du traitement transrectal focal par micro-ondes de la tumeur index prostatique ;
- d’évaluer l’évolution carcinologique à 6 mois du traitement transrectal focal par micro-ondes de la tumeur index prostatique.

# Plan expérimental

Il s’agit d’une étude interventionnelle monocentrique, ouverte, non comparative évaluant la faisabilité et la tolérance d’un traitement focal transrectal par micro-ondes de la tumeur index des patients ayant un cancer de la prostate à risque faible ou à risque intermédiaire bas de progression.

# Critères d’évaluation

## Critère de jugement principal

Le degré de nécrose de la tumeur index est évalué par une IRM multiparamétrique de la prostate réalisée à J7 (+2 jours si nécessaire) après le traitement, dans le service de Radiologie A de l’hôpital Cochin.

Le critère de jugement principal est la nécrose complète du volume cible défini sur l’IRM avant traitement. La nécrose complète est définie par l’absence de rehaussement à l’IRM dynamique de contraste dans la zone cible délimitée juste avant le traitement sur l’IRM multiparamétrique.

## Critères de jugement secondaires

Les critères de jugement secondaires sont :

- Le **degré d’extension du traitement** défini sur l’IRM à J7 après traitement par la longueur (en millimètres) des marges minimales et maximales de nécrose en dehors du volume cible, dans les trois plans de l’espace.

Les marges minimales et maximales de nécrose en dehors du volume cible délimité sur l’IRM préopératoire seront mesurées en comparant l’IRM multiparamétrique de la prostate réalisée 7 jours après le traitement avec l’IRM multiparamétrique de la prostate réalisée avant le traitement.

- La **tolérance sexuelle** évaluée par les auto-questionnaires IIEF (fonction érectile) et MSHQ-Ej (fonction éjaculatoire). Ces auto-questionnaires seront remplis par le patient au cours des visites de suivi à J7, 2 et 6 mois après l’intervention.
- La **tolérance urinaire** évaluée par les auto-questionnaires IPSS et IPSS-QDV, l’examen cytobactériologique des urines, le débit urinaire maximal, le Prostate Specific Antigen (PSA) total sérique, et l’introduction de tout traitement additionnel à visée urinaire.

Les auto-questionnaires seront remplis par le patient au cours des visites de suivi à J7, 2 et 6 mois après l’intervention. L’examen cytobactériologique des urines sera réalisé au cours des visites de suivi à J7, 2 et 6 mois après l’intervention. Le débit urinaire sera mesuré au cours des visites de suivi à J7, 2 et 6 mois après l’intervention et le taux de PSA sera mesuré à 2 et 6 mois après l’intervention.

L’**évolution carcinologique** sera évaluée par une IRM multiparamétrique de la prostate et des biopsies ciblées au niveau du volume traité, 6 mois après l’intervention. Les biopsies de contrôle de la prostate seront réalisées 6 mois après l’intervention au sein du service de radiologie de l’hôpital Cochin et seront analysées en anatomopathologie en aveugle du résultat IRM.

# Nombre de sujets nécessaire

Le nombre de sujets nécessaire est défini dans le protocole comme suit :

« Un design d’étude de phase II a été utilisé pour le calcul du nombre de sujet en considérant qu’un taux de nécrose complète de 99 % serait désirable. Si ce taux était de 60 % ou moins l’intervention serait considérée comme inefficace.

Conformément à ces hypothèses et avec un risque alpha de 0.1 et un risque beta de 0.1, un total de 5 patients devrait être nécessaire. Pour pouvoir étudier la tolérance de l’intervention un nombre supplémentaire de 6 patients sera prévu soit un total de 11 patients à inclure pour avoir in fine 10 patients analysables. »

# Participants à l’étude

Pour rappel, la population d’analyse a été définie comme suit :

- **Intention-de-traiter modifiée (ITTm)** : tous les patients inclus ayant reçu l’intervention et pour lesquels le critère de jugement principal est disponible à J7.

**Figure 1 : Flowchart de l’étude**


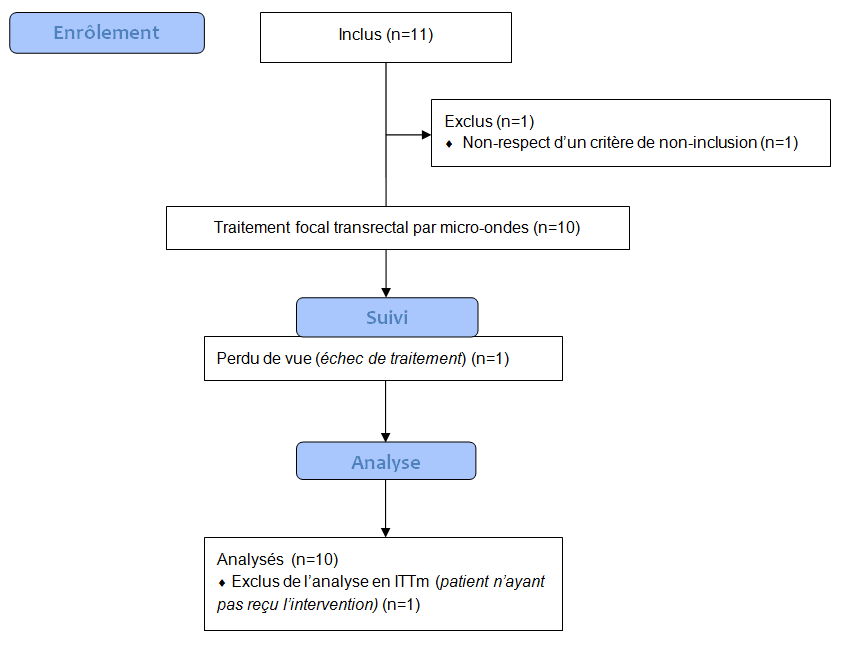


# Analyse statistique

Les données ont été analysés à l’aide du logiciel SAS Version 9.4 (Copyright© 2016 by SAS Institute Inc., Cary, NC, USA.).

Tous les tests réalisés sont bilatéraux avec un risque α de 5 % et un seuil de significativité de 5 %.

## Analyse descriptive

### Caractéristiques démographiques

**Tableau 2 : Analyse descriptive des données sociodémographiques**

|  | **Total n=10** |
| --- | --- |
| **Âge (années)** |  |
| Manquant | 0 |
| Moyenne (écart-type) | 65.7 (5.8) |
| Médiane (Q1-Q3) | 64.5 (61.0-72.0) |
| Min - Max | 58.0-74.0 |
|  |  |

### Examen clinique

**Tableau 3 : Analyse descriptive des examens cliniques**

|  | **Total n=10** |
| --- | --- |
| **Délai depuis le diagnostic de cancer (mois)** |  |
| Manquant | 0 |
| Moyenne (écart-type) | 2.0 (1.4) |
| Médiane (Q1-Q3) | 1.5 (1.0-3.0) |
| Min - Max | 0.0-4.0 |
|  |  |
| **Stade du cancer – n (%)** |  |
| T1c | 9 (90.0) |
| T2a | 1 (10.0) |
|  |  |
| **Localisation de la tumeur index – n (%)** |  |
| Droite | 3 (30.0) |
| Gauche | 7 (70.0) |
|  |  |
| ZP | 6 (60.0) |
| ZT | 4 (40.0) |
|  |  |
| Apex | 3 (30.0) |
| Zone moyenne | 3 (30.0) |
| Base | 4 (40.0) |
|  |  |
| **Grand axe (mm)** |  |
| Manquant | 0 |
| Moyenne (écart-type) | 12.1 (4.8) |
| Médiane (Q1-Q3) | 11.5 (9.0-15.0) |
| Min - Max | 5.0-20.0 |
|  |  |
| **Toucher rectal** **– n (%)** |  |
| Normal | 10 (100.0) |
|  |  |
| **PSA (ng/ml)** |  |
| Manquant | 0 |
| Moyenne (écart-type) | 5.9 (2.3) |
| Médiane (Q1-Q3) | 5.0 (4.3-8.1) |
| Min - Max | 3.9-10.6 |
|  |  |
| **Gleason de la tumeur index – n (%)** |  |
| 3+3 | 8 (80.0) |
| 3+4 | 2 (20.0) |
|  |  |
| **Gleason des tumeurs secondaires** (seulement 2 patients avec des tumeurs secondaires) **– n (%)** |  |
| 3+3 | 2 (20.0) |
|  |  |

### Biopsies

**Tableau 4 : Analyse descriptive des biopsies**

|  | **Total n=10** |
| --- | --- |
| **Nombre de carottes systématisées** |  |
| Manquant | 0 |
| Moyenne (écart-type) | 9.4 (4.6) |
| Médiane (Q1-Q3) | 11.5 (6.0-12.0) |
| Min - Max | 0.0-15.0 |
|  |  |
| **Nombre de carottes ciblées** |  |
| Manquant | 0 |
| Moyenne (écart-type) | 3.2 (1.2) |
| Médiane (Q1-Q3) | 3.0 (3.0-4.0) |
| Min - Max | 1.0-5.0 |
|  |  |
| **Biopsies avec cancer – n (%)** |  |
| Oui | 10 (100.0) |
| **Si oui, combien ?** |  |
| Moyenne (écart-type) | 3.0 (1.2) |
| Médiane (Q1-Q3) | 3.0 (3.0-4.0) |
| Min - Max | 1.0-5.0 |

**Tableau 5 : Détail des biopsies**

| **Patient** | **Type de biopsie** | **Longueur du cancer** | **Score de Gleason** | **Pourcentage de grade 4** |
| --- | --- | --- | --- | --- |
| 001-0001-M-P | Ciblée | 3 | 3+3 | 0 |
| 001-0002-L-B | Ciblée | 5 | 3+4 | 30 |
| 001-0002-L-B | Ciblée | 7 | 3+4 | 30 |
| 001-0002-L-B | Ciblée | 7 | 3+4 | 30 |
| 001-0004-A-J | Ciblée | 7 | 3+3 | 0 |
| 001-0004-A-J | Ciblée | 7 | 3+3 | 0 |
| 001-0004-A-J | Ciblée | 7 | 3+3 | 0 |
| 001-0005-B-S | Ciblée | 6 | 3+4 | 30 |
| 001-0005-B-S | Ciblée | 6 | 3+4 | 30 |
| 001-0005-B-S | Ciblée | 6 | 3+4 | 30 |
| 001-0005-B-S | Systématisée | 4 | 3+3 | 0 |
| 001-0005-B-S | Systématisée | 5 | 3+3 | 0 |
| 001-0006-N-J | Systématisée | 5 | 3+3 | 0 |
| 001-0006-N-J | Ciblée | 9 | 3+3 | 0 |
| 001-0006-N-J | Ciblée | 9 | 3+3 | 0 |
| 001-0006-N-J | Ciblée | 8 | 3+3 | 0 |
| 001-0007-G-J | Ciblée | 5 | 3+3 | 0 |
| 001-0007-G-J | Ciblée | 5 | 3+3 | 0 |
| 001-0007-G-J | Ciblée | 5 | 3+3 | 0 |
| 001-0008-R-E | Ciblée | 3 | 3+3 | 0 |
| 001-0008-R-E | Ciblée | 7 | 3+3 | 0 |
| 001-0008-R-E | Systématisée | 3 | 3+3 | 0 |
| 001-0008-R-E | Systématisée | 1 | 3+3 | 0 |
| 001-0010-S-J | Ciblée | 7 | 3+3 | 0 |
| 001-0011-D-J | Ciblée | 11 | 3+3 | 0 |
| 001-0011-D-J | Ciblée | 4 | 3+3 | 0 |
| 001-0011-D-J | Ciblée | 11 | 3+3 | 0 |

### Questionnaire

**Tableau 6 : Analyse descriptive des questionnaires**

|  | **Total n=10** |
| --- | --- |
| **IPSS à V0** |  |
| Manquant | 0 |
| Moyenne (écart-type) | 8.9 (5.0) |
| Médiane (Q1-Q3) | 8.5 (4.0-13.0) |
| Min - Max | 4.0-16.0 |
|  |  |
| **IPSS - Atteinte urinaire – n (%)** |  |
| Légère | 5 (50.0) |
| Modérée | 5 (50.0) |
| Sévère | 0 (0) |
|  |  |
| **IPSS-QDV à V0** |  |
| Manquant | 0 |
| Moyenne (écart-type) | 1.7 (1.1) |
| Médiane (Q1-Q3) | 1.0 (1.0-2.0) |
| Min - Max | 1.0-4.0 |
|  |  |
| **IIEF5 à V0** |  |
| Manquant | 0 |
| Moyenne (écart-type) | 16.2 (8.0) |
| Médiane (Q1-Q3) | 18.5 (13.0-23.0) |
| Min - Max | 3.0-25.0 |
|  |  |
| **IIEF5 – Atteinte sexuelle – n (%)** |  |
| Trouble de l’érection sévère | 0 (0) |
| Trouble de l’érection modéré | 2 (20.0) |
| Trouble de l’érection léger | 1 (10.0) |
| Fonction érectile normale | 5 (50.0) |
| Non interprétable | 2 (20.0) |
|  |  |
| **MSHQ-EJD (symptômes) à V0** |  |
| Manquant | 0 |
| Moyenne (écart-type) | 9.8 (4.0) |
| Médiane (Q1-Q3) | 10.5 (7.0-13.0) |
| Min - Max | 1.0-15.0 |
|  |  |
| **MSHQ-EJD (gêne) à V0** |  |
| Manquant | 0 |
| Moyenne (écart-type) | 0.8 (1.3) |
| Médiane (Q1-Q3) | 0.0 (0.0-1.0) |
| Min - Max | 0.0-4.0 |

### Intervention

**Tableau 7 : Analyse descriptive de l’intervention**

|  | **Total n=10** |
| --- | --- |
| **Durée du traitement (min)** |  |
| Manquant | 0 |
| Moyenne (écart-type) | 19.4 (9.8) |
| Médiane (Q1-Q3) | 15.5 (12.0-24.0) |
| Min - Max | 11.0-38.0 |
|  |  |
| **Puissance délivrée (watts)** |  |
| Manquant | 0 |
| Moyenne (écart-type) | 14.0 (2.1) |
| Médiane (Q1-Q3) | 15.0 (15.0-15.0) |
| Min - Max | 10.0-15.0 |
|  |  |
| **EI pendant l’intervention – n (%)** |  |
| Non | 9 (90.0) |
| Oui | 1 (10.0) * |
|  |  |
| **Traitement de soutien– n (%)** |  |
| Non | 9 (90.0) |
| Oui | 1 (10.0) * |
|  |  |
| **EI post-intervention– n (%)** |  |
| Non | 10 (100.0) |
| Oui | 0 (0) |
|  |  |

** Le patient 001-0001-M-P a pris des antidouleurs en raison de douleurs au cours de l'intervention.*

## Analyse principale

Pour rappel, le critère de jugement principal est la nécrose complète du volume cible à 7 jour post-intervention.

**Tableau 8 : Nécrose complète**

|  | **Total n=10** |
| --- | --- |
| **Nécrose totale du volume cible – n (% [IC95%])** |  |
| Non | 2 (20.0 [0.0-44.8]) |
| Oui | 8 (80.0 [55.2-100.0]) |

Il y a 8 succès à 7 jours post-intervention, soit une **efficacité de 80 % (IC95 % = [55 %-100 %])**.

## Analyses secondaires

### Tolérance sexuelle

**Tableau 9 : Données individuelles de tolérance sexuelle**

| **Patient** | **Date de la visite V2** | **IIEF5 à V2** | **MSHQ-EJD (symptômes) à V2** | **MSHQ-EJD (gêne) à V2** | **Date de la visite V3** | **IIEF5 à V3** | **MSHQ-EJD (symptômes) à V3** | **MSHQ-EJD (gêne) à V3** | **Date de la visite V4** | **IIEF5 à V4** | **MSHQ-EJD (symptômes) à V4** | **MSHQ-EJD (gêne) à V4** |
| --- | --- | --- | --- | --- | --- | --- | --- | --- | --- | --- | --- | --- |
| 001-0001-M-P | 28/09/2017 | 4 | 8 | 0 | 27/11/2017 | . | . | . | 14/06/2018 | 16 | 7 | 1 |
| 001-0002-L-B | 06/12/2017 | 2 | 15 | 0 | 29/01/2018 | 1 | 1 | 0 | 28/05/2018 | 1 | . | . |
| 001-0004-A-J | 11/12/2017 | 1 | . | . | 29/01/2018 | 2 | 13 | 0 | 30/05/2018 | 7 | 9 | 2 |
| 001-0005-B-S | 11/06/2018 | 22 | 15 | 0 | 30/07/2018 | 25 | 15 | 0 | 17/12/2018 | . | . | . |
| 001-0006-N-J | 07/06/2018 | 14 | 15 | 0 | . | . | . | . | . | . | . | . |
| 001-0007-G-J | 20/06/2018 | 14 | 10 | 0 | 02/08/2018 | 13 | 13 | 2 | 18/12/2018 | 16 | 11 | 3 |
| 001-0008-R-E | 11/07/2018 | 25 | 13 | 0 | 10/09/2018 | 25 | 11 | 1 | 28/01/2019 | 25 | 13 | 0 |
| 001-0009-P-D | 11/07/2018 | 8 | 8 | 4 | 10/09/2018 | 2 | . | . | 04/02/2019 | 2 | . | . |
| 001-0010-S-J | 17/09/2018 | 3 | . | . | 05/11/2018 | 14 | 14 | 0 | 11/03/2019 | 14 | 12 | 1 |
| 001-0011-D-J | 05/11/2018 | . | 14 | 1 | 17/12/2018 | 24 | 15 | 0 | 23/04/2019 | 20 | 12 | 1 |

**Tableau 10 : Analyse de la tolérance sexuelle au cours du suivi**

|  | **Total n=10** |
| --- | --- |
| **IIEF5 à V2** |  |
| Manquant | 1 |
| Moyenne (écart-type) | 10.3 (8.9) |
| Médiane (Q1-Q3) | 8.0 (3.0-14.0) |
| Min - Max | 1.0-25.0 |
|  |  |
| **IIEF5 à V2 – Atteinte sexuelle – n (%)** |  |
| Manquant | 1 (10.0) |
| Trouble de l’érection sévère | 1 (10.0) |
| Trouble de l’érection modéré | 2 (20.0) |
| Trouble de l’érection léger | 0 (0) |
| Fonction érectile normale | 2 (20.0) |
| Non interprétable | 0 (0) |
|  |  |
| **MSHQ-EJD (symptômes) à V2** |  |
| Manquant | 2 |
| Moyenne (écart-type) | 12.3 (3.1) |
| Médiane (Q1-Q3) | 13.5 (9.0-15.0) |
| Min - Max | 8.0-15.0 |
|  |  |
| **MSHQ-EJD (gêne) à V2** |  |
| Manquant | 2 |
| Moyenne (écart-type) | 0.6 (1.4) |
| Médiane (Q1-Q3) | 0.0 (0.0-0.5) |
| Min - Max | 0.0-4.0 |
|  |  |
| **IIEF5 à V3** |  |
| Manquant | 2 |
| Moyenne (écart-type) | 13.3 (10.7) |
| Médiane (Q1-Q3) | 13.5 (2.0-24.5) |
| Min - Max | 1.0-25.0 |
|  |  |
| **IIEF5 à V3 – Atteinte sexuelle – n (%)** |  |
| Manquant | 2 (20.0) |
| Trouble de l’érection sévère | 0 (0) |
| Trouble de l’érection modéré | 2 (20.0) |
| Trouble de l’érection léger | 0 (0) |
| Fonction érectile normale | 3 (30.0) |
| Non interprétable | 3 (30.0) |
|  |  |
|  |  |
|  |  |
|  |  |
| **MSHQ-EJD (symptômes) à V3** |  |
| Manquant | 3 |
| Moyenne (écart-type) | 11.7 (4.9) |
| Médiane (Q1-Q3) | 13.0 (11.0-15.0) |
| Min - Max | 1.0-15.0 |
|  |  |
| **MSHQ-EJD (gêne) à V3** |  |
| Manquant | 3 |
| Moyenne (écart-type) | 0.4 (0.8) |
| Médiane (Q1-Q3) | 0.0 (0.0-1.0) |
| Min - Max | 0.0-2.0 |
|  |  |
| **IIEF5 à V4** |  |
| Manquant | 2 |
| Moyenne (écart-type) | 12.6 (8.6) |
| Médiane (Q1-Q3) | 15.0 (4.5-18.0) |
| Min - Max | 1.0-25.0 |
|  |  |
| **IIEF5 à V4 – Atteinte sexuelle – n (%)** |  |
| Manquant | 2 (20.0) |
| Trouble de l’érection sévère | 1 (10.0) |
| Trouble de l’érection modéré | 1 (10.0) |
| Trouble de l’érection léger | 3 (30.0) |
| Fonction érectile normale | 1 (10.0) |
| Non interprétable | 2 (20.0) |
|  |  |
| **MSHQ-EJD (symptômes) à V4** |  |
| Manquant | 4 |
| Moyenne (écart-type) | 10.7 (2.3) |
| Médiane (Q1-Q3) | 11.5 (9.0-12.0) |
| Min - Max | 7.0-13.0 |
|  |  |
| **MSHQ-EJD (gêne) à V4** |  |
| Manquant | 4 |
| Moyenne (écart-type) | 1.3 (1.0) |
| Médiane (Q1-Q3) | 1.0 (1.0-2.0) |
| Min - Max | 0.0-3.0 |
|  |  |

### Tolérance urinaire

**Tableau 11 : Données individuelles de tolérance urinaire**

| **Patient** | **Date de la visite V2** | **IPSS à V2** | **Qualité de vie à V2** | **Stérilité des urines à V2** | **Traitement additionnel de l'HBP** | **Date de la visite V3** | **PSA à V3 (ng/ml)** | **IPSS à V3** | **Qualité de vie à V3** | **Stérilité des urines à V3** | **Traitement additionnel de l'HBP** | **Date de la visite V4** | **PSA à V4 (ng/ml)** | **IPSS à V4** | **Qualité de vie à V4** | **Stérilité des urines à V4** | **Traitement additionnel de l'HBP** |
| --- | --- | --- | --- | --- | --- | --- | --- | --- | --- | --- | --- | --- | --- | --- | --- | --- | --- |
| 001-0001-M-P | 28/09/2017 | 11 | 2 | Oui | Non | 27/11/2017 | 4.9 | 1 | 0 | Oui | Non | 14/06/2018 | 4.9 | 13 | 2 | . | Non |
| 001-0002-L-B | 06/12/2017 | 12 | 2 | Oui | Non | 29/01/2018 | 2.2 | 6 | 0 | Oui | Non | 28/05/2018 | 1.2 | 8 | 2 | Oui | Non |
| 001-0004-A-J | 11/12/2017 | 2 | 2 | Oui | Non | 29/01/2018 | 12.8 | 6 | 1 | Oui | Non | 30/05/2018 | 9.9 | 10 | 3 | Oui | Non |
| 001-0005-B-S | 11/06/2018 | 2 | 1 | . | Non | 30/07/2018 | 7.8 | 2 | 0 | Oui | Non | 17/12/2018 | 9.4 | . | . | . | Non |
| 001-0006-N-J | 07/06/2018 | 2 | 1 | Oui | Non | . | . | . | . | . | . | . | . | . | . | . | . |
| 001-0007-G-J | 20/06/2018 | 10 | 2 | Oui | Non | 02/08/2018 | 4.8 | 7 | 1 | Oui | Non | 18/12/2018 | 7.8 | 21 | 3 | . | Non |
| 001-0008-R-E | 11/07/2018 | 4 | 1 | Oui | Non | 10/09/2018 | 5.4 | 3 | 2 | Oui | Non | 28/01/2019 | 7.1 | 4 | 0 | . | Non |
| 001-0009-P-D | 11/07/2018 | 16 | 3 | Oui | Non | 10/09/2018 | 8.3 | 12 | 4 | Oui | Non | 04/02/2019 | 10.1 | 17 | 4 | . | Non |
| 001-0010-S-J | 17/09/2018 | 3 | 0 | Oui | Non | 05/11/2018 | 3.3 | 7 | 0 | Oui | Non | 11/03/2019 | 2.9 | 9 | 1 | Oui | Non |
| 001-0011-D-J | 05/11/2018 | 10 | 0 | Oui | Non | 17/12/2018 | 3.7 | 5 | 0 | . | Non | 23/04/2019 | . | 10 | 0 | . | Non |

**Tableau 12: Analyse de la tolérance urinaire au cours du suivi**

|  | **Total n=10** |
| --- | --- |
| **IPSS à V2** |  |
| Manquant | 0 |
| Moyenne (écart-type) | 7.2 (5.2) |
| Médiane (Q1-Q3) | 7.0 (2.0-11.0) |
| Min - Max | 2.0-16.0 |
|  |  |
| **IPSS à V2 - Atteinte urinaire – n (%)** |  |
| Légère | 5 (50.0) |
| Modérée | 5 (50.0) |
| Sévère | 0 (0) |
|  |  |
| **IPSS-QDV à V2** |  |
| Manquant | 0 |
| Moyenne (écart-type) | 1.4 (1.0) |
| Médiane (Q1-Q3) | 1.5 (1.0-2.0) |
| Min - Max | 0.0-3.0 |
|  |  |
| **PSA à V3 (ng/ml)** |  |
| Manquant | 1 |
| Moyenne (écart-type) | 5.9 (3.3) |
| Médiane (Q1-Q3) | 4.9 (3.7-7.8) |
| Min - Max | 2.2-12.8 |
|  |  |
| **IPSS à V3** |  |
| Manquant | 1 |
| Moyenne (écart-type) | 5.4 (3.3) |
| Médiane (Q1-Q3) | 6.0 (3.0-7.0) |
| Min - Max | 1.0-12.0 |
|  |  |
| **IPSS à V3 - Atteinte urinaire – n (%)** |  |
| Manquant | 1 (10.0) |
| Légère | 8 (80.0) |
| Modérée | 1 (10.0) |
| Sévère | 0 (0) |
|  |  |
| **IPSS-QDV à V3** |  |
| Manquant | 1 |
| Moyenne (écart-type) | 0.9 (1.4) |
| Médiane (Q1-Q3) | 0.0 (0.0-1.0) |
| Min - Max | 0.0-4.0) |
|  |  |
|  |  |
|  |  |
| **PSA à V4 (ng/ml)** |  |
| Manquant | 2 |
| Moyenne (écart-type) | 6.7 (3.3) |
| Médiane (Q1-Q3) | 7.5 (3.9-9.7) |
| Min - Max | 1.2-10.1 |
|  |  |
| **IPSS à V4** |  |
| Manquant | 2 |
| Moyenne (écart-type) | 11.5 (5.4) |
| Médiane (Q1-Q3) | 10.0 (8.5-15.0) |
| Min - Max | 4.0-21.0 |
|  |  |
| **IPSS à V4 - Atteinte urinaire – n (%)** |  |
| Manquant | 2 (20.0) |
| Légère | 1 (10.0) |
| Modérée | 6 (60.0) |
| Sévère | 1 (10.0) |
|  |  |
| **IPSS-QDV à V4** |  |
| Manquant | 2 |
| Moyenne (écart-type) | 1.9 (1.5) |
| Médiane (Q1-Q3) | 2.0 (0.5-3.0) |
| Min - Max | 0.0-4.0 |
|  |  |

### Évolution carcinologique

**Tableau 13 : Biopsies à V4**

| **Patient** | **Date des dernières biopsies à V4** | **Nb de carottes systématisées** | **Nb de carottes ciblées** | **Biopsies avec cancer à V4** | **Si oui, combien** |
| --- | --- | --- | --- | --- | --- |
| 001-0001-M-P | 26/03/2018 | 12 | 3 | Oui | 4 |
| 001-0002-L-B | 24/05/2018 | 3 | 6 | Non | . |
| 001-0004-A-J | 24/05/2018 | 6 | 4 | Oui | 2 |
| 001-0005-B-S | 26/11/2018 | 11 | 3 | Oui | 4 |
| 001-0006-N-J | . | . | . | . | . |
| 001-0007-G-J | 03/12/2018 | 12 | 7 | Oui | 6 |
| 001-0008-R-E | 07/01/2019 | 11 | 3 | Oui | 4 |
| 001-0009-P-D | 28/01/2019 | 12 | 3 | Oui | 3 |
| 001-0010-S-J | 25/02/2019 | 12 | 3 | Oui | 4 |
| 001-0011-D-J | 15/04/2019 | 5 | 5 | Non | . |

**Tableau 14 : Détails des résultats de biopsies**

| **Patient** | **Type de biopsie** | **Longueur du cancer** | **Score de Gleason** | **Pourcentage de grade 4** |
| --- | --- | --- | --- | --- |
| 001-0001-M-P | Systématisée | 2 | 3+3 | 0 |
| 001-0001-M-P | Ciblée | 3 | 3+3 | 0 |
| 001-0001-M-P | Ciblée | 4 | 3+3 | 0 |
| 001-0001-M-P | Ciblée | 4 | 3+3 | 0 |
| 001-0004-A-J | Ciblée | 8 | 3+3 | 0 |
| 001-0004-A-J | Ciblée | 10 | 3+3 | 0 |
| 001-0005-B-S | Systématisée | 6 | 3+4 | 10 |
| 001-0005-B-S | Systématisée | 6 | 3+4 | 10 |
| 001-0005-B-S | Ciblée | 3 | 3+4 | 10 |
| 001-0005-B-S | Ciblée | 3 | 3+4 | 10 |
| 001-0007-G-J | Ciblée | 5 | 3+3 | 0 |
| 001-0007-G-J | Systématisée | 6 | 3+4 | 10 |
| 001-0007-G-J | Systématisée | 6 | 3+4 | 10 |
| 001-0007-G-J | Systématisée | 1 | 3+3 | 0 |
| 001-0007-G-J | Systématisée | 1 | 3+3 | 0 |
| 001-0007-G-J | Systématisée | 4 | 3+3 | 0 |
| 001-0008-R-E | Systématisée | 5 | 3+4 | 15 |
| 001-0008-R-E | Systématisée | 5 | 3+4 | 15 |
| 001-0008-R-E | Systématisée | 5 | 3+4 | 15 |
| 001-0008-R-E | Systématisée | 5 | 3+4 | 15 |
| 001-0009-P-D | Ciblée | 3 | 3+3 | 0 |
| 001-0009-P-D | Ciblée | 3 | 3+3 | 0 |
| 001-0009-P-D | Ciblée | 4 | 3+3 | 0 |
| 001-0010-S-J | Ciblée | 3 | 3+3 | 0 |
| 001-0010-S-J | Ciblée | 3 | 3+3 | 0 |
| 001-0010-S-J | Ciblée | 2 | 3+3 | 0 |
| 001-0010-S-J | Systématisée | 0.5 | 3+3 | 0 |

## Analyses complémentaires

**Tableau 15 : Données individuelles de diamètre de la nécrose**

| **Patient** | **Diamètre maximal de la nécrose à V2 (mm)** |
| --- | --- |
| 001-0001-M-P | 17 |
| 001-0002-L-B | 16 |
| 001-0004-A-J | 22 |
| 001-0005-B-S | 12 |
| 001-0006-N-J | 26 |
| 001-0007-G-J | 17 |
| 001-0008-R-E | 18 |
| 001-0009-P-D | 13 |
| 001-0010-S-J | 26 |
| 001-0011-D-J | 20 |

**Tableau 16 : Données individuelles de superposition entre la zone cible et la tumeur index**

| **Patient** | **Superposition zone cible-tumeur index (%)** |
| --- | --- |
| 001-0001-M-P | 100 |
| 001-0002-L-B | 100 |
| 001-0004-A-J | 100 |
| 001-0005-B-S | 100 |
| 001-0006-N-J | 40 |
| 001-0007-G-J | 100 |
| 001-0008-R-E | 100 |
| 001-0009-P-D | 25 |
| 001-0010-S-J | 100 |
| 001-0011-D-J | 100 |

**Tableau 17 : Analyse des informations complémentaires sur la tumeur**

|  | **Total (n=10)** |
| --- | --- |
| **Diamètre maximal de la nécrose à V2 (mm)** |  |
| Manquant | 0 |
| Moyenne (écart-type) | 18.7 (4.8) |
| Médiane (Q1-Q3) | 17.5 (16.0-22.0) |
| Min - Max | 12.0-26.0 |
|  |  |
| **Superposition zone cible-tumeur index (%)** |  |
| Manquant | 0 |
| Moyenne (écart-type) | 86.5 (28.7) |
| Médiane (Q1-Q3) | 100.0 (100.0-100.0) |
| Min - Max | 25.0-100.0 |
