# Supplementary material for: Feasibility and safety of targeted focal microwave ablation of the index tumor in patients with low to intermediate risk prostate cancer: Results of the FOSTINE trial
Source: PLoS One. 2021 Jul 14;16(7):e0252040. doi: 10.1371/journal.pone.0252040 (PMC8279354; doi:10.1371/journal.pone.0252040)
Supplement: S2 File — (PDF) [file pone.0252040.s003.pdf]

ASSISTANCE PUBLIQUE HÔPITAUX DE PARIS

Paris le 01er avril 2021

Direction de la Recherche Clinique et de l'Innovation (DRCI)

Carré Historique de l'Hôpital Saint-Louis
Secteur Gris - Porte 23
1 avenue Claude Vellefaux
75475 PARIS
http://recherche-innovation.aphp.fr/

Directrice
Stéphanie DECOOPMAN

I, Stéphanie Decoopman, the undersigned Director of the Clinical Research and Innovation of AP-HP, and sponsor representative of the FOSTINE trial, agrees that the research protocol should be published under a CC-BY license.

Stéphanie Decoopman
Directrice de la DRCI

Erik DOMAIN
DRCI - AP-HP
Directeur adjoint
