# Supplementary material for: Feasibility and safety of targeted focal microwave ablation of the index tumor in patients with low to intermediate risk prostate cancer: Results of the FOSTINE trial
Source: PLoS One. 2021 Jul 14;16(7):e0252040. doi: 10.1371/journal.pone.0252040 (PMC8279354; doi:10.1371/journal.pone.0252040)
Supplement: S3 File — (PDF) [file pone.0252040.s004.pdf]

« Etude évaluant la faisabilité et la tolérance d'un traitement focal transrectal par micro-ondes de la tumeur index des patients ayant un cancer de la prostate à risque faible ou risque intermédiaire bas de progression » FOSTINE

PROTOCOLE DE RECHERCHE INTERVENTIONNELLE IMPLIQUANT LA PERSONNE HUMAINE PORTANT SUR UN DISPOSITIF MEDICAL OU SUR UN DISPOSITIF MEDICAL DE DIAGNOSTIC IN VITRO

Version N°3.0 du 07/03/2018
Code projet : P160301 / N°IDRCB : 2016-A01065-46

Investigateur coordonnateur : Nicolas BARRY DELONGCHAMPS
Service d'urologie
Hôpital Cochin
Tél 01 58 41 27 64
Courriel : nicolas.barry-delongchamps@aphp.fr

Responsable scientifique : Alexandre SCHULL
Service d'urologie
Hôpital Cochin
Tél : 01 58 41 27 48
Courriel : alexandre.schull@aphp.fr

Promoteur : AP-HP
et par délégation : Délégation à la Recherche Clinique et à l'Innovation (DRCI)
Hôpital Saint-Louis
1, avenue Claude Vellefaux
Référent projet DRCI-Siege: Karine GOUDE-ORY
Courriel : karine.goude@aphp.fr

Structure chargée du suivi de la recherche :
Unité de Recherche Clinique (URC) Necker Cochin
Pavillon Tarnier, 89 rue d'Assas 75006 Paris
Référent projet DRCI-URC : Clotilde SIMON
Courriel : clotilde.simon@aphp.fr

Délégation à la Recherche Clinique et à l'Innovation (DRCI)
Hôpital Saint Louis 75010 PARIS

Page de signature d'un protocole de recherche interventionnelle impliquant la personne humaine portant sur un dispositif médical ou sur un dispositif médical de diagnostic in vitro

Code de la Recherche : P160301
Titre : Etude évaluant la faisabilité et la tolérance d'un traitement focal transrectal par micro-ondes de la tumeur index des patients ayant un cancer de la prostate à risque faible ou risque intermédiaire bas de progression - FOSTINE
Version N°3.0 du 07/03/2018

La recherche sera conduite conformément au protocole, aux bonnes pratiques en vigueur et aux dispositions législatives et réglementaires en vigueur.

**L'investigateur coordonnateur** :	
Dr Nicolas BARRY DELONGCHAMPS	Date : ……………/………/………..
Service d'urologie	Signature :
Hôpital Cochin	
Paris	
**Le responsable scientifique** :	
Dr Alexandre SCHULL	Date : ……………/………/………..
Service d'urologie	Signature :
Hôpital Cochin	
Paris	
**Le promoteur**	
Assistance Publique – Hôpitaux de Paris	Date : ……………/………/………..
Délégation à la Recherche Clinique et à l'Innovation (DRCI)	Signature :
Hôpital Saint Louis	
1 avenue Claude Vellefaux	
75010 PARIS	

La recherche a reçu un avis favorable du CPP Ile de France III en date du 04/04/2017 et une autorisation de l'ANSM en date du 05/05/2017.

TABLE DES MATIÈRES

1 RÉSUMÉ SYNOPTIQUE 6

2 JUSTIFICATION SCIENTIFIQUE DE LA RECHERCHE 9

2.1 HYPOTHESES DE LA RECHERCHE 9
2.2 DESCRIPTION DES CONNAISSANCES RELATIVES A LA PATHOLOGIE CONCERNEE 9
2.3 RESUME DES EXPERIMENTATIONS PRE CLINIQUES ET DES ESSAIS CLINIQUES CONCERNES 13
2.4 DESCRIPTION DE LA POPULATION A ETUDIER ET JUSTIFICATION DE SON CHOIX 14
2.5 DENOMINATION ET DESCRIPTION DU DIPOSITIF MEDICAL 14
2.6 DESCRIPTION ET JUSTIFICATION DES MODALITES D'UTILISATION DU DISPOSITIF MEDICAL 15
2.7 RESUME DES BENEFICES ET DES RISQUES PREVISIBLES ET CONNUS POUR LES PERSONNES SE PRETANT A LA RECHERCHE 15
2.7.1 BENEFICES PREVISIBLES 15
2.7.2 RISQUES PREVISIBLES 15

3 OBJECTIFS 17

3.1 OBJECTIF PRINCIPAL 17
3.2 OBJECTIFS SECONDAIRES 17

4 CONCEPTION DE LA RECHERCHE 17

4.1 ENONCE PRECIS DES CRITERES D'EVALUATION PRINCIPAL ET SECONDAIRES 17
4.2 DESCRIPTION DE LA METHODOLOGIE DE LA RECHERCHE 18

5 DEROULEMENT DE LA RECHERCHE 18

5.1 VISITE DE SELECTION : V-1 18
5.2 VISITE D'INCLUSION : V0 19
5.3 VISITES DE SUIVI DE LA RECHERCHE 20
5.4 VISITE DE FIN DE LA RECHERCHE : V4 21
5.5 DUREE PREVUE DE PARTICIPATION DES PERSONNES, DESCRIPTION DE LA CHRONOLOGIE ET DE LA DUREE DE LA RECHERCHE. 21
5.6 TABLEAU RECAPITULATIF DE LA CHRONOLOGIE DE LA RECHERCHE 21
5.7 DISTINCTION SOIN-RECHERCHE 22
5.8 REGLES D'ARRET 22

6 CRITERES D'ELIGIBILITE 24

6.1 CRITERES D'INCLUSION 24
6.2 CRITERES DE NON INCLUSION 24
6.3 MODALITES DE RECRUTEMENT 24

7 DISPOSITIF MEDICAL UTILISE DANS LA RECHERCHE ET TRAITEMENTS, PROCEDURES, STRATEGIES ASSOCIES A L'UTILISATION DU DISPOSITIF 25

7.1 DESCRIPTION DES DISPOSITIFS MEDICAUX 25
7.2 DESCRIPTION DES TRAITEMENTS NON EXPERIMENTAUX 30
7.3 DESCRIPTION DES ELEMENTS DE TRAÇABILITE QUI ACCOMPAGNENT LE OU LES MEDICAMENTS EXPERIMENTAUX 30
7.4 TRAITEMENTS (MEDICAMENTEUX, NON MEDICAMENTEUX, CHIRURGICAUX) AUTORISES ET INTERDITS, Y COMPRIS LES MEDICAMENTS DE SECOURS 30
7.5 METHODES DE SUIVI DE L'OBSERVANCE AU TRAITEMENT 30

8 EVALUATION DE L'EFFICACITE 30

8.1 DESCRIPTION DES PARAMETRES D'EVALUATION 31
8.2 METHODES ET CALENDRIER PREVUS POUR MESURER, RECUEILLIR ET ANALYSER LES PARAMETRES D'EVALUATION DE L'EFFICACITE 31

9 COMITES SPECIFIQUES DE LA RECHERCHE 32

9.1 COMITE SCIENTIFIQUE 32

9.2 COMITE DE PILOTAGE 32
9.3 COMITE DE VALIDATION DES EVENEMENTS CRITIQUES 32

10 EVALUATION DE LA SECURITE – RISQUES ET CONTRAINTES AJOUTES PAR LA RECHERCHE 32

10.1 DESCRIPTION DES PARAMETRES D'EVALUATION DE LA SECURITE 32
10.2 METHODES ET CALENDRIER PREVUS POUR MESURER, RECUEILLIR ET ANALYSER LES PARAMETRES D'EVALUATION DE LA SECURITE 33
UNITE DE SURVEILLANCE POST-INTERVENTIONELLE : 33
IRM MULTIPARAMETRIQUE DE LA PROSTATE : 33
EVALUATION DE LA TOLERANCE SEXUELLE : 33
EVALUATION DE LA TOLERANCE URINAIRE : 33
10.3 DEFINITIONS 33
10.4 ROLES DE L'INVESTIGATEUR 35
10.5 ROLES DU PROMOTEUR 38
10.6 COMITE DE SURVEILLANCE INDEPENDANT 41

11 GESTION DES DONNEES 41

11.1 MODALITES DE RECUEIL DES DONNEES 42
11.2 IDENTIFICATION DES DONNEES RECUEILLIES DIRECTEMENT DANS LES CRF QUI SERONT CONSIDEREES COMME DONNEES-SOURCE 42
11.3 DROIT D'ACCES AUX DONNEES ET DOCUMENTS SOURCE 42
11.4 TRAITEMENT DES DONNEES ET CONSERVATION DES DOCUMENTS ET DES DONNEES 43
11.5 PROPRIETE DES DONNEES 44

12 ASPECTS STATISTIQUES 44

12.1 HYPOTHESES DE CALCUL DU NOMBRE DE SUJETS NECESSAIRES ET RESULTAT 44
12.2 DESCRIPTION DES METHODES STATISTIQUES PREVUES Y COMPRIS DU CALENDRIER DES ANALYSES INTERMEDIAIRES PREVUES 44

13 CONTROLE ET ASSURANCE DE LA QUALITE 44

13.1 ORGANISATION GENERALE 45
13.2 CONTROLE DE QUALITE 45
13.3 CAHIER D'OBSERVATION 46
13.4 GESTION DES NON CONFORMITES 46
13.5 AUDIT / INSPECTIONS 46
13.6 ENGAGEMENT DE RESPONSABILITES DE L'INVESTIGATEUR PRINCIPAL 46

14 ASPECTS ETHIQUES ET LEGAUX 47

14.1 MODALITES D'INFORMATION ET DE RECUEIL DU CONSENTEMENT DES PERSONNES SE PRETANT A LA RECHERCHE 47
14.2 INTERDICTION POUR LA PERSONNE DE PARTICIPER A UNE AUTRE RECHERCHE OU PERIODE D'EXCLUSION PREVUE A L'ISSUE DE LA RECHERCHE, LE CAS ECHEANT 47
14.3 INDEMNISATION DES SUJETS 47
14.4 AUTORISATION DES LIEUX 47

15 OBLIGATIONS LEGALES 48

15.1 ROLE DU PROMOTEUR 48
15.2 DEMANDE D'AVIS AU COMITE DE PROTECTION DES PERSONNES CPP 48
15.3 DEMANDE D'AUTORISATION A L'ANSM 48
15.4 ENGAGEMENT DE CONFORMITE A LA « METHODOLOGIE DE REFERENCE » MR 001 48
15.5 MODIFICATIONS DE LA RECHERCHE 48
15.6 RAPPORT FINAL DE LA RECHERCHE 48

16 FINANCEMENT ET ASSURANCE 48

16.1 SOURCE DE FINANCEMENT 49
16.2 ASSURANCE 49

17 REGLES RELATIVES A LA PUBLICATION 49

17.1 MENTION DE L'AFFILIATION DE L'AP-HP POUR LES PROJETS PROMUS PAR L'AP-HP 49
17.2 MENTION DU PROMOTEUR AP-HP (DRCI) DANS LES "ACKNOWLEDGMENTS" DU MANUSCRIT 49
17.3 MENTION DU FINANCEUR DANS LES "ACKNOWLEDGMENTS" DU MANUSCRIT 49

18 BIBLIOGRAPHIE 50

19 LISTE DES ADDENDA 53

19.1 LISTE DES INVESTIGATEURS 53
19.2 FORMULAIRE DE NOTIFICATION DES EVENEMENTS INDESIRABLES GRAVES 54
19.3 MARQUAGES CE 58
19.4 QUESTIONNAIRE IPSS (QUESTIONS 1 A 7) 63
19.5 QUESTIONNAIRE IPSS-QDV (SCORE DE QUALITE DE VIE, QUESTION 8) 64
19.6 QUESTIONNAIRE IIEF 64
19.7 QUESTIONNAIRE MSHG-EJD 66
19.8 ABAQUES 67
19.8.1 ABAQUE POUR LE THERAPIE FOCALE PAR ABLATION MICOR-ONDE 67
19.8.2 ABAQUE POUR LE TRAITEMENT MICRO-ONDE 78
19.9 CARTE PATIENT 80

1 RÉSUMÉ SYNOPTIQUE

Titre complet	Etude évaluant la faisabilité et la tolérance d'un traitement focal transrectal par micro-ondes de la tumeur index des patients ayant un cancer de la prostate à risque faible ou risque intermédiaire bas de progression
Acronyme	FOSTINE 01 (FOcal Secured Targeted Induced Energy)
Investigateur coordonnateur	Nicolas BARRY DELONGCHAMPS<br>Service d'urologie<br>Hôpital Cochin<br>Tél 01 58 41 27 64<br>Courriel : nicolas.barry-delongchamps@aphp.fr
Promoteur	Assistance Publique – Hôpitaux de Paris
Justification scientifique	Diminuer le risque de progression carcinologique et de recours à un traitement radical
Objectif et critère d'évaluation principal	Evaluer la faisabilité du traitement transrectal focal par micro-ondes d'une tumeur prostatique index identifiée par IRM, chez les patients ayant un cancer de la prostate à risque faible ou à risque intermédiaire bas de progression.<br><br>Le critère d'évaluation principal est la nécrose complète du volume cible sur l'IRM multiparamétrique de la prostate réalisée 7 jours après le traitement.
Objectifs et critères d'évaluation secondaires	1/ Evaluer les marges minimales et maximales de nécrose en dehors de la tumeur index, dans les trois plans de l'espace, en IRM multiparamétrique de la prostate réalisée 7 jours après le traitement.<br><br>2/ Evaluer la tolérance sexuelle après traitement, par auto-questionnaires IIEF (fonction érectile) et MSHQ-Ej (fonction éjaculatoire), réalisés à J7, 2 et 6 mois.<br><br>3/ Evaluer la tolérance urinaire après traitement par l'auto-questionnaire IPSS et IPSS-QDV, l'examen cytobactériologique des urines, le débit urinaire maximal, le Prostate Specific Antigen (PSA), et l'introduction de tout traitement additionnel à visée urinaire, à J7, 2 et 6 mois.<br><br>4/ Evaluer l'efficacité carcinologique après traitement par une IRM multiparamétrique de la prostate et des biopsies ciblées au niveau du volume traité, 6 mois après l'intervention.
Schéma expérimental	Etude monocentrique
Population concernée	Hommes adultes ayant un cancer de la prostate à risque faible ou risque intermédiaire bas de progression
Critères d'inclusion	- Patient âgé de 45 à 76 ans ;

	- Espérance de vie à l'inclusion de plus de 10 ans ;<br>- Diagnostic de cancer de la prostate confirmé par des biopsies transrectales de la prostate ;<br>- Classement dans le groupe à risque faible ou risque intermédiaire bas de progression de D'Amico, défini par :<br>*un stade clinique T1c ou T2a au toucher rectal<br>*un score de Gleason biopsique maximal de 3+4, y compris sur les biopsies ciblées correspondant à moins de 50% de grade 4 dans la tumeur<br>*un taux de Prostate Specific Antigen sérique <15 ng/mL<br>- Mise en évidence d'une zone tumorale en IRM multiparamétrique de la prostate, confirmée par des biopsies transrectales ciblées par fusion d'image IRM-échographie ;<br>- Patient acceptant d'être inclus dans un protocole de surveillance active à la fin de l'étude, conformément aux recommandations de bonne pratique ;<br>- Patient affilié à un régime de sécurité sociale ;<br>- Consentement libre, éclairé et écrit, daté et signé par le patient et l'investigateur, au plus tard le jour de l'inclusion et avant tout examen nécessité par l'étude.
Critères de non inclusion	- Antécédent de chirurgie de la prostate ;<br>- Antécédent de radiothérapie ou de traumatisme pelvien ;<br>- Antécédent de prostatite aiguë ou chronique documentée<br>- Antécédent d'allergie ou de non tolérance aux sels de Gadolinium utilisés en IRM ;<br>- Patient ayant une contre-indication à la réalisation d'une IRM<br>- Symptomatologie urinaire sévère liée à une hyperplasie bénigne de la prostate, et définie par un score IPSS >18 ;<br>- tumeur avec signes IRM d'extension extra-capsulaire ou d'envahissement des vésicules séminales.<br>- Mise en évidence d'un envahissement tumoral ayant une longueur supérieur à 3 mm sur les biopsies systématisées réalisées en dehors de la zone tumorale visible en IRM multiparamétrique de la prostate.<br>- Mise en évidence d'un grand axe tumoral supérieur à 20 mm en IRM multiparamétrique de la prostate ;<br>- Mise en évidence d'une distance de moins de 5 mm entre la circonférence tumorale et le rectum.<br>- Personne placée sous sauvegarde de justice.
Dispositif faisant l'objet de la recherche	*Phase initiale de développement du traitement focal micro-onde intra-prostatique par voie transrectale sous anesthésie générale*
Traitement de référence	*Prostatectomie radicale*
Autres actes ajoutés par la recherche	IRM multiparamétrique de la prostate à J7 après l'intervention
Risques ajoutés par la recherche	*Risque D*

Déroulement pratique	Les patients éligibles seront inclus. L'intervention sera organisée au moment de la visite d'inclusion et programmée dans un délai de 1 mois. L'intervention consistera en un traitement focal, transrectal, par micro-ondes de la tumeur prostatique index identifiée par IRM. Elle sera réalisée au bloc opératoire, sous anesthésie générale. Les patients seront autorisés à regagner leur domicile le lendemain de l'intervention. Une IRM multiparamétrique de la prostate sera réalisée 7 jours après l'intervention, puis une visite de contrôle aura lieu 2 mois et 6 mois après l'intervention. A 6 mois seront réalisés une IRM multiparamétrique de la prostate suivie de biopsies prostatiques ciblées dans la zone traitée.
Nombre de sujets sélectionnés	10
Nombre de centres	Un seul centre
Durée de la recherche	durée d'inclusion : 12 mois<br>durée de participation (traitement + suivi) : 7 mois maximum<br>durée totale : 19 mois
Nombre d'inclusions prévues par centre et par mois	Une inclusion par mois
Analyse statistique	Pour décrire l'efficacité du traitement : l'effectif et le pourcentage de patients ayant une nécrose complète du volume cible 7 jours après le traitement seront donnés. L'intervalle de confiance à 95% (en unilatéral) sera calculé via la méthode exacte de Clopper-Pearson.
Source de financement	*Société KOELIS*
Comité de Surveillance Indépendant prévu	Oui

2 JUSTIFICATION SCIENTIFIQUE DE LA RECHERCHE

Le dépistage du cancer de la prostate a permis la détection plus précoce de tumeurs localisées de petit volume et de faible agressivité [1]. La prise en charge de ces formes à risque faible ou risque intermédiaire bas de progression évolue aujourd'hui vers une désescalade thérapeutique. Les traitements radicaux du cancer de la prostate (chirurgie ou radiothérapie externe), du fait de leur possible retentissement important sur la qualité de vie ne sont en effet pas adaptés à ces patients sélectionnés. La prostatectomie radicale, en particulier, est grevée d'effets secondaires urinaires (incontinence) et sexuels significatifs [2]. La curiethérapie, de son côté entraîne une morbidité urinaire, transitoire dans la majorité des cas, mais qui peut gêner le patient pendant plusieurs mois [2]. Plus rarement, on peut observer une urétrite radique, complication redoutée et de traitement difficile touchant de 1 à 2% des patients [3].
Une surveillance active a donc été proposée pour ces patients, avec comme perspective de proposer un traitement radical en cas de progression.

Cependant, une alternative innovante actuellement en cours d'évaluation est de traiter de manière focale le foyer tumoral le plus agressif, dit tumeur index, afin de réduire le risque de progression, en évitant un traitement radical. La réalisation d'un traitement focal implique de localiser la tumeur et de focaliser l'agent thérapeutique sur la zone à traiter avec une grande précision.

Nous proposons de tirer parti de notre expertise en matière de traitement focal [4], de localisation de la tumeur par IRM [5, 6] et de biopsies ciblées [7-9] avec fusion d'images pour évaluer la faisabilité d'un traitement focal strictement limité à la zone tumorale (ou « ultra-focal ») du cancer par énergie micro-onde délivrée par voie transrectale. Les patients porteurs d'une tumeur unique visible sur l'IRM multiparamétrique seraient inclus dans ce protocole, une surveillance active étant ensuite mise en place pour dépister et traiter précocement une récidive dans la zone traitée ou un 2ème cancer prostatique métachrone.

2.1 Hypothèses de la recherche

Nous posons l'hypothèse de la faisabilité et de l'innocuité d'un traitement focal de la tumeur index, réalisé par micro-ondes délivrées par voie transrectale au moyen d'une aiguille, chez les patients ayant un cancer de la prostate à risque faible ou risque intermédiaire bas de progression et dont la tumeur index est identifiable en IRM multiparamétrique de la prostate.

2.2 Description des connaissances relatives à la pathologie concernée

Données épidémiologiques et dépistage du cancer de la prostate

L'incidence du cancer de la prostate est en constante augmentation en France depuis le début des années 1990 [10]. Aux Etats-Unis, il a été constaté un « pic » d'incidence au cours de la même période, suivi d'une augmentation de l'incidence jusqu'à nos jours [11]. L'hypothèse d'un lien entre l'augmentation de l'incidence et la pratique croissante du dosage du PSA a été évoquée, bien qu'elle n'ait pas été clairement démontrée. L'augmentation de la prévalence du cancer, elle-même dépendante de facteurs environnementaux, est aussi une hypothèse possible [12].

Depuis plusieurs années, l'association française d'urologie, de même que les sociétés savantes européennes et américaines, recommandent d'effectuer un dépistage individuel du cancer de la prostate une fois par an [13]. Ce dépistage s'adresse aux hommes âgés de 50

à 75 ans, c'est-à-dire ceux ayant une espérance de vie de plus de 10 ans. Il est recommandé de débuter ce dépistage dès l'âge de 45 ans en cas de risque familial (antécédents familiaux de cancer de la prostate) ou ethnique (populations d'origine africaine ou antillaise). Le dépistage repose sur deux outils, le toucher rectal (TR) et le dosage du PSA total. Ce dépistage fait néanmoins l'objet de nombreuses controverses, car il implique d'être complété par une série de 12 biopsies de prostate systématiques qui comportent un risque désormais établi de « sur-détection » et donc de « sur-traitement » des cancers dits à risque faible ou risque intermédiaire bas de progression.

Classification pronostique et traitements recommandés

Il est recommandé de distinguer trois groupes pronostiques selon la classification dite de D'AMICO qui s'applique à toutes les modalités thérapeutiques [14]. Le groupe de bon pronostic, dit à risque faible de progression [stade clinique T1c ou T2a, Score de Gleason 3+3, PSA <10ng/ml] peut être pris en charge par surveillance active, radiothérapie externe ou interstitielle ou chirurgie d'exérèse [15].]. Le groupe à risque intermédiaire bas [stade clinique T2a, score de Gleason 3+4, PSA<15ng/mL] ne peut être pris en charge par surveillance active, mais peut bénéficier de toutes les thérapies actives, en particulier le moins invasif d'entre eux qui est la radiothérapie interstitielle. Si elles sont également efficaces, avec un risque de décès spécifique de moins de 1% à 15 ans, aucune n'est totalement dénuée de conséquences sur la qualité de vie mictionnelle et sexuelle [16].

De son côté, la surveillance active, qui impose une réévaluation clinique (toucher rectal), biologique (PSA) et surtout biopsique régulière, est source d'angoisse [17] et comporte un risque infectieux minime mais réel associé à la biopsie (hospitalisation pour complication de l'ordre de 3,8% dans les 60j après la biopsie). Ainsi plus d'un tiers des patients inclus dans les protocoles de surveillance demande à en sortir à moyen terme [18] et sollicite un traitement radical.

Développement de l'IRM multiparamétrique de la prostate et des outils de guidage

2.2.1.1 IRM multiparamétrique

L'IRM multiparamétrique de la prostate, qui combine une séquence morphologique traditionnelle pondérée en T2, une séquence de diffusion et une séquence dynamique après injection intraveineuse de Gadolinium, a montré sa sensibilité élevée, variant de 71 à 86%, pour détecter et caractériser les foyers tumoraux de plus de 0,2 cm3 [19-24], en particulier chez les patients ayant un cancer à risque faible ou risque intermédiaire bas de D'Amico [8, 9, 22]. La combinaison des séquences morphologiques et fonctionnelles, dominées par la diffusion, a en effet renforcé la fiabilité diagnostique de l'IRM, de même que sa valeur pour détecter la présence d'une tumeur agressive.

Actuellement, l'IRM multiparamétrique est réalisée dans le cadre des soins courants à la fois pour faire le bilan d'extension locorégional du cancer de la prostate (recherche d'une extension extra-capsulaire, d'un envahissement des vésicules séminales, d'une atteinte ganglionnaire pelvienne) et pour rechercher la ou les cibles tumorales. Elle est recommandée par les sociétés savantes [13] et réalisée avant les biopsies dans les centres experts, ce qui permet de guider les prélèvements des zones suspectes de cancer.

Les modalités de l'examen sont les suivantes :

Le choix de l'aimant (1,5 ou 3 Tesla) et de l'antenne (pelvienne seule ou combinée à une antenne endorectale) est fonction des habitudes de chaque centre. Les recommandations pour un niveau d'équipement minimum ont été rapportées (ESUR, PIRADS v2)

Séquence en T2:
L'acquisition T2 est réalisée le plus souvent en mode 2D avec, en fonction des centres, une antenne pelvienne combinée à une antenne endorectale ou une antenne pelvienne seule, à condition qu'elle comporte au moins 16 canaux. Une acquisition 3D volumique peut également être réalisée.

Séquence en diffusion:
Une séquence d'acquisition est réalisée en echo-planar (EPI), avec une épaisseur de coupe de 2,5-3,5 mm et trois valeurs de b (50-500-1000 s/mm²). Pour superposer les informations fournies par chaque séquence, les coupes ont une épaisseur et un centrage identiques

Séquence dynamique après injection de Gadolinium (IRM dynamique):
L'injection intraveineuse de Gadolinium se fait en bolus à la dose de 0.1 mmol/kg, suivie d'un bolus de 15 ml de sérum physiologique. Les acquisitions sont répétées pendant 2 à 5 mn avec une fréquence (résolution temporelle) inférieure à 15 secondes. Elles visent à détecter un rehaussement précoce d'un hyposignal visible en T2 ou en diffusion (tableau 2). Les coupes ont le même centrage et la même épaisseur que les coupes de la séquence pondérée enT2.

La définition d'une zone suspecte repose sur un score de probabilité attribué à chaque région d'intérêt (échelle de 1 à 5) sur la base des recommandations de l'European Society of Uro-Radiology et l'American College of Radiology (tableau 1) [23, 24]. Un score global sur une échelle de 1 à 5 est ensuite attribué qui tient compte d'une séquence dite dominante différente pour la zone périphérique et la zone de transition. Pour les lésions de la zone périphérique, la séquence dominante est la séquence de diffusion qui donne donc en règle le score global pour déterminer le niveau de probabilité de malignité. Pour les lésions de la zone de transition, le score global est en règle celui de la séquence T2, séquence dominante dans cette zone.

Tableau 1: score de l'imagerie T2, de l'imagerie de diffusion et de perfusion (dynamique) recommandé par l'European Society of Urogenital Radiology (ESUR) et l'American College of Radiology (ACR)

Séquence pondérée en écho de spin T2
Zone périphérique (ZP)
1. hypersignal homogène.
2. hyposignaux triangulaires, linéaires
3. plages géographiques mal limitées, non classable dans les catégories 2 ou 4-5.
4. hyposignal avec effet de masse d'aspect confiné à la prostate, de grand axe ≤15mm.
5. similaire au 4, mais avec extension extra-capsulaire ou de grand axe >15mm

Zone de transition (ZT)
1. Aspect hétérogène de la ZT sans anomalie focale décelable.
2. Nodule encapsulé, en hyposignal homogène ou hétérogène.
3. Plage en hyposignal hétérogène et mal limitée non classable dans les catégories 4 ou 5.
4. Plage en hyposignal homogène, mal limitée, antérieure et souvent de forme lenticulaire, de grand axe ≤15mm
5. Même aspect que 4, mais avec atteinte du stroma fibro-musculaire antérieur ou de la corne latérale de la ZP, ou de grand axe >15mm.

Séquence pondérée en diffusion (ZP et ZT)

1. aspect homogène sans chute du Coefficient Apparent de Diffusion (ADC) par rapport au tissu normal. Pas d'augmentation de l'intensité de signal sur les images obtenues avec un gradient de diffusion élevé (valeur de b ≥1400)
2. hyposignal diffus sur la cartographie ADC avec augmentation diffuse de l'intensité de signal sur les images obtenues à b ≥1400. Pas d'anomalie focale, sauf si de forme triangulaire ou linéaire
3. plage en hyposignal modéré sur la carte ADC et en isosignal ou hypersignal modéré sur les images obtenues à b ≥1400c
4. hyposignal focal de grand axe ≤15mm sur la cartographie ADC et en hypersignal sur les images obtenues à b ≥1400,
5. hyposignal focal de grand axe >15mm sur la cartographie ADC et en hypersignal sur les images obtenues à b ≥1400,

Imagerie de contraste après injection de Gadolinium. (ZP)

1. réhaussement focal, précoce ou contemporain correspondant à un hyposignal visible sur la séquence pondérée en T2 ou en diffusion. *

0. pas de rehaussement précoce ou rehaussement diffus ne correspondant pas à une anomalie focale sur la séquence pondérée en T2 et/ou en diffusion, ou rehaussement focal dans un nodule d'allure hyperplasique sur la séquence pondérée en T2.

Tableau 2: Score PIRADS appliqué à la zone périphérique. *Score 1 : lésion significative très peu probable Score 2 : lésion significative peu probable*
Score 3 : lésion significative ne pouvant être éliminée Score 4 : lésion significative probable
Score 5 : lésion significative très probable

Diffusion	T2	Perfusion	global
Score 1	Indifférent*	indifférent	Score 1
Score 2	indifférent	indifférent	Score 2
Score 3	indifférent	Négative (0)<br>Positive (1)	Score 3<br>Score 4
Score 4	indifférent	indifférent	Score 4
Score 5	indifférent	indifférent	Score 5

* : quel que soit le score (de 1 à 5)

Tableau 3: Score PIRADS appliqué à la zone de transition
Score *1 : lésion significative très peu probable Score 2 : lésion significative peu probable*
Score 3 : lésion significative ne pouvant être éliminée Score 4 : lésion significative probable
Score 5 : lésion significative très probable

T2	Perfusion	Diffusion	PIRADS global
Score 1	indifférent	indifférent	Score 1
Score 2	indifférent	indifférent	Score 2
Score 3	indifférent	Score 3<br>Score 4	Score 3<br>Score 4
Score 4	indifférent	indifférent	Score 4
Score 5	indifférent	indifférent	Score 5

2.2.1.2 **Outils de guidage : le système Trinity de Koelis**

Le développement des techniques de fusion d'images entre l'IRM et l'échographie permet de cibler par échographie les zones suspectes visibles sur l'IRM multiparamétrique. Plusieurs systèmes ont été commercialisés, dont le système Trinity® (Koelis, Grenoble, France), qui a été évalué cliniquement et a fait la preuve de sa fiabilité pour guider une biopsie de prostate [7-9, 25-28.] avec une précision moyenne d'environ 3mm pour atteindre une cible de 0,5 cm3. [29].

Actuellement, ce système certifié CE est utilisé dans le cadre des soins courants pour réaliser les biopsies ciblées de la prostate. Son utilisation augmente un peu la durée des prélèvements sans modifier notablement le protocole classique de biopsie. La courbe d''apprentissage de la technique est accessible à tout praticien formé aux biopsies de prostate écho-guidées. La procédure de fusion d'image est détaillée dans le manuel utilisateur fourni en annexe.

En résumé, l'IRM multiparamétrique de la prostate, ainsi que la réalisation de biopsies ciblées guidées par fusion d'image IRM-échographie, permettent actuellement d'améliorer la caractérisation du cancer de la prostate, en précisant avec une meilleure fiabilité le score de Gleason du cancer, son volume et sa localisation.

2.3 Résumé des expérimentations pré cliniques et des essais cliniques concernés

Le traitement focal reste aujourd'hui en cours d'évaluation, sa réalisation posant en effet trois questions encore imparfaitement résolues : comment définir avec précision la zone à traiter, comment focaliser l'agent thérapeutique sur cette cible et enfin comment surveiller en toute sécurité le reste de la glande après traitement, compte tenu de l'existence possible de foyers tumoraux infra-cliniques non détectés [30].

A ce jour, plusieurs modes d'ablation tissulaire ont été testés, dans le cadre de traitements zonaux de la prostate ou d'hémi-ablation. La plupart d'entre elles utilisent le guidage échographique. La cryothérapie [31, 32] par voie tranpérinéale et les ultrasons focalisés par voie externe transabdominale [33-35] ont été utilisés dans quelques centres experts. Les courtes séries publiées ont rapporté des résultats intéressants [30, 34]. Plus récemment, la photothérapie dynamique de la prostate par voie tranpérinéale, utilisant un photosensibilisant (le Tookad) injecté par voie intraveineuse, a été testée chez les patients ayant un cancer de la prostate à faible risque de progression, défini par les résultats d'un protocole classique de 10-12 biopsies systématiques. Plusieurs études de phase II ont suggéré la faisabilité et la bonne tolérance urinaire et sexuelle de cette approche [36, 37]. Une étude internationale multicentrique de phase III, comparant cette technique à la surveillance active et à laquelle nous avons participé, vient de se terminer. Les résultats sont en cours de publication.

Cependant, l'absence de recalage d'image entre l'échographie et l'IRM lors de l'application du traitement, introduit une incertitude inquiétante sur la précision du guidage. De plus, la prostate est un organe extrêmement mobile et la simple insertion d'une sonde d'échographie et plus encore d'une aiguille entraine des déplacements pouvant aller jusqu'au centimètre, entrainant une imprécision majeure de ciblage. De fait, la délimitation imparfaite de la zone cible, fait que les traitements focaux actuellement réalisés consistent en une ablation de toute la partie droite ou gauche de la glande (hémi-ablation).

Pour augmenter la précision du geste ablatif, quelques équipes ont proposé la réalisation de traitements focaux sous guidage IRM en temps réel par voie tranpérinéale [38], ou transrectale [39], avec de bons résultats fonctionnels et carcinologiques à court terme. Néanmoins, la précision élevée de ces techniques est obtenue au détriment d'une durée d'examen de plusieurs heures, limitant la diffusion de ces traitements.

Nous proposons de dépasser ces obstacles en utilisant le système commercial de fusion d'image TRINITY (Koelis, Grenoble, France).

1) La définition de la zone cible se fera sur les résultats de l'imagerie IRM multiparamétrique (T2, Diffusion, Injection de contraste) les images IRM étant ensuite transférées vers le système TRINITY pour biopsies. Des régions d'intérêt seront ensuite délimitées sur l'IRM et recalées avec le volume échographique acquis avec une sonde 3D, ce système permettra, pendant les biopsies, d'archiver la localisation spatiale des trajets de l'aiguille par rapport à la région d'intérêt, et donc de la tumeur index à traiter.

2) Par ailleurs, la précision du système pour localiser une aiguille dans le volume prostatique [29] sera utilisée pour placer au cœur de la cible par voie transrectale, l'applicateur de la source de micro-ondes. Cet applicateur se présente sous la forme d'une aiguille de 18 Gauge, de diamètre identique à celui des aiguilles employées pour la réalisation des biopsies prostatiques dans le cadre des soins courants.

2.4 Description de la population à étudier et justification de son choix

La population incluse sera constituée d'hommes ayant une espérance de vie de plus de 10 ans, et atteints d'un cancer de la prostate localisé à faible risque ou risque intermédiaire bas de progression selon la définition de D'Amico (détaillée dans le chapitre 2.2.2). De plus, seules les tumeurs sans extension extra-prostatique infra-clinique et sur l'IRM multiparamétrique seront incluses.

Ces critères d'éligibilité définissent cette population qui peut tirer le bénéfice optimal d'un traitement focal, comme l'a considéré une conférence de consensus internationale [40].

2.5 Dénomination et description du dipositif médical

Le traitement à l'étude est ***l'ablation focale par micro-ondes***. Ce traitement sera délivré par un applicateur de 18 Gauge (TATOpro® 18G de la société Biomedical SRL) introduit sous guidage échographique par voie transrectale, et relié à un générateur (TATO® de Biomedical). Les caractéristiques techniques et le guide utilisateur de l'applicateur et du générateur sont fournis en annexe.

Le guidage de cette aiguille de traitement sera réalisé par fusion d'image échographie-IRM grâce au système TRINITY® (Koelis, Grenoble, France), dont les caractéristiques sont détaillées en annexe.

Au cours du traitement, l'applicateur de micro-ondes sera maintenu, solidairement à la sonde d'échographie, par un bras mécanique dont les caractéristiques sont détaillées au chapitre 7. Ce bras permet de conserver la position et l'orientation de l'applicateur lorsque le générateur émet les ondes.

2.6 Description et justification des modalités d'utilisation du dispositif médical

L'administration des micro-ondes est détaillée au chapitre 7. La durée d'administration et la puissance délivrée par le générateur sont modulables. Ces paramètres seront adaptés au volume cible à traiter, selon un abaque préétabli fourni en annexe.

2.7 Résumé des bénéfices et des risques prévisibles et connus pour les personnes se prêtant à la recherche

2.7.1 Bénéfices prévisibles

Les bénéfices individuels prévisibles concernent le traitement focal de la tumeur index du cancer de la prostate. Comme précisé au chapitre 2.2.2., les traitements recommandés chez les patients ayant un cancer de la prostate à faible risque de progression s'étendent de la surveillance active à la prostatectomie totale.

Si l'on se positionne par rapport à la prise en charge la moins agressive (surveillance active), le bénéfice attendu est carcinologique. L'étude des cohortes de patients en cours de surveillance active suggère qu'environ un tiers des patients auront une progression carcinologique, le plus souvent au cours des 2-3 premières années [41]. Par ailleurs, il est globalement admis que la tumeur index est à l'origine de la progression carcinologique [13]. Traiter de manière focale la tumeur index devrait donc supprimer le risque de progression tumorale encouru avec la surveillance active, et diminuer le risque de recours à une prostatectomie radicale et à la morbidité à laquelle elle expose.

Si l'on se positionne par rapport à la prise en charge la plus agressive (prostatectomie totale), le bénéfice attendu est une diminution majeure de la morbidité induite. En effet, la prostatectomie totale est à l'origine d'une incontinence urinaire dans un tiers des cas et d'une dysfonction érectile chez plus de la moitié des patients [13]. Traiter focalement la tumeur index permet de préserver le reste de la glande, les bandelettes vasculo-nerveuses ainsi que le sphincter strié de l'urètre.

2.7.2 Risques prévisibles

Les risques prévisibles de la recherche sont liés d'une part à la voie d'abord transrectale, et d'autre part à l'utilisation des micro-ondes. Il n'y a pas de risque à l'utilisation du module de guidage et de navigation TRINITY.

2.7.2.1 Risque fonctionnel urinaire et sexuel

Les risques prévisibles d'un traitement focal ablatif de la prostate sont liés à la diffusion de l'énergie en dehors de la glande, au niveau du rectum, de l'urètre, ainsi que des bandelettes vasculo-nerveuses.
Les séries publiées de traitement focal par cryothérapie, ultrasons focalisés, ou photothérapie dynamique ont suggéré la bonne tolérance de ces traitements, alors même qu'il ne s'agissait pas de traitements limités à la tumeur index mais d'ablations tissulaires plus volumineuses, englobant la tumeur index et allant jusqu'à l'hémi-ablation [34, 36, 37].

Feijo et al [34] ont rapportés les résultats fonctionnels précoces de l'hémiablation par ultrasons focalisés chez 71 patients. A 3 mois, tous les patients étaient continents, et la

fonction érectile était maintenue chez 11 des 21 patients ayant une fonction érectile jugée normale en pré-opératoire. Les auteurs ont rapporté la survenue de complications de grade 3 dans la classification de Clavien chez 2,8% des patients.

Une analyse combinée des résultats des études de phase II évaluant l'hémi-ablation prostatique par photothérapie dynamique après injection intraveineuse de Tookad est disponible : Azzouzi et al [36, 37] ont rapporté les résultats fonctionnels urinaires et sexuels à 6 mois chez 117 patients. Les scores urinaires sont restés stables au cours du temps. Le score érectile (IIEF5) a légèrement diminué, mais de manière non significative.

A notre connaissance, une seule série sur le traitement focal limité à la tumeur index a été publiée : Lepor et al [39] ont rapportés chez 25 patients les résultats fonctionnels à 3 mois d'un traitement focal de la tumeur index par laser interstitiel délivré par voie transrectale sous guidage IRM en temps réel. Les scores fonctionnels urinaires et sexuels étaient inchangés par rapport à la période préopératoire.

Dans la recherche proposée, nous prévoyons des suites fonctionnelles, liées à la diffusion de l'énergie, comparables à celles rapportées par Lepor et al [39], et donc un risque non significatif. D'après les abaques de traitement micro-onde, la diffusion de l'énergie est reproductible, l'effet comète négligeable, et la zone de nécrose tissulaire engendrée est donc prévisible à une puissance et une durée de traitement données. L'effet thermique est donc au maximum équivalent à celui engendré par un laser interstitiel comme celui utilisé dans l'étude de Lepor et al [39]. Enfin, un contrôle en temps réel de la position de l'applicateur permettra de mesurer précisément la distance de la zone de traitement par rapport au rectum.

2.7.2.2 Risque infectieux

Dans la recherche proposée, le risque infectieux est lié à la voie d'abord transrectale. Ce risque est largement évalué dans la littérature chez les patients soumis à des biopsies de la prostate par voie transrectale, plus particulièrement chez les patients sans cancer et en cas de biopsie répétée lors de la surveillance. Une étude canadienne de registre a été réalisée chez 37 190 patients ayant eu des biopsies prostatiques. Cette étude a mis en évidence un taux d'hospitalisation pour complication dans les 30 jours suivant l'acte de 1,9 % [42]. Le taux des complications infectieuses sévères et de rétention aiguë d'urines rapportées après des biopsies prostatiques réalisées par voie transrectale représente moins de 1 %. Il est limité par la prescription d'une antibiothérapie prophylactique préalable au geste et pouvant se poursuivre entre 24 et 72 heures [13, 43].

Complications	Fréquence (%)
Hémospermie	37,4
Urétrorragie > 1 jour	14,5
Fièvre	0,8
Sepsis	0,3
Rectorragie	2,2
Rétention aiguë d'urines	0,2
Prostatite	1
Épididymite	0,7

Tableau 1 : Complications rapportées après biopsies prostatiques systématisées écho-guidées par voie transrectale [15]

Lepor et al. [39], ne rapportent pas de cas d'infection après ablation focale par voie transrectale sous guidage IRM.

Dans la recherche proposée, nous estimons donc que le risque lié à la voie d'abord transrectale est minime. Par rapport à la voie transpérinéale, la voie d'abord trans-rectale est réputée moins invasive et douloureuse. Le risque prévisible de l'utilisation de la voie trans-rectale dans la recherche proposée est donc identique à celui des biopsies de la prostate, utilisée en soins courants depuis plus de 30 ans. L'applicateur micro-onde a en effet un diamètre identique à l'aiguille utilisée pour les biopsies de la prostate (18 gauges). La lésion muqueuse engendrée sur le trajet de ponction sera donc du même ordre.

2.7.2.3 Rapport Bénéfices / Risques

Compte tenu des bénéfices attendus et des risques prévisibles, le traitement à l'étude semble se situer à mi-chemin entre une surveillance active et une prostatectomie radicale, ces deux formes de prise en charge étant admises en pratique clinique dans la population soumise à la recherche proposée.

3 OBJECTIFS

3.1 Objectif principal

Evaluer la faisabilité du traitement transrectal ablatif focal par micro-ondes d'une tumeur prostatique index identifiée par IRM multiparamétrique, chez les patients ayant un cancer de la prostate à faible risque de progression selon la classification de d'Amico.

3.2 Objectifs secondaires

1/ Evaluer le degré d'extension du traitement transrectal focal par micro-ondes de la tumeur index prostatique ;

2/ Evaluer la tolérance sexuelle du traitement transrectal focal par micro-ondes de la tumeur index prostatique ;

3/ Evaluer la tolérance urinaire du traitement transrectal focal par micro-ondes de la tumeur index prostatique ;

4/ Evaluer l'évolution carcinologique à 6 mois du traitement transrectal focal par micro-ondes de la tumeur index prostatique.

4 CONCEPTION DE LA RECHERCHE

4.1 Enoncé précis des critères d'évaluation principal et secondaires

Critère d'évaluation principal

Le critère d'évaluation principal est la nécrose complète du volume cible défini sur l'IRM avant traitement. Cette nécrose sera évaluée sur une IRM multiparamétrique réalisée 7 jours après le traitement.

Critères d'évaluation secondaires

1/ Le degré d'extension du traitement sera défini sur l'IRM à J7 après traitement par la longueur (en millimètres) des marges minimales et maximales de nécrose en dehors du volume cible, dans les trois plans de l'espace
2/ La tolérance sexuelle sera évaluée par les auto-questionnaires IIEF (fonction érectile) et MSHQ-Ej (fonction éjaculatoire) (fournis en annexe), réalisés à J7, 2 et 6 mois.

3/ La tolérance urinaire sera évaluée par les auto-questionnaires IPSS et IPSS-QDV (fournis en annexe), l'examen cytobactériologique des urines, le débit urinaire maximal, le Prostate Specific Antigen (PSA), et l'introduction de tout traitement additionnel à visée urinaire, à J7, 2 et 6 mois.

4/ L'évolution carcinologique sera évaluée par une IRM multiparamétrique de la prostate et des biopsies ciblées au niveau du volume traité, 6 mois après l'intervention.

4.2 Description de la méthodologie de la recherche

Plan expérimental
Cette recherche est une étude interventionnelle, ouverte et non comparative

Nombre de centres participants
La recherche est monocentrique. Le service d'urologie de l'hôpital Cochin participera à la recherche et sera à l'origine du recrutement des patients.

Identification des sujets
Dans le cadre de cette recherche, les sujets seront identifiés de la façon suivante :
n° centre (3 positions numériques) – n°ordre de sélection de la personne dans le centre (4 positions numériques) - initiale nom - initiale prénom
Cette référence est unique et sera conservée pour toute la durée de la recherche.

5 DÉROULEMENT DE LA RECHERCHE

Avant tout examen ou acte lié à la recherche, l'investigateur recueille le consentement libre, éclairé et écrit de la personne qui se prête à la recherche ou de son représentant légal le cas échéant.

5.1 Visite de sélection : V-1

La visite de sélection a lieu entre 2 *mois* et au plus tard *3 jours* avant la visite d'inclusion.
Cette visite a lieu en consultation.

Au cours de cette visite, l'investigateur :

- Réalisera un examen clinique comportant un toucher rectal, et précisant la présence ou non d'un nodule prostatique suspect.
- Vérifiera le taux de PSA, dosé dans le cadre des soins courants et datant de moins de 3 mois.
- Vérifiera que l'IRM multiparamétrique de la prostate datant de moins de 2 mois et réalisée dans le cadre des soins courants, a localisé une cible suspecte correspondant à la tumeur en précisant son siège exact, incluant ses rapports avec le sphincter distal et l'urètre prostatique ainsi que son grand axe. L'absence d'extension extra-capsulaire et aux vésicules séminales de la tumeur, ainsi que l'absence d'adénopathie pelvienne suspecte.
- Vérifiera le résultat des biopsies ciblées de la prostate réalisées dans le cadre des soins courants : ces biopsies devront avoir été dirigées dans la zone suspecte décrite en IRM multiparamétrique de la prostate par fusion d'image IRM-échographie utilisant le système Koelis Grenoble, France).
- Vérifiera l'absence de tumeur significative (définition UCL) sur les biopsies systématiques des sextants d'aspect normal en IRM
- Vérifiera la stérilité des urines sur l'examen cytobactériologique réalisé quelques jours avant dans le cadre des soins courants.
- Vérifiera les critères d'éligibilité puis informera le patient de la recherche.
- Fournira au patient la fiche d'information concernant la recherche, et informera le patient du délai de réflexion de 3 jours avant la possibilité d'une inclusion dans l'étude. Ce délai devra être respecté avant la signature de la feuille de consentement éclairé.

Personnes dont le consentement est sollicité	Qui informe et recueille le consentement de la personne	A quel moment la personne est informée	A quel moment le consentement de la personne est recueilli
La personne qui se prête à la recherche	• *l'investigateur (urologue)*<br>• *son représentant*	*Visite de sélection et visite d'inclusion*	*Après un délai de réflexion de 3 jours*

5.2 Visite d'inclusion : V0

La visite d'inclusion a lieu au plus tôt 3 jours après la visite de sélection, et au plus tard 2 mois après.
Cette visite a lieu en consultation.

Au cours de cette visite, l'investigateur :

- Vérifiera les critères d'inclusion et de non inclusion ;
- Fournira au patient un complément d'information concernant la recherche ;
- Fera signer au patient la fiche de consentement éclairé ;
- Réalisera un examen clinique comportant au minimum un toucher rectal;
- Réalisera une débitmétrie avec mesure du débit maximal ;
- Fera remplir au patient les auto-questionnaires IIEF, MSHQ-Ej, IPSS et IPSS-QDV ;
- Organisera la visite d'intervention, qui devra être programmée dans un délai de 1 mois, au cours d'une hospitalisation dans le service d'urologie de l'hôpital Cochin :
    - Bilan sanguin préopératoire, tel que pratiqué dans le cadre des soins courants
    - Consultation d'anesthésie

5.3 Visites de suivi de la recherche

Visite d'intervention : V1

La visite d'intervention a lieu dans un délai de 1 mois après la visite d'inclusion. L'intervention est réalisée au cours d'une hospitalisation au sein du service d'urologie de l'hôpital Cochin.
Au cours de cette visite sera réalisé le traitement à l'étude, tel que décrit dans le chapitre 7 du protocole.

Visite du 7ème jour post intervention : V2

Cette visite sera réalisée en consultation, par un urologue, 7 jours après la visite d'intervention. Un intervalle de 5 jours supplémentaires sera toléré pour la réalisation de cette visite.

Au cours de cette visite, le médecin :

- Réalisera un examen clinique, comportant au minimum :
  - La prise de température ;
  - Un toucher rectal ;
- Vérifiera l'absence de survenue d'évènement indésirable ;
- Vérifiera la stérilité des urines sur l'examen cytobactériologique des urines réalisé trois jours plus tôt ;
- Réalisera une débitmétrie avec mesure du débit maximal ;
- Informera le patient du résultat de l'IRM multiparamétrique de la prostate réalisé sept jours après la visite d'intervention;
- Récupèrera les auto-questionnaires IIEF, MSHQ-Ej, IPSS et IPSS-QDV préalablement remplis par le patient, et lui fournira les auto-questionnaires à remplir pour la prochaine visite de suivi.
- Informera le patient de la suite de la prise en charge, et de la prochaine visite de suivi

Visite à 2 mois : V3

Cette visite sera réalisée en consultation, par un urologue, 2 mois après la visite d'intervention. Un intervalle de plus ou moins 15 jours sera toléré pour la réalisation de cette visite.

Au cours de cette visite, le médecin :

- Réalisera un examen clinique, comportant au minimum : un toucher rectal ;
- Vérifiera l'absence de survenue d'évènement indésirable ;
- Réalisera une débitmétrie avec mesure du débit maximal ;
- Réalisera un prélèvement sanguin pour mesure du taux de Prostate Specific Antigen (PSA) ;
- Récupèrera les auto-questionnaires IIEF, MSHQ-Ej, IPSS et IPSS-QDV préalablement remplis par le patient, et lui fournira les auto-questionnaires à remplir pour la prochaine visite de suivi.
- Informera le patient de la suite de la prise en charge, et de la prochaine visite de suivi

A la fin de la visite, l'investigateur programmera la réalisation d'une IRM multiparamétrique de la prostate et de biopsies ciblées à 6 mois, telles que prévues dans le cadre des soins courants en cas de surveillance active.

5.4 Visite de fin de la recherche : V4

Cette visite sera réalisée en consultation, par un urologue, 6 mois après la visite d'intervention. Un intervalle de plus ou moins 15 jours sera toléré pour la réalisation de cette visite.

Au cours de cette visite, le médecin :

Réalisera un examen clinique, comportant au minimum :

Un toucher rectal ;

Vérifiera l'absence de survenue d'évènement indésirable ;

Réalisera une débitmétrie avec mesure du débit maximal ;

Réalisera un prélèvement sanguin pour mesure du taux de Prostate Specific Antigen (PSA) ;

Informera le patient du résultat de l'IRM multiparamétrique de la prostate et des biopsies ciblées réalisées quelques jours auparavant.

Récupèrera les auto-questionnaires IIEF, MSHQ-Ej, IPSS et IPSS-QDV préalablement remplis par le patient.

Informera le patient de la fin de la recherche et lui expliquera la suite de la prise en charge.

Les modalités de la prise en charge médicale des personnes prévue en fin de recherche sont celles relatives à la surveillance active du cancer de la prostate à risque faible ou risque intermédiaire bas de progression, telles que précisées dans les recommandations de bonne pratique [Salomon].

5.5 Durée prévue de participation des personnes, description de la chronologie et de la durée de la recherche.

Durée maximale entre la sélection et l'inclusion	2 mois
Durée de la période d'inclusion	12 mois
Durée de participation des sujets, dont :	7 mois maximum
• Durée du traitement :	2 à 20 minutes
• Durée du suivi :	6 mois +/- 15 jours
Durée totale de la recherche :	19 mois

5.6 Tableau récapitulatif de la chronologie de la recherche

	V-1	*V0*	*V1*	*V2*	*V3*	*V4*
Actions	*J-60 – J-3 jours (sélection)*	*J0* ***(inclusion)***	*J0 +/- 1 mois (intervention)*	Suivi à 7 jours	Suivi à 2 mois	Suivi à 6 mois (fin d'étude)
Fiche d'information	*X*					
Consentement éclairé		*X*				
Vérification critères éligibilité	*X*	*X*				
Toucher rectal	*X*	*X*		*X*	*X*	*X*
PSA	*X*				*X*	*X*
Débitmétrie		*X*		*X*	*X*	*X*

Auto-questionnaires		*X*		*X*	*X*	*X*
IRM multiparamétrique de la prostate	*X*			*X + 2 jours si nécessaire*		*X*
Biopsies ciblées de la prostate	*X*					*X*
*Bilan préopératoire**		*X*				
Dispensation du traitement			*X*			
Evénements indésirables			*X*	*X*	*X*	*X*

**Bilan préopératoire :* bilan sanguin comportant une numération-formule sanguine, un bilan d'hémostase, une double détermination de groupe sanguin et Rhésus, un ionogramme sanguin, un dosage de la créatininémie ; bilan urinaire comportant un examen cytobactériologique des urines.

5.7 Distinction soin-recherche

TABLEAU : Distinction entre les actes liés au « soin » et les actes ajoutés par la « recherche »

Actes, procédures et traitements réalisés dans le cadre de la recherche	Actes, procédures et traitements liés aux <u>soins</u>	Actes, procédures et traitements ajoutés par <u>la recherche</u>
**Traitements**		*Traitement transrectal micro-onde*
**Consultations**	*Consultation à 3 et 6 mois Incluant un toucher rectal et des auto-questionnaires*	*Consultations supplémentaires V0, V2, V3, (à l'inclusion, à J7 et 2 mois)*
**Prises de sang**	*PSA à 3 et 6 mois*	
**IRM de la prostate**	*IRM multiparamétrique à l'inclusion et à 6 mois*	*IRM multiparamétrique à J7*
**Biopsies**	*Biopsies de la prostate à l'inclusion et à 6 mois*	

5.8 Règles d'arrêt

Critères et modalités d'arrêt prématuré du traitement de la recherche

5.8.1.1 Différentes situations

- Arrêt temporaire du traitement, l'investigateur doit documenter la raison de l'arrêt et sa reprise dans le dossier source du sujet et le CRF
- Arrêt prématuré du traitement, mais le sujet reste dans la recherche, jusqu'à la fin de sa participation, l'investigateur doit documenter la raison
- Arrêt prématuré du traitement et arrêt de participation à la recherche.

L'investigateur doit :

  - Documenter la ou les raison(s)
  - Recueillir les critères d'évaluation au moment de l'arrêt de participation à la recherche, si le sujet est d'accord
  - Poursuivre le suivi du sujet, notamment en cas d'effet indésirable grave

5.8.1.2 Critères et modalités d'arrêt prématuré de la recherche

- Tout sujet peut arrêter sa participation à la recherche, à n'importe quel moment et quelle qu'en soit la raison.

- L'investigateur peut interrompre temporairement ou définitivement la participation d'un sujet à la recherche pour toute raison ayant un impact sur sa sécurité ou qui servirait au mieux les intérêts du sujet.
    - Sujet perdu de vue : on ne sait pas ce qu'est devenu le sujet. L'investigateur doit mettre tout en œuvre pour reprendre contact avec le sujet (et le tracer dans le dossier source) afin de savoir au moins si le sujet est vivant ou décédé

En cas d'arrêt prématuré de la recherche d'un sujet, ou de retrait du consentement, les données le concernant pourront être utilisées en l'absence d'opposition de celui-ci lors de la signature du consentement.

Le cahier d'observation doit lister les différentes raisons d'arrêt de participation à la recherche:

- ☐ Inefficacité
- ☐ Effet indésirable
- ☐ Autre problème médical
- ☐ Raison personnelle du sujet
- ☐ Retrait explicite de consentement

Suivi des sujets suite à un arrêt prématuré de traitement

L'arrêt de participation d'un sujet ne changera pas sa prise en charge habituelle par rapport à sa maladie.
La survenue d'un 'évènement indésirable grave sera notifiée par l'investigateur au promoteur et faire l'objet d'un suivi pendant les 12 mois suivant l'arrêt prématuré du traitement (à adapter en fonction de la recherche). Dans le cas d'un arrêt prématuré du traitement à la suite de la survenue d'un évènement indésirable grave, une notification d'évènement indésirable grave sera transmise par télécopie (01 44 84 17 99) au promoteur. L'effet indésirable grave sera suivi jusqu'à sa résolution.

Arrêt d'une partie ou de la totalité de la recherche

Le promoteur AP-HP ou l'Autorité Compétente (ANSM) peuvent interrompre prématurément de façon temporaire ou définitive toute ou une partie de la recherche, dans les situations suivantes:

- en premier, en cas d'effets indésirables graves inattendus (SUSARS) nécessitant une réévaluation du rapport bénéfices/risques de la recherche.
- de même, des faits imprévus, de nouvelles informations relatives au produit, au vu desquels les objectifs de la recherche ou du programme clinique ne seront vraisemblablement pas atteints, peuvent amener le promoteur AP-HP ou l'Autorité Compétente (ANSM) à interrompre prématurément la recherche.
- le promoteur AP-HP se réserve le droit de suspendre définitivement les inclusions, à tout moment, s'il s'avère que les objectifs d'inclusion ne sont pas atteints.

Dans tous les cas d'arrêt d'une recherche, les sujets inclus dans la recherche doivent être suivis jusqu'à la fin de leur participation, comme prévu par le protocole.

En cas d'arrêt prématuré de la recherche, la décision et la justification sont transmises par le promoteur AP-HP dans un délai de 15 jours à l'Autorité Compétente (ANSM) et au CPP.

6 CRITERES D'ELIGIBILITE

6.1 Critères d'inclusion

- Patient âgé de 45 à 76 ans ;
- Espérance de vie à l'inclusion de plus de 10 ans ;
- Diagnostic de cancer de la prostate confirmé par des biopsies transrectales de la prostate ;
- Classement dans le groupe à risque faible ou risque intermédiaire bas de progression de D'Amico, défini par :

*un stade clinique T1c ou T2a au toucher rectal
*un score de Gleason biopsique maximal de 3+4, y compris sur les biopsies ciblées correspondant à moins de 50% de grade 4 dans la tumeur
*un taux de Prostate Specific Antigen sérique <15 ng/mL

- Mise en évidence d'une zone tumorale en IRM multiparamétrique de la prostate, confirmée par des biopsies transrectales ciblées par fusion d'image IRM-échographie ;
- Patient acceptant d'être inclus dans un protocole de surveillance active à la fin de l'étude, conformément aux recommandations de bonne pratique ;
- Patient affilié à un régime de sécurité sociale ;
- Consentement libre, éclairé et écrit, daté et signé par le patient et l'investigateur, au plus tard le jour de l'inclusion et avant tout examen nécessité par l'étude.

6.2 Critères de non inclusion

- Antécédent de chirurgie de la prostate ;
- Antécédent de radiothérapie ou de traumatisme pelvien ;
- Antécédent de prostatite aiguë ou chronique documentée
- Antécédent d'allergie ou de non tolérance aux sels de Gadolinium utilisés en IRM ;
- Patient ayant une contre-indication à la réalisation d'une IRM
- Symptomatologie urinaire sévère liée à une hyperplasie bénigne de la prostate, et définie par un score IPSS >18 ;
- tumeur avec signes IRM d'extension extra-capsulaire ou d'envahissement des vésicules séminales.
- Mise en évidence d'un envahissement tumoral ayant une longueur supérieur à 3 mm sur les biopsies systématisées réalisées en dehors de la zone tumorale visible en IRM multiparamétrique de la prostate.
- Mise en évidence d'un grand axe tumoral supérieur à 20 mm en IRM multiparamétrique de la prostate ;
- Mise en évidence d'une distance de moins de 5 mm entre la circonférence tumorale et le rectum.
- Personne placée sous sauvegarde de justice.

6.3 Modalités de recrutement

Le recrutement des patients s'effectuera en consultation hospitalière, au sein du service d'urologie de l'hôpital Cochin. Toute inclusion potentielle dans l'étude sera au préalable discutée puis validée au cours de la réunion de concertation pluridisciplinaire du service d'urologie.

10 patients devront être recrutés de manière séquentielle pendant la période d'inclusion, ce qui est largement en deçà des capacités de recrutement du service d'urologie de l'hôpital Cochin. En effet, environ 12 patients potentiellement éligibles sont recrutés tous les mois.

	Nombre de sujets

Nombre total de sujets sélectionnés	24
Nombre de centres	1
Période d'inclusion (mois)	12
Nombre de sujets à inclure	10
**Nombre de sujets / mois à inclure**	**1**

7 DISPOSITIF MEDICAL UTILISE DANS LA RECHERCHE ET TRAITEMENTS, PROCEDURES, STRATEGIES ASSOCIES A L'UTILISATION DU DISPOSITIF

7.1 Description des dispositifs médicaux

Le traitement à l'étude est la thermo-ablation focale par micro-onde d'une tumeur prostatique, réalisée par voie transrectale, et guidée par fusion d'image IRM-échographie, sous anesthésie générale.

Dispositif expérimental faisant l'objet de la recherche

Le dispositif médical faisant l'objet de la recherche est un système d'ablation thermique par microondes TATO du fabricant Biomedical. L'applicateur utilisé est le TATOpro 18G spécialement adapté au traitement de la prostate.
Référence : TATO1
Numéro de série : BMEM03aaxx

Destination prévue du dispositif

L'utilisation du dispositif dans le cadre de la recherche est tel que prévu dans le manuel utilisateur du dispositif :
« Le système ablation thermique TATO est utilisé pour la coagulation percutanée, laparoscopique et intraopératoire des tissus mous (foie, poumon, rein et prostate), y compris l'ablation partielle ou complète des tumeurs non opérables. Il s'agit d'une procédure d'hyperthermie extrême visant à la destruction des masses tumorales profonde grâce à l'application de l'énergie électromagnétique via une aiguille de très petite taille, entre 18 et 11 G, dit "applicateur", insérée dans le corps du patient jusqu'à atteindre la masse tumorale, en général par guidage échographique. »

Modalités d'utilisation du dispositif

L'ensemble des instructions se trouve dans le manuel utilisateur en annexe.

- Le rayonnement micro-onde du système peut être la source d'interférences électromagnétiques avec d'autres dispositifs médicaux. Dans de tels cas, il peut être nécessaire d'éloigner les dispositifs.
- L'applicateur doit être connecté au générateur avant d'être inséré dans le patient.
- L'énergie microonde ne doit être activée que lorsque l'applicateur est pleinement inséré sur la zone cible.
- L'applicateur est jetable et à usage unique.
- A la fin de la procédure, l'applicateur peut rester chaud. Il ne faut donc pas toucher l'applicateur après utilisation, et ne pas l'approcher de matériaux inflammables.

Modalités de formation à l'utilisation du dispositif

Une formation à la manipulation, au stockage, et à l'utilisation du dispositif TATO sera réalisée au bloc opératoire PR1 de l'hôpital Cochin, dans la salle d'intervention qui sera utilisée pour la recherche, avant la visite de mise en place. Un ingénieur Biomédical assisté d'un technicien formera l'ensemble de l'équipe, incluant les Dr Nicolas Barry Delongchamps

et Alexandre Schull, le personnel IBODE et le personnel d'encadrement du bloc opératoire (Monsieur Melton Momperousse, cadre supérieur).
Cette formation inclura en particulier:
La stérilisation de l'applicateur micro-onde ;
La mise en route du générateur micro-onde ;
Les réglages de la puissance et de la durée d'émission de l'énergie micro-onde ;
La procédure d'arrêt en urgence de l'émission de l'énergie micro-onde.

Description des autres dispositifs médicaux utilisés pour les besoins de la recherche

Les dispositifs médicaux suivants seront utilisés pour assister le système microonde qui fait l'objet de la recherche :

Le système d'imagerie et de guidage de KOELIS, qui comprend :
- La Station TRINITY
- Le Logiciel PROMAP
- Une sonde échographique endocavitaire
- Un guide porte-instrument réutilisable de KOELIS

- Enfin, la sonde d'échographie et son guide seront maintenus par un bras articulé de FISSO pendant la procédure afin d'éviter tout mouvement lors de l'émission des micro-ondes.

Préparation à l'intervention

7.1.1.1 Consultation d'anesthésie

Chaque patient inclus aura une consultation d'anesthésie en vue de l'intervention, programmée au sein du service d'urologie de l'hôpital Cochin au minimum 3 jours avant le geste chirurgical. Au cours de cette consultation, l'anesthésiste vérifiera le bilan préopératoire prescrit au cours de la visite d'inclusion (chapitre 5.6) et réalisera un électrocardiogramme. L'intervention sera réalisée sous anesthésie générale.

7.1.1.2 Antibioprophylaxie et lavement rectale

Conformément aux recommandations de l'association européenne d'urologie [Naber], une antibioprophylaxie par ofloxacine sera débutée 2 heures avant l'intervention et prolongée pendant 48 heures, à la posologie de 200 mg matin et soir. En cas d'allergie, une antibioprophylaxie par Trimétoprime-Sulfamétoxazole (800 mg / 160 mg) sera prescrite à la posologie de deux comprimés matin et soir pendant 48 heures.

Un lavement rectal sera réalisé la veille ainsi que 2 heures avant l'intervention (une dose de Microlax).

Installation du patient

Le patient sera installé en décubitus dorsal, et en position gynécologique.

Voie d'abord

La voie d'abord est transrectale et écho-guidée, comme pour une biopsie de prostate. Un lavement évacuateur (Microlax) est d'abord effectué la veille et le matin de l'intervention. Juste avant le traitement, un lavement antiseptique rectal est réalisé au sérum physiologique bétadiné, puis, après désinfection périnéale, la sonde d'échographie endorectale est introduite sous contrôle visuel et échographique.

Modalités de guidage

7.1.1.3 Guide porte-instrument

Comme pour une biopsie prostatique écho-guidée par voie transrectale, un guide à ponction est fixé sur la sonde d'échographie, afin de guider l'applicateur à micro-onde dans l'axe de la sonde (annexe).

7.1.1.4 Repérage, localisation et délimitation de la zone cible

Le système Trinity, décrit en annexe (annexe), permet une fusion d'image IRM-échographie. La tumeur détectée et localisée en IRM multiparamétrique de la prostate avant le traitement, dans le cadre des soins courants, peut ainsi être contourée et ciblée pendant le traitement.

La fusion d'image IRM-échographie est réalisée de la manière suivante :

En début d'examen, une acquisition du volume échographique prostatique est effectuée avec une sonde 3D motorisée et enregistrée dans le système Trinity. Le volume IRM 3D, préalablement chargé dans la station Trinity, est alors recalé au volume échographique. Une fois les images recalées, on place une ou plusieurs région(s) d'intérêt pour couvrir, dans les trois plans de l'espace, la surface de la tumeur localisée par l'IRM et préalablement confirmée par des biopsies guidées. Le recalage des volumes permet d'afficher les contours de la tumeur indifféremment sur le volume échographique ou IRM.

7.1.1.5 Ciblage de la région d'intérêt

Trinity est un système capable de placer avec précision, sous guidage échographique, une aiguille dans une cible localisée par IRM et recalée dans le volume échographique. Les technologies utilisées par ce système sont éprouvées pour les biopsies de la prostate, et seront exploitées ici pour le placement d'un applicateur micro-ondes. La visée s'effectue tout d'abord sous contrôle échographique 2D temps-réel. Afin de contrôler la position qu'aurait l'applicateur en cas d'insertion (position virtuelle), une échographie 3D est ensuite effectuée. Elle permet de visualiser la position virtuelle de l'applicateur ainsi que la ou les cibles dans une vue 3D. Plusieurs acquisitions 3D peuvent être nécessaires afin de faire correspondre la position virtuelle de l'applicateur avec la position des cibles, et ce avant l'insertion de l'applicateur. Une fois la sonde correctement placée, sa position est figée à l'aide du bras articulé. L'applicateur peut alors être inséré dans la prostate. La profondeur de l'applicateur dans la prostate peut être contrôlée en temps réel via l'échographie 2D. Sa bonne position au centre de la cible est vérifiée avant traitement par une ultime acquisition d'un volume échographique 3D.

Si nécessaire, la procédure peut être répétée jusqu'à ce que l'applicateur soit correctement placé. Lorsque c'est le cas, la position de l'aiguille est enregistrée et peut être consultée ultérieurement. Le traitement de la zone cible peut alors être lancé.

Modalités de délivrance des micro-ondes

7.1.1.6 Générateur à micro-ondes

Le générateur micro-onde TATO est une solution pour l'ablation (la nécrose) locale de tissus. L'intérêt de l'ablation par micro-ondes est que la propagation du rayonnement donc le volume de la zone traitée est peu sensible au type de tissu traité, ce qui permet un plus grand contrôle de l'ablation.

7.1.1.7 Applicateur à micro-ondes

Les applicateurs seront réceptionnés par la pharmacie de l'Hôpital Cochin et remis au chirurgien contre ordonnance.

7.1.1.8 **Dose d'énergie et durée du traitement**

Des mesures ont été réalisées avec le fabricant du système micro-ondes pour la réalisation d'un abaque permettant de prédire les dimensions du volume traité en fonction des paramètres de traitement (la puissance P, et le temps T). De forme elliptique, le volume traité est représenté par son diamètre D et sa longueur L.

Une loi logarithmique relie D à l'énergie émise (E = PxT), tandis que L est une relation linéaire de D :

$$D = \frac{1}{\alpha}\log\frac{E}{E_s}$$
$$L = aD + b$$

Où α est un paramètre lié à l'atténuation du rayonnement micro-ondes, E_s est l'énergie seuil à partir de laquelle le traitement est effectif, a et b sont les coefficients linéaires augmentant D en L.

Ci-après une illustration de l'abaque micro-ondes obtenue par l'expérience et l'analyse statistique, pour une puissance fixée à 10W, et pour 3 durées de traitement : 2, 4, 8 minutes, ces valeurs étant compatibles avec l'objet de l'étude. Pour ces paramètres le petit axe de la zone de traitement croît de 6mm à 14 mm, le grand axe de 13mm à 24mm (Figure 1). La Figure 2 illustre une représentation continue du petit axe traité D en fonction de la puissance et du temps.

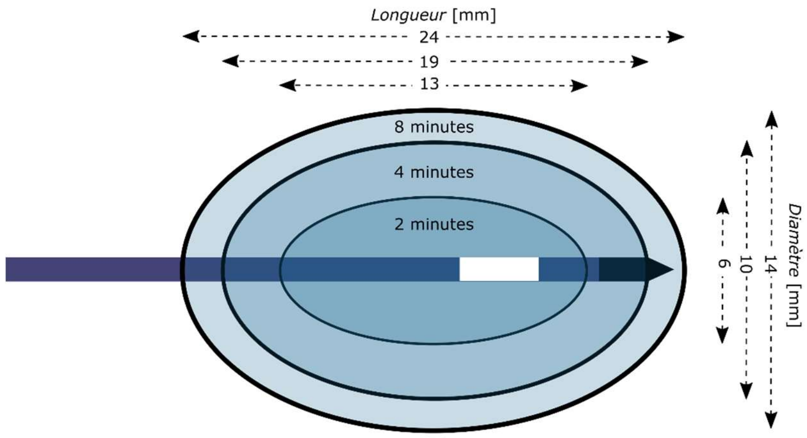

Figure 1Diametre et Longueur de la zone traitée par micro-ondes pour une puissance de 10W, pendant 2, 4, 8 minutes.

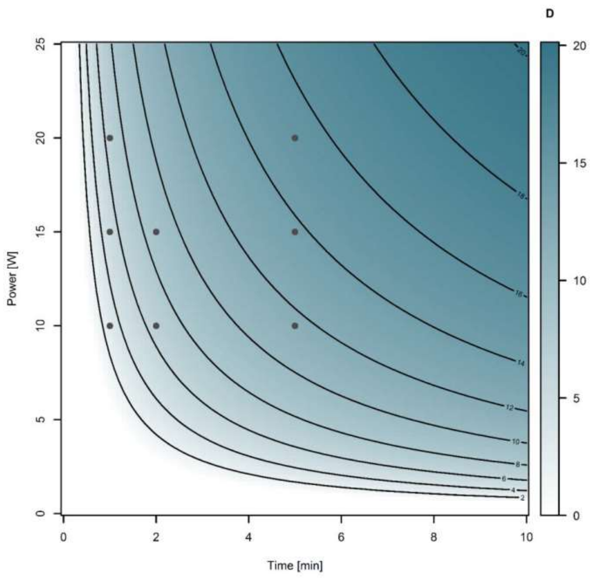

Figure 2Illustration de l'abaque micro-ondes :Petit axe D de la zone traitee fonction du temps et de la puissance

Fin de l'intervention

Immédiatement après la fin du traitement micro-onde, la sonde endo-rectale est retirée, le patient installé en décubitus dorsal strict, puis réveillé selon les mêmes modalités que les soins courants.

Soins postopératoires

Les soins postopératoires sont équivalents à ceux d'une biopsie de prostate comme elle est peut-être réalisée sous anesthésie générale, dans le cadre des soins courants.

> **En postopératoire immédiat** : Le patient est conduit en unité de surveillance post-interventionnelle.

* Il y reste pendant la durée nécessaire à la surveillance.
*Il est ensuite reconduit par l'aide-soignant de SSPI dans sa chambre.
*La continuité de la surveillance et des soins sont assurés par l'infirmière et l'aide-soignant de la salle d'hospitalisation.
*Une collation est proposée au patient deux heures après l'intervention.
*La reprise spontanée des mictions est surveillée, et la douleur évaluée par échelle visuelle analogique.

> **Le lendemain de l'intervention,** le patient est autorisé à regagner son domicile si son état de santé le permet, avec :

* L'autorisation de sortie signée par le médecin
* Le numéro de téléphone du service en cas de besoin
* Les ordonnances de sortie, comprenant la poursuite de l'antibioprophylaxie pendant 24 heures et des antalgiques de palier 1 (paracétamol) en cas de besoin.
* Le rendez-vous d'IRM prostatique de contrôle à J7 et le rendez-vous de consultation pour la visite V3.

7.2 Description des traitements non expérimentaux

Antibioprophylaxie

Une antibioprophylaxie par Ofloxacine, 200 mg matin et soir pendant 48 heures ou, en cas d'allergie à l'Ofloxacine, par Trimétoprime-Sulfamétoxazole (800 mg / 160 mg) matin et soir pendant 48 heures, sera débutée 2 heures avant l'intervention.

Lavement rectal

Un lavement rectal par Microlax sera réalisé la veille de l'intervention, puis le jour même de l'intervention 2 à 4 heures avant.

Anesthésie générale

L'anesthésie générale sera réalisée avec du propofol, associé à une faible dose de sufentanil comme analgésique. La ventilation sera assurée au moyen d'un masque laryngé et l'anesthésie entretenue avec soit du propofol en AIVOC (anesthésie intraveineuse à objectif de concentration), soit par inhalation avec du sévoflurane.

Traitements de soutien

Les traitements de soutien prévus en cours d'hospitalisation comprennent des antalgiques de palier 1 ou 2 ou des antiémétiques en cas de besoin, en intra-veineux ou per os :

- Paracétamol 500 mg per os ou Perfalgan 1 g IV
- Nefopam (Acupan) 20 mg/2 mL
- Ondansetron 8 mg IV

7.3 Description des éléments de traçabilité qui accompagnent le ou les médicaments expérimentaux

Le traitement par énergie micro-onde est réalisé en peropératoire. Le suivi de la durée cumulée de traitement et de la quantité cumulée d'énergie délivrée sont suivis en temps réel au cours de l'intervention grâce au moniteur de traitement du générateur. Ces deux paramètres sont notés dans le cahier d'observation.

Les références de l'applicateur micro-onde (à usage unique) utilisé pour le traitement sont notées dans le cahier d'observation

7.4 Traitements (médicamenteux, non médicamenteux, chirurgicaux) autorisés et interdits, y compris les médicaments de secours

Aucun traitement médical, non médical ou chirurgical n'est interdit pendant la durée de la recherche.

7.5 Méthodes de suivi de l'observance au traitement

Aucune méthode de suivi de l'observance n'est nécessaire dans le cadre de ce protocole de recherche.

8 EVALUATION DE L'EFFICACITE

8.1 Description des paramètres d'évaluation

Critère principal

Le critère d'évaluation principal est le degré de nécrose de la tumeur index, évaluée en IRM multiparamétrique de la prostate réalisée à J7 (+2 jours si nécessaire) après le traitement, dans le service de Radiologie A de l'hôpital Cochin.

La nécrose complète est définie par l'absence de rehaussement à l'IRM dynamique de contraste dans la zone cible délimitée juste avant le traitement sur l'IRM multiparamétrique.

Critères secondaires

1/ Les marges minimales et maximales de nécrose en dehors du volume cible délimité sur l'IRM préopératoire seront mesurées en comparant l'IRM multiparamétrique de la prostate réalisée 7 jours après le traitement avec l'IRM multiparamétrique de la prostate réalisée avant le traitement.

2/ La tolérance sexuelle sera évaluée par les auto-questionnaires IIEF (fonction érectile) et MSHQ-Ej (fonction éjaculatoire) (fournis en annexe). Ces auto-questionnaires seront remplis par le patient au cours des visites de suivi à J7, 2 et 6 mois après l'intervention.

3/ La tolérance urinaire sera évaluée par les auto-questionnaires IPSS et IPSS-QDV (fournis en annexe), l'examen cytobactériologique des urines, le débit urinaire maximal, le Prostate Specific Antigen (PSA) total sérique, et l'introduction de tout traitement additionnel à visée urinaire, à J7, 2 et 6 mois après l'intervention.

4/ L'évolution carcinologique sera évaluée par une IRM multiparamétrique de la prostate et des biopsies ciblées au niveau du volume traité, 6 mois après l'intervention.

8.2 Méthodes et calendrier prévus pour mesurer, recueillir et analyser les paramètres d'évaluation de l'efficacité

L'ensemble des critères d'évaluation sera rapporté dans le cahier d'observation de la recherche.

L'évaluation du critère principal sera réalisée par le Dr Alexandre Schull, radiologue et investigateur de la recherche, par étude comparative des IRM de la prostate préopératoire et postopératoire à J7 du traitement.

L'évaluation de la précision du traitement sera réalisée par le Dr Alexandre Schull, en même temps que le critère principal.

L'évaluation de la tolérance sexuelle et urinaire sera réalisée sous la direction du Dr Nicolas Barry Delongchamps. Les auto-questionnaires seront remplis directement par le patient. Les prélèvements urinaires et plasmatiques seront réalisés au sein de la consultation d'urologie de l'hôpital Cochin, et analysés au laboratoire central de l'hôpital Cochin (examen cytobactériologique des urines et dosage du PSA total sérique). La mesure du débit urinaire maximal sera réalisée au sein de la consultation du service d'urologie de l'hôpital Cochin.

L'IRM multiparamétrique réalisée 6 mois après l'intervention sera programmée au sein du même service que celle réalisée à la sélection et à 2 mois, et analysée par le Dr Alexandre Schull.

Les biopsies de la prostate de contrôle, réalisées 6 mois après l'intervention, seront réalisées au sein du service de radiologie de l'hôpital Cochin, sous la direction du Dr Alexandre Schull, et seront analysées en anatomopathologie en aveugle du résultat IRM.

L'ensemble des IRM de chaque patient sera anonymisé puis analysé en aveugle par un deuxième radiologue, le Dr Jean-Paul Abecassis. Pour chaque patient, les examens IRM seront analysés dans un ordre aléatoire de la chronologie des examens. La concordance de la mesure de l'évolution du volume de nécrose par les deux radiologues sera étudiée.

9 COMITÉS SPÉCIFIQUES DE LA RECHERCHE

9.1 Comité scientifique

Aucun comité scientifique ne sera constitué dans le cadre de ce protocole.

9.2 Comité de pilotage

Les membres du comité de pilotage sont les personnes suivantes :

- Dr Nicolas BARRY-DELONGCHAMPS, service d'urologie à Cochin
- Dr Alexandre SCHULL, service de radiologie à Cochin
- Dr Hendy ABDOUL, Unité de recherche clinique à Cochin
- Karine GOUDE-ORY, Délégation à la Recherche Clinique et à l'Innovation (DRCI) Hôpital Saint-Louis

Rôle :

- définir l'organisation générale de la recherche, coordonner les informations, déterminer initialement la méthodologie et surveiller le déroulement de la recherche.
- proposer des conduites à tenir en cours de recherche, en prenant acte des recommandations du comité de surveillance indépendant. Le promoteur DRCI reste décisionnaire.

Il se réunira selon les besoins de l'étude.

9.3 Comité de Validation des Evénements Critiques

Cette étude ne faisant courir aucun risque particulier aux sujets inclus, le comité ne sera pas constitué.

10 EVALUATION DE LA SECURITÉ – RISQUES ET CONTRAINTES AJOUTES PAR LA RECHERCHE

10.1 Description des paramètres d'évaluation de la sécurité

Les constantes cardio-respiratoires en peropératoire et en postopératoire immédiat pendant les 2 premières heures suivant le réveil du patient.

L'extension anatomique de la nécrose intra-prostatique provoquée par le traitement à l'étude, en particulier l'aspect radiologique des structures anatomiques adjacentes.

La tolérance sexuelle du traitement, en particulier sur le plan érectile et éjaculatoire.

La tolérance urinaire du traitement, sur la plan du risque infectieux et sur le plan fonctionnel.

10.2 Méthodes et calendrier prévus pour mesurer, recueillir et analyser les paramètres d'évaluation de la sécurité

Unité de surveillance post-interventionelle :

Tous les patients traités seront surveillés en peropératoire selon les recommandations de bonnes pratiques de la société française d'anesthésie et de réanimation. En postopératoire immédiat, les patients seront surveillés en unité de surveillance post-interventionnelle pendant deux heures, avec une évaluation continue de leurs constantes cardio-respiratoires.

Ce type de surveillance n'est pas une contrainte ajoutée par la recherche.

IRM multiparamétrique de la prostate :

L'IRM permettra d'évaluer l'étendue de la nécrose intra-prostatique postopératoire et de s'assurer de l'absence d'atteinte des structures anatomiques péri-prostatiques. Elle sera réalisée 7 jours après l'intervention.

Evaluation de la tolérance sexuelle :

La tolérance sexuelle sera évaluée par les auto-questionnaires IIEF (fonction érectile) et MSHQ-Ej (fonction éjaculatoire) (fournis en annexe). Ces auto-questionnaires seront remplis par le patient au cours des visites de suivi à J7, 2 et 6 mois après l'intervention.

Evaluation de la tolérance urinaire :

La tolérance urinaire sera évaluée avant la sortie d'hospitalisation par la surveillance de la reprise mictionnelle spontanée postopératoire.

Un examen cytobactériologique des urines permettra d'éliminer toute bactériurie postopératoire. Il sera réalisé pour la visite au 7ème jour postopératoire.

Les symptômes urinaires seront ensuite évalués par les auto-questionnaires IPSS et IPSS-QDV (fournis en annexe) et la mesure du débit urinaire au cours des visites de suivi à J7, 2 et 6 mois après l'intervention.

10.3 Définitions

D'après l'article R1123-46 du Code de la Santé Publique :

- **Evènement indésirable (EI)**

Toute manifestation nocive survenant chez une personne qui se prête à une recherche impliquant la personne humaine que cette manifestation soit liée ou non à la recherche ou au produit sur lequel porte cette recherche.

- **Effet indésirable d'un dispositif médical (DM) ou d'un dispositif médical de diagnostic *in vitro* (DMDIV)**

Toute réaction nocive et non désirée à un dispositif médical ou tout incident qui aurait pu entraîner cette réaction si une action appropriée n'avait pas été effectuée, chez une personne qui se prête à la recherche ou chez l'utilisateur du dispositif médical ou tout effet lié

à une défaillance ou une altération d'un dispositif médical de diagnostic *in vitro* et néfaste pour la santé d'une personne qui se prête à la recherche.

- **Evènement ou effet indésirable grave**

Tout évènement ou effet indésirable qui entraîne la mort, met en danger la vie de la personne qui se prête à la recherche, nécessite une hospitalisation ou la prolongation de l'hospitalisation, provoque une incapacité ou un handicap importants ou durables, ou bien se traduit par une anomalie ou une malformation congénitale, et s'agissant du médicament, quelle que soit la dose administrée.

- **Effet indésirable inattendu**

Tout effet indésirable dont la nature, la sévérité ou l'évolution ne concorde pas avec les informations relatives aux produits, actes, pratiqués et méthodes utilisées au cours de la recherche.

D'après l'article R5212-15 du Code de la Santé Publique :

- **Incident**
  - Réaction nocive et non voulue se produisant lors de l'utilisation d'un dispositif médical conformément à sa destination ;
  - Réaction nocive et non voulue résultant d'une utilisation d'un dispositif médical ne respectant pas les instructions du fabricant ;
  - Tout dysfonctionnement ou toute altération des caractéristiques ou des performances d'un dispositif médical ;
  - Toute indication erronée, omission et insuffisance dans la notice d'instruction, le mode d'emploi ou le manuel de maintenance.

D'après l'article R.1123-46 du Code de la Santé Publique et l'avis aux promoteurs d'essais cliniques portant sur des dispositifs médicaux et dispositifs médicaux de diagnostic *in vitro* (ANSM) :

- **Fait nouveau**

Toute nouvelle donnée pouvant conduire à une réévaluation du rapport des bénéfices et des risques de la recherche ou du produit objet de la recherche, à des modifications dans l'utilisation de ce produit, dans la conduite de la recherche, ou des documents relatifs à la recherche, ou à suspendre ou interrompre ou modifier le protocole de la recherche ou des recherches similaires. Pour les essais portant sur la première administration ou utilisation d'un produit de santé chez des personnes qui ne présentent aucune affection : tout effet indésirable grave.

Exemples :
a) toute augmentation cliniquement significative de la fréquence d'apparition d'un effet indésirable grave attendu ;
b) des suspicions d'effets indésirables graves inattendus survenus chez des participants ayant terminé l'essai et qui sont notifiés par l'investigateur au promoteur, ainsi que des rapports de suivi éventuels ;
c) tout fait nouveau concernant le déroulement de l'essai clinique ou l'utilisation du dispositif médical, lorsque ce fait nouveau est susceptible de porter atteinte à la sécurité des participants.
A titre d'exemples :

- un évènement indésirable grave susceptible d'être lié aux investigations et aux procédures de diagnostic de l'essai et qui pourrait modifier le déroulement de cet essai,

- un risque significatif pour la population de l'essai comme par exemple un manque d'efficacité du dispositif médical utilisé dans le traitement d'une maladie mettant en jeu le pronostic vital,
- des résultats significatifs issus d'une étude préclinique terminée pouvant remettre en question l'évaluation des risques par rapport au bénéfice attendu (telle qu'une étude de biomécanique),
- un arrêt anticipé ou une interruption temporaire pour des raisons de sécurité d'un essai conduit avec le même dispositif médical dans un autre pays,
- un évènement indésirable grave inattendu lié à un produit de santé non expérimental nécessaire à la réalisation de l'essai (ex : « challenge agents », traitement de secours)

d) les recommandations du comité de surveillance indépendant (CSI), le cas échéant, si elles sont pertinentes pour la sécurité des personnes,
e) tout évènement indésirable grave inattendu transmis au promoteur par un autre promoteur d'un essai clinique portant sur le même dispositif médical dans un pays tiers.

10.4 Rôles de l'investigateur

L'investigateur doit **évaluer pour chaque évènement indésirable sa gravité** et reporter tous les évènements indésirables graves et non graves dans le cahier d'observation (CRF).

L'investigateur doit **documenter au mieux** les évènements indésirables graves et donner dans la mesure du possible, le diagnostic médical définitif.

L'investigateur doit **évaluer l'intensité** des évènements indésirables par des termes généralistes :
❖ Léger : toléré par le patient, n'interférant pas avec ses activités quotidiennes
❖ Modéré : suffisamment inconfortable pour altérer les activités quotidiennes
❖ Sévère : qui empêche les activités quotidiennes

De même, chaque évènement indésirable sera classé selon la classification de Clavien Dindo, communément utilisée dans l'évaluation des complications chirurgicales.

L'investigateur doit **évaluer le lien de causalité** des évènements indésirables graves avec le dispositif médical expérimental, et/ou son geste de mise en œuvre et/ou les actes/procédures ajoutées par la recherche et avec les autres traitements éventuels.
La méthode utilisée par l'investigateur, basée sur la méthode OMS (WHO Uppsala Monitoring Centre), repose sur les 4 termes de causalité suivants :

- Certain
- Probable/plausible
- Possible
- Improbable

Leur définition est présentée dans le tableau suivant (extrait de WHO-UMC causality categories, version du 17/04/2012).

TABLEAU : WHO-UMC causality categories (extrait)

**Causality term**	Assessment criteria*
**Certain**	· Event or laboratory test abnormality, with plausible time relationship to drug intake **<br>· Cannot be explained by disease or other drugs<br>· Response to withdrawal plausible (pharmacologically, pathologically)<br>· Event definitive pharmacologically or phenomenologically (i.e. an objective and specific medical disorder or a recognized pharmacological phenomenon)<br>· Rechallenge satisfactory, if necessary

**Probable / Likely**	· Event or laboratory test abnormality, with reasonable time relationship to drug intake<br>· Unlikely to be attributed to disease or other drugs<br>· Response to withdrawal clinically reasonable<br>· Rechallenge not required
**Possible**	· Event or laboratory test abnormality, with reasonable time relationship to drug intake<br>· Could also be explained by disease or other drugs<br>· Information on drug withdrawal may be lacking or unclear
**Unlikely**	· Event or laboratory test abnormality, with a time to drug intake<br>· that makes a relationship improbable (but not impossible)<br>· Disease or other drugs provide plausible explanations

*All points should be reasonably complied with
** Or study procedures

L'investigateur précisera si l'évènement indésirable grave fait suite à un incident lié au dispositif médical expérimental et/ou à son geste de mise en œuvre.

L'investigateur ayant connaissance d'un incident doit le signaler au promoteur sans délai dès la prise de connaissance. Il remplit la fiche de signalement d'un incident ou risque d'incident téléchargeable sur le site de l'ANSM et l'envoie par télécopie sans délai dès la prise de connaissance au secteur Vigilance de la DRCI (AP-HP) au 01 44 84 17 99.

10.4.1 Evènements indésirables graves nécessitant une notification sans délai par l'investigateur au promoteur

Conformément à l'article R.1223-49, l'investigateur notifie au promoteur sans délai à compter du jour où il en a connaissance tous les événements indésirables graves, survenus au cours d'une recherche mentionnée au 1° de l'article L.1121-1, à l'exception de ceux qui sont recensés dans le protocole (cf. section 10.4.2.3) et, le cas échéant, dans la brochure pour l'investigateur comme ne nécessitant pas une notification sans délai (extrait de l'article R.1123-49 du Code de la Santé Publique).
L'investigateur notifie ces événements dans un délai approprié en tenant compte des spécificités de la recherche et de l'événement indésirable grave ainsi que d'éventuelles indications figurant dans le protocole ou la brochure pour l'investigateur. Cette notification fait l'objet d'un rapport écrit et est suivie de rapports complémentaires écrits détaillés.

Un évènement indésirable grave présente l'un des critères suivants :

**1-**	évènement qui entraîne la mort,
**2-**	évènement qui met en danger la vie de la personne qui se prête à la recherche,
**3-**	évènement qui nécessite une hospitalisation ou la prolongation de l'hospitalisation,
**4-**	évènement qui provoque une incapacité ou un handicap importants ou durables,
**5-**	évènement qui se traduit par une anomalie ou une malformation congénitale.

10.4.2 Spécificités du protocole

10.4.2.1 Autres évènements nécessitant une notification sans délai par l'investigateur au promoteur

- Evénements indésirables post-opératoires de grade ≥ 3b selon la classification de Clavien-Dindo
- Tout EIG faisant suite à un incident dans les modalités de délivrance des micro-ondes (ex : dose d'énergie et durée de traitement non conforme à l'abaque)
- Cancers secondaires

Ces évènements indésirables doivent être notifiés au promoteur par l'investigateur sans délai à compter du jour où il en a connaissance, selon les mêmes modalités et délais que les évènements indésirables graves (cf. section 10.4.4).

10.4.2.2 **Evènements indésirables graves ne nécessitant pas une notification sans délai par l'investigateur au promoteur**

Ces évènements indésirables graves sont uniquement recueillis dans la partie « évènement indésirable » du cahier d'observation.

- Evolution naturelle et habituelle de la pathologie :
    - hospitalisation programmée pour le suivi de la pathologie étudiée,
    - hospitalisation pour traitement de routine ou surveillance de la pathologie étudiée non associée à une détérioration de l'état du sujet,
    - aggravation de la pathologie étudiée : évolution carcinologique nécessitant un traitement radical, tel que proposé dans le cadre des soins courants

- Circonstances particulières
    - prolongation d'hospitalisation en unité de surveillance post-interventionnelle au-delà du délai de 3 heures prévu par le protocole
    - complications post-opératoire de grade < 3b selon la classification de Clavien-Dindo
    - hospitalisation pour une pathologie préexistante
    - hospitalisation pour traitement médical ou chirurgical programmé avant la recherche
    - admission pour raisons sociale ou administrative
    - passage aux urgences (<12 heures)

- Evènements indésirables susceptibles d'être liés aux traitements/actes prescrits dans le cadre du soin pendant le suivi de la recherche
  L'investigateur, comme tout professionnel de santé, doit notifier ces évènements aux vigilances sanitaires applicables. Exemples : Agence Régionale de Santé, direction de la qualité de l'établissement, Centre Régional de Pharmacovigilance, correspondant local de matériovigilance (ANSM), etc.

    - Evènements indésirables survenus à distance du geste de mise en œuvre du dispositif médical expérimental. Le délai à partir duquel le lien de causalité entre la survenue de l'EIG et le geste de mise en œuvre du dispositif médical expérimental peut être exclu est de 30 jours. Ce délai est communément admis après une intervention chirurgicale.

10.4.3 Période de notification sans délai des EIG par l'investigateur au promoteur

L'investigateur doit notifier sans délai au promoteur les évènements indésirables graves tels que définis dans la rubrique correspondante :

❖ à partir de la date de l'intervention (date d'application des micro-ondes),

❖ pendant toute la durée de suivi du participant prévue par la recherche,

❖ sans limitation de durée, lorsque l'EIG est susceptible d'être dû au dispositif médical et/ou aux actes/procédures/examens spécifiques à la recherche.

10.4.4 Modalités et délais de notification au promoteur

La notification initiale d'EIG fait l'objet d'un rapport écrit et signé par l'investigateur à l'aide d'un formulaire spécifique de notification des EIG prévu à cet effet dans le cahier d'observation.

Chaque item de ce document doit être complété par l'investigateur pour permettre au promoteur d'effectuer une analyse pertinente.

La notification initiale d'un événement indésirable grave au promoteur doit être suivie rapidement par un (ou des) rapport(s) complémentaire(s) détaillé(s), écrit(s) permettant de suivre l'évolution du cas en vigilance ou de compléter les informations.

L'investigateur transmettra, dans la mesure du possible, tout document pouvant être utile au promoteur (comptes-rendus médicaux, résultats biologiques, résultats d'examens complémentaires, etc). Ces documents devront être rendus anonymes. Par ailleurs, ils devront être complétés par les mentions suivantes : acronyme de la recherche, numéro et initiales du participant.

Tout évènement indésirable sera suivi jusqu'à sa complète résolution (stabilisation à un niveau jugé acceptable par l'investigateur ou retour à l'état antérieur) même si le participant est sorti de la recherche.

La notification initiale, les rapports de suivi d'EIG et tout autre document seront transmis au promoteur représenté par son secteur Vigilance exclusivement par télécopie au **01 44 84 17 99**.

Dans le cas d'une recherche avec e-CRF :
- l'investigateur complète le formulaire de notification d'EIG dans l'e-CRF, le valide, l'imprime, le signe puis l'envoi par télécopie.
- en cas d'impossibilité de connexion à l'e-CRF, l'investigateur complètera, signera et adressera le formulaire de notification d'EIG inséré en annexe 2. Dès que la connexion sera rétablie, il régularisera en complétant le formulaire de notification d'EIG de l'e-CRF.

L'investigateur doit répondre à toute demande d'information complémentaire émanant du promoteur.
Pour toute question relative à la notification d'un évènement indésirable, il est possible de contacter le secteur Vigilance par courriel : vigilance.drc@aphp.fr

10.5 Rôles du promoteur

Le promoteur représenté par son secteur Vigilance évalue la sécurité de chaque dispositif médical de façon continue, tout au long de la recherche.

10.5.1 Analyse et déclaration des évènements indésirables graves

Le promoteur évalue :
- la **gravité** de tous les évènements indésirables qui lui sont rapportés,
- leur **lien de causalité** avec chaque dispositif médical expérimental et/ou son geste de mise en œuvre et/ou les actes/procédures/examens spécifiques ajoutés par la

recherche et avec les autres traitements éventuels. Tous les évènements indésirables graves pour lesquels l'investigateur et/ou le promoteur estiment qu'une relation de causalité avec le dispositif médical expérimental peut être raisonnablement envisagée sont considérés comme des suspicions d'effets indésirables graves.

- le **caractère attendu ou inattendu** des effets indésirables.
  Tout effet indésirable grave dont la nature, la sévérité ou l'évolution ne concordent pas avec les informations figurant dans la notice d'utilisation lorsque le dispositif médical fait l'objet d'un marquage CE ou dans la brochure pour l'investigateur lorsque le produit n'est pas autorisé, est considéré comme inattendu.
  L'évaluation du caractère attendu/inattendu d'un effet indésirable grave est effectuée par le promoteur représenté par son secteur Vigilance sur la base des informations décrites ci-dessous (cf. section 10.5.1.1).

Information de sécurité de référence

❖ Pour les évènements indésirables graves susceptibles d'être liés au dispositif médical expérimental et/ou à son geste de mise en œuvre et attendus, il convient de se référer à la notice d'utilisation «TATO® de Biomedical ».

Les évènements indésirables graves attendus sont liés à l'effet thermique des micro-ondes, entrainant une nécrose circonscrite autour de l'applicateur. L'extension de la zone de nécrose peut être liée soit à une imprécision du guidage, soit à une diffusion trop large de l'effet thermique des micro-ondes.

❖ Une extension de la zone de nécrose au-delàs de la zone cible peut entrainer, dans un délai allant de quelques heures à environ 30 jours :

- une nécrose de la paroi rectale pouvant entrainer :
  - une fistule recto-prostatique ou recto-urétrale,
  - une prostatite aigue,
  - une rectorragie légère, modérée, ou massive avec syndrome hémorragique aigu,
- une dysfonction érectile liée à l'atteinte des bandelettes vasculo-nerveuses,
- des troubles mictionnels liés à une atteinte urétrale, et se caractérisant par des urgences mictionnelles, une pollakiurie, et/ou des brulures mictionnelles,
- une hématurie légère à sévère, avec risque de syndrome hémorragique aigu.

Le risque d'extension de la zone de nécrose sera néanmoins évalué précisément sur l'IRM postopératoire réalisée à J7.

❖ Les événements indésirables liés aux actes/procédures/examens spécifiques à la recherche :

  o Préparation à l'intervention : se référer aux RCP Oflocet®, Bactrim® et Microlax®/Normacol®

  o Immobilisation : complications thrombo-emboliques post-opératoires

  o Anesthésie générale :
    - pneumopathie postopératoire liée à l'intubation trachéale,
    - choc anaphylactique secondaire aux drogues anesthésiques : se référer aux RCP de Propofol® ou Sevorane®

  o Traitement de soutien : se référer aux RCP d'Acupan®, du paracetamol et de l'ondansetron administré.

- IRM : allergie ou intolérance au Gadolinium injecté par voie intra-veineuse (se référer au RCP Gadolinium), claustrophobie

Le promoteur déclare :

- toute suspicion d'effet indésirable grave inattendu dû à un dispositif médical expérimental faisant l'objet de la recherche (SUSAR)
- tous les événements indésirables graves susceptibles d'être liés au geste de mise en œuvre du dispositif médical expérimental faisant l'objet de la recherche.
- tout incident ayant conduit à la survenue d'un évènement indésirable grave.

NB : le promoteur déclare tout incident ayant conduit à la survenue d'un évènement indésirable grave auprès de l'ANSM (boîte mail dédiée à la transmission des données relatives aux essais cliniques des DM, DM DIV et cellule de matériovigilance de l'ANSM).

Le promoteur déclare toute suspicion d'effet indésirable grave inattendu (SUSAR) et tout événement indésirable grave pouvant être lié au geste de mise en œuvre du dispositif médical survenus en France et en dehors du territoire national concerné, dans les délais réglementaires, auprès de l'Agence nationale de sécurité des médicaments et des produits de santé (ANSM) :

- La déclaration initiale doit être réalisée sans délai à compter du jour où le promoteur en a eu connaissance dans le cas d'effet indésirable grave inattendu et d'événement indésirable grave pouvant être lié au geste de mise en œuvre du dispositif médical ayant entraîné la mort ou mis la vie en danger et dans un délai de 15 jours à compter du jour où le promoteur en a eu connaissance pour le cas des autres effets indésirables graves inattendus et événement indésirable grave pouvant être lié au geste de mise en œuvre du dispositif médical ;

- Dans le cas d'effet indésirable grave inattendu ou d'événement indésirable grave pouvant être lié au geste de mise en œuvre du dispositif médical ayant entraîné la mort ou mis la vie en danger, les informations complémentaires pertinentes sont notifiées à l'Agence nationale de sécurité du médicament et des produits de santé dans un délai de huit jours.

- Dans les autres cas d'effets indésirables graves inattendus et d'événements indésirables graves pouvant être liés au geste de mise en œuvre du dispositif médical, les informations complémentaires pertinentes sont transmises à l'Agence nationale de sécurité du médicament et des produits de santé dans un délai de quinze jours.

Le promoteur informe tous les investigateurs concernés de toute donnée qui pourrait avoir un impact défavorable sur la sécurité des personnes qui se prêtent à la recherche.

10.5.2 Analyse et déclaration des autres données de sécurité

D'après l'article R.1123-46 du Code de la Santé Publique et l'avis aux promoteurs d'essais cliniques portant sur des dispositifs médicaux et dispositifs médicaux de diagnostic *in vitro* (ANSM), un fait nouveau est défini par toute nouvelle donnée pouvant conduire à une réévaluation du rapport des bénéfices et des risques de la recherche ou du produit objet de la recherche, à des modifications dans l'utilisation de ce produit, dans la conduite de la recherche, ou des documents relatifs à la recherche, ou à suspendre ou interrompre ou modifier le protocole de la recherche ou des recherches similaires. Pour les essais portant

sur la première administration ou utilisation d'un produit de santé chez des personnes qui ne présentent aucune affection : tout effet indésirable grave.

Le promoteur informe sans délai à compter du jour où il en a connaissance l'autorité compétente et le comité de protection des personnes des faits nouveaux et, le cas échéant, des mesures urgentes de sécurité prises.

A la suite de la déclaration initiale relative à un fait nouveau, le promoteur adresse aux autorités compétentes sous forme d'un rapport de suivi du fait nouveau, toute information complémentaire pertinente relative à ce fait nouveau dans un délai de 15 jours maximum à compter du moment où il dispose de ces informations.

Si une suspicion d'effet indésirable grave inattendu (SUSAR) ou un évènement indésirable grave pouvant être lié au geste de mise en œuvre du DM répond à la définition d'un fait nouveau, l'évènement correspondant doit faire l'objet d'une double déclaration de la part du promoteur, selon les modalités et les délais précédemment cités.

10.5.3 Rapport annuel de sécurité

Le promoteur doit établir une fois par an pendant toute la durée de l'essai clinique un rapport annuel de sécurité (RAS ou annual safety report – [ASR]) comprenant notamment :
- une analyse de la sécurité des personnes qui se prêtent à la recherche,
- une liste de toutes les suspicions d'effets indésirables graves et une liste des évènements indésirables graves pouvant être liés au geste de mise en œuvre du dispositif médical, survenus pendant la période couverte par le rapport,
- les tableaux de synthèse de tous les effets indésirables graves survenus depuis le début de la recherche.

Le rapport est transmis à l'ANSM et au CPP dans les 60 jours après la date anniversaire correspondant à la date du premier patient inclus dans la recherche.

10.6 Comité de Surveillance Indépendant

Un Comité de Surveillance Indépendant (CSI) est prévu dans le cadre de cette recherche. Il a comme principale mission d'être un comité de suivi des données de sécurité. Une réunion préliminaire du CSI est prévue avant la première inclusion du premier sujet.
Les membres du CSI sont :

- Urologue (à prévoir)
- Dr. Cédric LAOUENAN, département d'épidémiologie biostatistique et recherche clinique, Hôpital Bichat, Paris
- Dr. Caroline ESCOURROU, radiologie, médecin libéral, Paris

L'ensemble des missions ainsi que les modalités précises de fonctionnement du CSI sont décrites dans la charte du CSI de l'étude.

Le CSI a une fonction consultative, le promoteur reste décisionnaire.

11 GESTION DES DONNEES

11.1 Modalités de recueil des données

Toutes les informations requises par le protocole doivent être fournies dans le cahier d'observation et une explication donnée par l'investigateur pour chaque donnée manquante.
Les données devront être reportées dans les cahiers d'observation électroniques au fur et à mesure qu'elles sont obtenues qu'il s'agisse de données cliniques ou para-cliniques.
Les données erronées dépistées sur les cahiers d'observation feront l'objet d'une demande de correction. Avec le cahier d'observation électronique, les modifications seront tracées par l'outil informatique.

11.2 Identification des données recueillies directement dans les CRF qui seront considérées comme données-source

Il s'agira principalement des données suivantes :
- les données relatives au patient : date de naissance (mois/année), âge et initiales nom et prénom.
- les données concernant les différents examens effectués dans le cadre de la recherche (résultats IRM multiparamétrique, toucher rectal, bilan biologique)
- les données recueillies à partir des questionnaires patients (IIEF, MSHQ-Ej, IPSS et IPSS-QDV)
- les évènements indésirables recueillis

11.3 Droit d'accès aux données et documents source

Accès aux données

Conformément aux BPC :
- le promoteur est chargé d'obtenir l'accord de l'ensemble des parties impliquées dans la recherche afin de garantir l'accès direct à tous les lieux de déroulement de la recherche, aux données source, aux documents source et aux rapports dans un but de contrôle de qualité et d'audit par le promoteur ou d'inspection par l'autorité compétente,
- les investigateurs mettront à disposition des personnes chargées du suivi, du contrôle de qualité, de l'audit ou de l'inspection de la recherche impliquant la personne humaine, les documents et données individuelles strictement nécessaires à ce contrôle, conformément aux dispositions législatives et réglementaires en vigueur (articles L.1121-3 et R.5121-13 du code de la santé publique).

Documents source

Les documents source étant définis comme tout document ou objet original permettant de prouver l'existence ou l'exactitude d'une donnée ou d'un fait enregistrés au cours de la recherche seront conservés selon la réglementation en vigueur par l'investigateur ou par l'hôpital s'il s'agit d'un dossier médical hospitalier.

Les données considérées comme données sources seront le dossier médical, les comptes rendus de consultations, les résultats biologiques, les clichés radiologiques et leur compte-rendu.

Confidentialité des données

Les personnes chargées du contrôle de qualité d'une recherche impliquant la personne humaine (article L.1121-3 du code de la santé publique), prendront toutes les précautions nécessaires en vue d'assurer la confidentialité des informations relatives à la recherche, aux personnes qui s'y prêtent et notamment en ce qui concerne leur identité ainsi qu'aux résultats obtenus.

Ces personnes, au même titre que les investigateurs eux-mêmes, sont soumises au secret professionnel (selon les conditions définies par les articles 226-13 et 226-14 du code pénal).
Pendant la recherche impliquant la personne humaine ou à son issue, les données recueillies sur les personnes qui s'y prêtent et transmises au promoteur par les investigateurs (ou tous autres intervenants spécialisés) seront rendues non identifiantes.
Elles ne doivent en aucun cas faire apparaître en clair les noms des personnes concernées ni leur adresse.
Seules les initiales du nom et du prénom seront enregistrées, accompagnées d'un numéro codé propre à la recherche indiquant l'ordre d'inclusion des sujets.
Le promoteur s'assurera que chaque personne qui se prête à la recherche a donné son accord par écrit pour l'accès aux données individuelles la concernant et strictement nécessaires au contrôle de qualité de la recherche.

11.4 Traitement des données et conservation des documents et des données

Identification du responsable et du(es) lieu(x) de la gestion du(es) traitement(s) des données

La saisie des données sera réalisée par les investigateurs sur un support électronique via un navigateur internet.
L'analyse des données sera réalisée par un biostatisticien de l'URC Paris Descartes Cochin Necker.

Saisie des données

La saisie des données sera réalisée sur un support électronique via un navigateur internet.

Traitements des données (CNIL) en France

Cette recherche entre dans le cadre de la « Méthodologie de Référence pour les traitements de données personnelles opérés dans le cadre des recherches interventionnelles impliquant la personne humaine » (MR-001 modifiée). L'AP-HP, promoteur de la recherche, a signé un engagement de conformité à cette « Méthodologie de Référence ».

Archivage

Les documents spécifiques d'une recherche impliquant la personne humaine portant sur un dispositif médical ou sur un dispositif médical de diagnostic in vitro seront archivés par l'investigateur et le promoteur pour une durée de 15 ans après la fin de la recherche.

Cet archivage indexé comporte notamment :

- Une enveloppe scellée pour l'investigateur contenant un exemplaire de toutes les notes d'information et les formulaires de consentement signés de toutes les personnes du centre ayant participé à la recherche ;
- Une enveloppe scellée pour le promoteur contenant un exemplaire de toutes les notes d'information et les formulaires de consentement signés de toutes les personnes du centre ayant participé à la recherche ;
- Les classeurs « recherche » pour l'Investigateur et le promoteur comprenant :
  - les versions successives du protocole (identifiées par le n° et la date de version), ses annexes
  - les autorisations de l'ANSM et les avis du CPP
  - les courriers de correspondance,
  - la liste ou registre d'inclusion,

- les annexes spécifiques à la recherche
- le rapport final de la recherche.

- Les documents de recueil des données

11.5 Propriété des données

L'AP-HP est propriétaire des données et aucune utilisation ou transmission à un tiers ne peut être effectuée sans son accord préalable.

12 ASPECTS STATISTIQUES

12.1 Hypothèses de calcul du nombre de sujets nécessaires et résultat

Un design d'étude de phase II a été utilisé pour le calcul du nombre de sujet en considérant qu'un taux de nécrose complète de 99 % serait désirable. Si ce taux était de 60 % ou moins l'intervention serait considérée comme inefficace.
Conformément à ces hypothèses et avec un risque alpha de 0.1 et un risque beta de 0.1, un total de 5 patients devrait être nécessaire. Pour pouvoir étudier la tolérance de l'intervention un nombre supplémentaire de 5 patients sera prévu soit un total de 10 patients à inclure.

12.2 Description des méthodes statistiques prévues y compris du calendrier des analyses intermédiaires prévues

L'analyse sera réalisée à l'aide du logiciel R (http://cran.r-project.org).L'analyse statistique sera effectuée à la fin du recueil, de la saisie et du contrôle de cohérence des données. Aucune analyse intermédiaire n'est prévue dans ce protocole.

Pour l'analyse du critère d'évaluation principal, l'effectif et le pourcentage de patients ayant une nécrose complète du volume cible 7 jours après le traitement seront donnés. L'intervalle de confiance à 95% (en unilatéral) sera calculé via la méthode exacte de Clopper-Pearson.

Pour les analyses secondaires :
Les marges minimales et maximales de nécrose seront données pour chacun des 10 patients (une médiane et un intervalle interquartile sera éventuellement calculé). Les scores obtenus aux auto-questionnaires IIEF (fonction érectile), MSHQ-Ej (fonction éjaculatoire) IPSS et IPSS-QDV à J7 2 et 6 mois seront données pour chacun des 10 patients (une médiane et un intervalle interquartile sera éventuellement calculé). Le débit urinaire maximal et le Prostate Specific Antigen (PSA) total sérique seront rapportés pour chaque patient (une médiane et un intervalle interquartile sera éventuellement calculé).
Le résultat de l'examen cytobactériologique des urines et l'introduction de tout traitement additionnel à visée urinaire à J7, 2 et 6 mois après l'intervention sera précisé pour chaque patient. Un pourcentage accompagné de son intervalle de confiance à 95% sera donné pour chacune des deux variables.

13 CONTRÔLE ET ASSURANCE DE LA QUALITE

Chaque projet de recherche interventionnelle impliquant la personne humaine pris en charge par l'AP-HP est classé selon le risque prévisionnel encouru par les personnes se prêtant aux recherches grâce à la classification des recherches interventionnelle impliquant la personne humaine à promotion AP-HP.

13.1 Organisation générale

Le promoteur doit s'assurer de la sécurité et du respect des personnes qui ont accepté de participer à la recherche. Il doit mettre en place un système d'assurance qualité permettant de surveiller au mieux le déroulement de la recherche dans les centres investigateurs.

A cet effet, le promoteur mandate des Attachés de Recherche Clinique (ARC) qui ont pour mission principale d'effectuer des visites régulières de suivi dans les lieux de recherche après avoir effectué les visites d'ouvertures.
Les objectifs du suivi de la recherche, tels que définis dans les Bonnes Pratiques Cliniques, (BPC § 5.18.1) sont de vérifier que :

- le droit, la sécurité et la protection des personnes qui se prêtent à la recherche sont satisfaits,
- les données rapportées sont exactes, complètes et cohérentes avec les documents sources,
- la recherche est conduite conformément au protocole en vigueur, aux BPC et aux dispositions législatives et réglementaires en vigueur.

Stratégie d'ouverture des centres

La stratégie d'ouverture des centres mise en place pour cette recherche est déterminée grâce au plan de monitoring adapté. Avant toute inclusion, le centre recruteur sera ouvert par une visite de mise en place sur site, pour une mise en place du protocole et prise de connaissance avec les différents intervenants de la recherche impliquant la personne humaine.

Etendue du monitoring des centres

Dans le cas de cette recherche à risque **D** le choix d'un niveau de monitoring adapté a été pondéré en fonction de la complexité, l'impact et le budget de la recherche. A cet effet, le promoteur en accord avec l'investigateur coordonnateur a déterminé le score logistique et impact qui a permis d'obtenir le niveau de monitoring à mettre en place sur la recherche : niveau **élevé.**
Ces différents niveaux sont définis dans la charte du monitoring des Recherches Impliquant la personne humaine.

13.2 Contrôle de qualité

Un Attaché de Recherche Clinique (ARC) mandaté par le promoteur s'assurera de la bonne réalisation de la recherche, du recueil des données générées par écrit, de leur documentation, enregistrement et rapport, en accord avec les Procédures Opératoires Standard mises en application au sein de la DRCI et conformément aux Bonnes Pratiques Cliniques ainsi qu'aux dispositions législatives et réglementaires en vigueur.

L'investigateur et les membres de son équipe acceptent de se rendre disponibles lors des visites de Contrôle de qualité effectuées à intervalles réguliers par l'Attaché de Recherche Clinique. Lors de ces visites, les éléments suivants seront revus :

- consentement écrit ;
- respect du protocole de la recherche et des procédures qui y sont définies ;
- qualité des données recueillies dans le cahier d'observation : exactitude, données manquantes, cohérence des données avec les documents "source" (dossiers médicaux, carnets de rendez-vous, originaux des résultats de laboratoire, etc.) ;
- gestion des traitements utilisés.

13.3 Cahier d’observation

Toutes les informations requises par le protocole doivent être consignées dans les cahiers d’observation. Les données devront être recueillies au fur et à mesure qu'elles sont obtenues, et enregistrées dans ces cahiers de façon explicite. Chaque donnée manquante devra être codée.
Ce cahier d’observation électronique sera mis en place dans le centre grâce à un support Internet de recueil des données. Un document d’aide pour l’utilisation de cet outil sera fourni aux investigateurs.
Le remplissage du cahier d'observation via internet par l'investigateur permet ainsi à l'ARC de visualiser rapidement et à distance les données. L'investigateur est responsable de l'exactitude, de la qualité et de la pertinence de toutes les données saisies. De plus, lors de leurs saisies, ces données sont immédiatement vérifiées grâce à des contrôles de cohérence. A ce titre, il doit valider toute modification de valeur dans le CRF. Ces modifications font l'objet d'un audit trail. Une justification peut éventuellement être intégrée en commentaire. Une impression papier sera demandée en fin d'étude, authentifiée (datée et signée) par l'investigateur. Une copie du document authentifié à destination du promoteur devra être archivée par l’investigateur.

13.4 Gestion des non conformités

Tout évènement survenant suite au non-respect du protocole, des procédures opératoires standardisées, des bonnes pratiques cliniques ou des dispositions législatives et réglementaires en vigueur par un investigateur ou toute autre personne impliquée dans la conduite de la recherche doit faire l’objet d’une déclaration de non-conformité au promoteur.

Ces non-conformités seront gérées conformément aux procédures du promoteur.

13.5 Audit / inspections

Les investigateurs s’engagent à accepter les audits d’assurance qualité effectués par le promoteur ainsi que les inspections effectuées par les autorités compétentes. Toutes les données, tous les documents et rapports peuvent faire l'objet d'audits et d'inspections réglementaires sans que puisse être opposé le secret médical.

Un audit peut être réalisé à tout moment par des personnes mandatées par le promoteur et indépendantes des responsables de la recherche. Il a pour objectif de s'assurer de la qualité de la recherche, de la validité de ses résultats et du respect de la loi et des règlementations en vigueur.

Les personnes qui dirigent et surveillent la recherche acceptent de se conformer aux exigences du promoteur et à l’autorité compétente en ce qui concerne un audit ou une inspection de la recherche.

L’audit pourra s’appliquer à tous les stades de la recherche, du développement du protocole à la publication des résultats et au classement des données utilisées ou produites dans le cadre de la recherche.

13.6 Engagement de responsabilités de l’Investigateur Principal

Avant de démarrer la recherche, chaque investigateur fournira au représentant du promoteur de la recherche son curriculum vitæ personnel daté et signé, comportant son numéro RPPS. Le CV devra comprendre les participations antérieures à des recherches et les formations liées à la recherche clinique.

Chaque investigateur s'engagera à respecter les obligations de la loi et à mener la recherche selon les B.P.C., en respectant les termes de la déclaration d'Helsinki en vigueur.

L'investigateur principal de chaque centre participant signera un engagement de responsabilités (document type DRCI) qui sera remis au représentant du promoteur.
Les investigateurs et leurs collaborateurs signeront un formulaire de délégation de fonctions précisant le rôle de chacun.

14 ASPECTS ETHIQUES ET LEGAUX

14.1 Modalités d'information et de recueil du consentement des personnes se prêtant à la recherche

Conformément à l'article L1122-1-1 du Code de la santé publique, aucune recherche interventionnelle impliquant la personne humaine ne peut être pratiquée sur une personne sans son consentement libre et éclairé, recueilli par écrit après que lui a été délivrée l'information prévue à l'article L. 1122-1 du même code.

Un délai de réflexion de **3 jours** est laissé à la personne entre le moment où elle est informée et celui où elle signe le formulaire de consentement.

Le consentement libre, éclairé et écrit de la personne est recueilli par l'investigateur, ou par un médecin qui le représente avant l'inclusion de la personne dans la recherche et avant la réalisation de tout acte nécessité par la recherche interventionnelle impliquant la personne humaine.

La note d'information et une copie du formulaire de consentement daté et signé par la personne qui se prête à la recherche ainsi que par l'investigateur ou le médecin qui le représente sont remis à la personne préalablement à sa participation à la recherche.

En outre, l'investigateur précisera dans le dossier médical de la personne la participation de celle-ci à la recherche, les modalités de recueil de son consentement ainsi que les modalités de la délivrance de l'information en vue de le recueillir. Il conserve l'exemplaire original du formulaire du recueil du consentement de la personne daté et signé.

14.2 Interdiction pour la personne de participer à une autre recherche ou période d'exclusion **prévue à l'issue de la recherche, le cas échéant**

La période d'exclusion définie dans le cadre de cette recherche est de 12 mois, durée nécessaire à la cicatrisation complète des tissus prostatiques.
Pendant cette période le sujet ne peut pas participer à un autre protocole de recherche interventionnelle impliquant la personne humaine. Les sujets peuvent toutefois participer à d'autres recherches de type non interventionnel.

14.3 Indemnisation des sujets

Aucune indemnisation n'est prévue pour les patients en compensation des contraintes liées à la recherche.

14.4 Autorisation des lieux

La recherche se déroule dans des services de soins sur des personnes présentant une condition clinique pour laquelle les services ont compétence et nécessite des actes pratiqués

habituellement dans le cadre de leurs activités. Par conséquent, il n'est pas nécessaire d'avoir une autorisation de lieu spécifique pour la recherche.

15 OBLIGATIONS LÉGALES

15.1 Rôle du promoteur

L'Assistance publique hôpitaux de Paris (AP-HP) est le promoteur de cette recherche et par délégation la Délégation à la Recherche Clinique et à l'Innovation (DRCI) en assure les missions, conformément à l'article L.1121-1 du code de la santé publique. L'Assistance Publique - Hôpitaux de Paris se réserve le droit d'interrompre la recherche à tout moment pour des raisons médicales ou administratives; dans cette éventualité, une notification sera fournie à l'investigateur

15.2 Demande d'avis au comité de protection des personnes CPP

L'AP-HP en tant que promoteur obtient pour la recherche interventionnelle impliquant la personne humaine portant sur un dispositif médical ou sur un dispositif médical de diagnostic in vitro, préalablement à sa mise en œuvre l'avis favorable du CPP concerné, dans le cadre de ses compétences et conformément aux dispositions législatives et réglementaires en vigueur.

15.3 Demande d'autorisation à l'ANSM

L'AP-HP en tant que promoteur obtient pour la recherche interventionnelle impliquant la personne humaine portant sur un dispositif médical ou sur un dispositif médical de diagnostic in vitro, préalablement à sa mise en œuvre l'autorisation de l'ANSM, dans le cadre de ses compétences et conformément aux dispositions législatives et réglementaires en vigueur.

15.4 Engagement de conformité à la « Méthodologie de référence » MR 001

L'AP-HP, promoteur de la recherche, a signé un engagement de conformité à cette « Méthodologie de Référence ».

15.5 Modifications de la recherche

Toute modification substantielle apportée au protocole par l'investigateur coordonnateur, devra être transmise au promoteur pour approbation. Après cet accord, le promoteur devra obtenir préalablement à sa mise en œuvre un avis favorable du CPP et une autorisation de l'ANSM dans le cadre de leurs compétences respectives.
La note d'information et le formulaire de consentement pourront être révisés si nécessaire, notamment en cas de modification substantielle de la recherche ou de la survenue d'effets indésirables.

15.6 Rapport final de la recherche

Le rapport final de la recherche interventionnelle impliquant la personne humaine mentionné à l'article R1123-60 du CSP est établi et signé par le promoteur et l'investigateur. Un résumé du rapport rédigé selon le plan de référence de l'autorité compétente doit être transmis à l'autorité compétente dans un délai de un an, après la fin de la recherche, correspondant au terme de la participation de la dernière personne qui se prête à la recherche.

16 FINANCEMENT ET ASSURANCE

16.1 Source de financement

Cette recherche est financée par la société KOELIS et est promue par l'APHP (DRCI).

16.2 Assurance

Le Promoteur, souscrit pour toute la durée de la recherche une assurance garantissant sa propre responsabilité civile ainsi que celle de tout médecin impliqué dans la réalisation de la recherche. Il assure également l'indemnisation intégrale des conséquences dommageables à la recherche pour la personne qui s'y prête et ses ayants droit, sauf preuve à sa charge que le dommage n'est pas imputable à sa faute ou à celle de tout intervenant, sans que puisse être opposé le fait d'un tiers ou le retrait volontaire de la personne qui avait initialement consenti à se prêter à la recherche.

L'Assistance Publique- Hôpitaux de Paris (AP-HP) a pris une assurance auprès de la compagnie HDI-GERLING par l'intermédiaire de BIOMEDIC-INSURE pour toute la durée de la recherche, garantissant sa responsabilité civile ainsi que celle de tout intervenant (médecin ou personnel impliqué dans la réalisation de la recherche), conformément à l'article L.1121-10 du CSP.

17 REGLES RELATIVES A LA PUBLICATION

L'APHP sera obligatoirement mentionnée dans les affiliations du ou des auteurs des publications qui résulteront de cette recherche et l'AP-HP (DRCI) sera mentionnée comme promoteur.

17.1 Mention de l'affiliation de l'AP-HP pour les projets promus par l'AP-HP

L'institution AP-HP apparaîtra sous le sigle « AP-HP » en premier dans l'adresse suivi précisément par : AP-HP, hôpital, service, ville, code postal, France.
- Si un auteur a plusieurs affiliations, l'ordre dans lequel sont citées les institutions (AP-HP, Université, INSERM…) n'a pas d'importance
- Cependant, si la recherche est financée dans le cadre d'un appel d'offre interne de l'AP-HP, la première affiliation devrait être « AP-HP »
- Chacune de ces affiliations doit être identifiée par une adresse séparée par un point-virgule
- L'institution AP-HP doit apparaître sous le sigle « **AP-HP** » en premier dans l'adresse suivi précisément par : **AP-HP**, hôpital, service, ville, code postal, France

17.2 Mention du promoteur AP-HP (DRCI) dans les "acknowledgments" du manuscrit

Le promoteur de la recherche (Assistance Publique – Hôpitaux de Paris) sera mentionné dans les "acknowledgments" des manuscrits (« The sponsor was Assistance Publique – Hôpitaux de Paris (Délégation à la Recherche Clinique et à l'Innovation (DRCI)) »)

17.3 Mention du financeur dans les "acknowledgments" du manuscrit

Le financeur de la recherche (KOELIS) sera mentionné dans les "acknowledgments" des manuscrits («The study was funded by a grant from KOELIS, La Tronche, France »).

Cette recherche est enregistrée sur le site http://clinicaltrials.gov/ sous le n°*NCT03023345*.

18 *BIBLIOGRAPHIE*

1) Noldus J, Graefen M, Haese A, Henke RP, Hammerer P, HulandH. Stage migration in clinically localized prostate cancer. EurUrol 2000;38: 74-8.

2) Pardo Y, Guedea F, Aguilo F, et al. Quality-of-life impact of primary treatments for localized prostate cancer in patients without hormonal treatment. Journal of clinical oncology : official journal of the American Society of Clinical Oncology 2010;28:4687-96.

3) Bottomley D, Ash D, Al-Qaisieh B*, et al.* Side effects of permanent I125 prostate seed implants in 667 patients treated in Leeds. Radiotherapy and oncology : journal of the European Society for Therapeutic Radiology and Oncology 2007;82:46-9.

4) Cornud F, Khoury G, Bouazza N, Beuvon F, Peyromaure M, Flam T, Zerbib M, Legmann P, Delongchamps NB. Tumor target volume for focal therapy of prostate cancer-does multiparametric magnetic resonance imaging allow for a reliable estimation? J Urol. 2014 May;191(5):1272-9.

5) Delongchamps NB, Rouanne M, Flam T, Beuvon F, Liberatore M, Zerbib M, et al. Multiparametric magnetic resonance imaging for the detection and localization of prostate cancer: combination of T2-weighted, dynamic contrast enhanced and diffusion-weighted imaging. BJU Int 2011; 107: 1411—8.

6) Delongchamps NB, Beuvon F, Eiss D, Flam T, Muradyan N, Zerbib M, et al. Multiparametric MRI is helpful to predict tumor focality, stage, and size in patients diagnosed with unilateral low-risk prostate cancer. Prostate Cancer Prostatic Dis 2011; 14: 232—7.

7) Lanz C, Cornud F, Beuvon F, Lefèvre A, Legmann P, Zerbib M, Delongchamps NB. Gleason score determination with TRUS-MRI fusion guided prostate biopsies: are we gaining in accuracy? J Urol. 2015 Jul 9. pii: S0022-5347(15)04338-4. doi: 10.1016/j.juro.2015.07.021.

8) Delongchamps NB, Lefèvre A, Bouazza N, Beuvon F, Legman P, Cornud F. Detection of significant prostate cancer with magnetic resonance targeted biopsies--should transrectal ultrasound-magnetic resonance imaging fusion guided biopsies alone be a standard of care? J Urol. 2015 Apr;193(4):1198-204.

9) Delongchamps NB, Peyromaure M, Schull A, Beuvon F, Bouazza N, Flam T, Zerbib M, Muradyan N, Legman P, Cornud F. Prebiopsy magnetic resonance imaging and prostate cancer detection: comparison of random and targeted biopsies. J Urol. 2013; 189: 493-9.

10) Institut National du Cancer. Dynamique d'évolution des taux de mortalité des principaux cancers en France. http://www.e-cancer.fr/toutes-les-actualites/81/4703-la-baisse-de-la-mortalite-par-cancers-saccelere-en-france.

11) Jemal A, Siegel R, Xu J, Ward E. Cancer statistics, 2010. CA Cancer J Clin 2010; 60: 277-300.

12) Delongchamps NB, Singh A, Haas GP. The role of prevalence in the diagnosis of prostate cancer. Cancer Control 2006; 13: 158-68.

13) Salomon L, Bastide C, Beuzeboc P, Cormier L, Fromont G, Hennequin C, Mongiat-Artus P, Peyromaure M, Ploussard G, Renard-Penna R, Rozet F, Azria D, Coloby P, Molinié V, Ravery V, Rebillard X, Richaud P, Villers A, Soulié M; Les membres du CCAFU. [CCAFU Recommendations 2013: Prostate cancer]. Prog Urol. 2013 Nov;23 Suppl 2:S69-101.

14) D'Amico AV, Whittington R, Malkowicz SB, et al. Biochemical outcome after radical prostatectomy, external beam radiation therapy, or interstitial radiation therapy for clinically localized prostate cancer. JAMA : the journal of the American Medical Association 1998; 280: 969-74.

15) Heidenreich A, Bellmunt J, Bolla M, et al.EAU guidelines on prostate cancer. Part 1: screening, diagnosis, and treatment of clinically localised disease. European urology 2011;59:61-71.

16) Parker C, Muston D, Melia J, Moss S, Dearnaley D. A model of the natural history of screen-detected prostate cancer, and the effect of radical treatment on overall survival. British journal of cancer 2006;94:1361-8.

17) Pickles T, Ruether JD, Weir L, Carlson L, Jakulj F. Psychosocial barriers to active surveillance for the management of early prostate cancer and a strategy for increased acceptance. BJU international 2007;100:544-51.

18) Latini DM, Hart SL, Knight SJ*, et al.* The relationship between anxiety and time to treatment for patients with prostate cancer on surveillance. The Journal of urology 2007;178:826-31; discussion 831-2.

19) Turkbey B, Mani H, Shah V, RastinehadAR, Bernardo M, Pohida T, et al. Multiparametric 3T prostate magnetic resonance imaging to detect cancer: histopathological correlation using prostatectomy specimens processed in customized magnetic resonance imaging based molds. J Urol 2011; 186: 1818—24.

20) Park BK, Park JW, Park SY, Kim CK, Lee HM, Jeon SS, et al. Prospective evaluation of 3-T MRI performed before initial transrectal ultrasound-guided prostate biopsy in patients with high prostate-specific antigen and no previous biopsy. AJR Am J Roentgenol 2011; 197: W876—81.

21) Haffner J, Lemaitre L, Puech P, Haber GP, Leroy X, Jones JS, et al. Role of magnetic resonance imaging before initial biopsy: comparison of magnetic resonance imaging-targeted and systematic biopsy for significant prostate cancer detection. BJU Int 2011; 108: 171—8.

22) Villers A, Puech P, Mouton D, Leroy X, Ballereau C, Lemaitre L. Dynamic contrast enhanced, pelvic phased array magnetic resonance imaging of localized prostate cancer for predicting tumor volume: correlation with radical prostatectomy findings. J Urol 2006;176:2432—7.

23) Dickinson L, Ahmed HU, Allen C, Barentsz JO, Carey B, Futterer JJ, Heijmink SW, Hoskin PJ, Kirkham A, Padhani AR, Persad R, Puech P, Punwani S, Sohaib AS, Tombal B, Villers A, van der Meulen J, Emberton M. Magnetic resonance imaging for the detection, localisation, and characterisation of prostate cancer: recommendations from a European consensus meeting. Eur Urol. 2011; 59: 477-94.

24) Portalez D, Mozer P, Cornud F, Renard-Penna R, Misrai V, ThoulouzanM,Malavaud B. Validation of the European society of urogenital radiology scoringsystem for prostate cancer diagnosis on multiparametric magnetic resonanceimaging in a cohort of repeat biopsy patients. Eur Urol. 2012; 62: 986-96.

25) Natarajan S, Marks LS, Margolis DJA, Huang J, Macairan ML, Lieu P, et al. Clinical application of a 3D ultrasound-guided prostate biopsy system. UrolOncol 2011; 29: 334—42.

26) Baumann M, Mozer P, Daanen V, Troccaz J. Prostate biopsy tracking with deformation estimation. Med Image Anal. 2012 Apr;16(3):562-76. Epub 2011 May 17.

27) Audenet F, Rouprêt M, Mozer P. [Evolution of modern imaging and development of targeted prostatic biopsies for the diagnosis of prostate cancer]. Prog Urol. 2011 May;21Suppl 3:S93-5.

28) Mozer P, Baumann M, Chevreau G, Moreau-Gaudry A, Bart S, Renard-Penna R, et al. Mapping of transrectalultrasonographicprostate biopsies: quality control and learning curve assessmentby image processing. J Ultrasound Med 2009; 28: 455-60.

29) Ukimura O, Desai OM, Palmer S, Valencerina S,Gross M, Abreu AL, AronM, Gill IS. 3-Dimensional Elastic Registration System of Prostate BiopsyLocation by Real-Time 3-Dimensional TransrectalUltrasoundGuidanceWith Magnetic Resonance/Transrectal UltrasoundImage Fusion. J Urol 2012; 187: 1088-1086.

30) Lecornet E, Moore C, Ahmed HU, Emberton M. Focal therapy for prostate cancer: fact or fiction? Urologic oncology 2010; 28: 550-6.

31) Onik G. The male lumpectomy: rationale for a cancer targeted approach for prostate cryoablation. A review. Technology in cancer research & treatment 2004; 3: 365-70.

32) Bahn D, de Castro Abreu AL, Gill IS*, et al.* Focal Cryotherapy for Clinically Unilateral, Low-Intermediate Risk Prostate Cancer in 73 Men with a Median Follow-Up of 3.7 Years. European urology 2012.

33) Ahmed HU, Hindley RG, Dickinson L*, et al.* Focal therapy for localised unifocal and multifocal prostate cancer: a prospective development study. The lancet oncology 2012.

34) Feijoo ER, Sivaraman A, Barret E, Sanchez-Salas R, Galiano M, Rozet F, Prapotnich D, Cathala N, Mombet A, Cathelineau X. Focal High-intensity Focused Ultrasound Targeted Hemiablation for Unilateral Prostate Cancer: A Prospective Evaluation of Oncologic and Functional Outcomes. Eur Urol. 2015 Jul 8. pii: S0302-2838(15)00521-7. doi: 10.1016/j.eururo.2015.06.018.

35) Ghai S, Louis AS, Van Vliet M, Lindner U, Haider MA, Hlasny E, Spensieri P, Van Der Kwast TH, McCluskey SA, Kucharczyk W, Trachtenberg J. Real-Time MRI-Guided Focused Ultrasound for Focal Therapy of Locally Confined Low-Risk Prostate Cancer: Feasibility and Preliminary Outcomes. AJR Am J Roentgenol. 2015; 205(2):W177-84. doi: 10.2214/AJR.14.13098.

36) Azzouzi AR, Barret E, Bennet J, Moore C, Taneja S, Muir G, Villers A, Coleman J, Allen C, Scherz A, Emberton M. TOOKAD® Soluble focal therapy: pooled analysis of three

phase II studies assessing the minimally invasive ablation of localized prostate cancer. World J Urol. 2015; 33: 945-53.

37) Azzouzi AR, Barret E, Moore CM, Villers A, Allen C, Scherz A, Muir G, de Wildt M, Barber NJ, Lebdai S, Emberton M. TOOKAD(®) Soluble vascular-targeted photodynamic (VTP) therapy: determination of optimal treatment conditions and assessment of effects in patients with localised prostate cancer. BJU Int. 2013; 112: 766-74.

38) Lindner U, Lawrentschuk N, Weersink RA, Davidson SR, Raz O, Hlasny E, Langer DL, Gertner MR, Van der Kwast T, Haider MA, Trachtenberg J. Focal laser ablation for prostate cancer followed by radical prostatectomy: validation of focal therapy and imaging accuracy. Eur Urol. 2010 Jun;57(6):1111-4.

39) Lepor H, Llukani E, Sperling D, Fütterer JJ. Complications, Recovery, and Early Functional Outcomes and Oncologic Control Following In-bore Focal Laser Ablation of Prostate Cancer. Eur Urol. 2015 May 12. pii: S0302-2838(15)00331-0. doi: 10.1016/j.eururo.2015.04.029.

40) van den Bos W, Muller BG, Ahmed H, Bangma CH, Barret E, Crouzet S, Eggener SE, Gill IS, Joniau S, Kovacs G, Pahernik S, de la Rosette JJ, Rouvière O, Salomon G, Ward JF, Scardino PT. Focal therapy in prostate cancer: international multidisciplinary consensus on trial design. Eur Urol. 2014; 65: 1078-83.

41) Cary KC, Cowan JE, Sanford M, Shinohara K, Perez N, *et al.* Predictors of pathologic progression on biopsy among men on active surveillance for localized prostate cancer: the value of the pattern of surveillance biopsies. *Eur Urol* 2014; 66: 337-42.

42) Nam RK, Saskin R, Lee Y, Liu Y, Law C, Klotz LH, et al. Increasing hospital admission rates for urological complications after transrectal ultrasound guided prostate biopsy. J Urol 2010; 183: 963—8.

43) Naber KG, Bishop MC, Bjerklund-Johansen TE, Botto H, Cek M, Grabe M, Lobel B, Palou J, Tenke P. Guidelines on the management of urinary and male genital tract infections. European association of urology 2006

19 LISTE DES ADDENDA

19.1 Liste des Investigateurs

Coordonnées du lieu de recherche	Titre	Prénom Nom	Téléphone / e-mail
Service d'urologie, hôpital Cochin	Dr	Nicolas Barry Delongchamps	01 58 41 27 64<br>nicolas.barry-delongchamps@aphp.fr
Service d'urologie, hôpital Cochin	Dr	Alexandre Schull	01 58 41 27 57<br>alexandre.schull@aphp.fr

19.2 Formulaire de notification des Evenements Indesirables Graves

Direction de l'Organisation Médicale et des relations avec les Universités (DOMU)<br><br>Délégation à la Recherche Clinique et à l'Innovation (DRCI)	ASSISTANCE PUBLIQUE HÔPITAUX DE PARIS<br><br>**Formulaire de notification d'un Evènement Indésirable Grave (EIG) survenant au cours d'une Recherche Interventionnelle Impliquant la Personne Humaine portant sur un Dispositif Médical (DM)**	**PARTIE RESERVEE AU PROMOTEUR**<br><br>**REFERENCE VIGILANCE :**<br><br>Référence GED : REC-DTYP-0270

Dès la prise de connaissance de l'EIG par l'investigateur, ce formulaire doit être dûment complété (3 pages), signé et retourné sans délai au secteur Vigilance de la DRCI-Siège par télécopie au +33 (0)1 44 84 17 99

Notification initiale ☐ Suivi d'EIG ☐ N° du suivi |__|__|

**1. Identification de la recherche**	
**Acronyme : FOSTINE**	**Date de notification :** |__|__||__|__||_2_|_0_|__|__| jj mm aaaa
**Code de la Recherche : P160301**<br>**Risque : D**	**Date de prise de connaissance de l'EIG par l'investigateur :** |__|__||__|__||_2_|_0_|__|__| jj mm aaaa
**Titre complet de la Recherche:**<br>**Etude évaluant la faisabilité et la tolérance d'un traitement focal transrectal par micro-ondes de la tumeur index des patients ayant un cancer de la prostate à risque faible ou risque intermédiaire bas de progression**	

**2. Identification du centre investigateur**	
Nom de l'établissement : Hôpital Cochin<br>Ville et code postal : PARIS - 75014<br>Service : Service d'urologie	Investigateur (nom/prénom) : ....<br>Tél :.......... Fax :..........

**3. Identification et antécédents de la personne se prêtant à la recherche**		
Référencede la personne :|__|__|__|-|__|__|__|__|-|__|-|__| n°centre- n° ordre de sélection- initiale nom - initiale prénom		**Antécédents médicaux-chirurgicaux/familiaux pertinents pour l'évaluation du cas (joindre un CRH anonymisé le cas échéant) :**<br>..........<br>..........<br>..........<br>..........<br>..........<br>..........<br>..........
Sexe : ☒ M ☐ F	Date de naissance :<br>|__|__||__|__| |__|__|__|__| jj mm aaaa	
Poids : |__|__|__| kg<br>Taille : |__|__|__| cm	Age : |__|__|__|ans	
Date de signature du consentement :|__|__| |__|__||_2_|_0_|__|__| jj mm aaaa		
Date de l'intervention : |__|__| |__|__||_2_|_0_|__|__|		

4. Description du (ou des DM) expérimental(aux) ***(barrer l'encadré si DM expérimental(aux) non utilisé(s))***

Dénomination commune / Dénomination commerciale (si marquage CE)	Modèle / Version (y compris de logiciel)	N° de série	N° de lot	Dates de début et de fin de l'utilisation du DM chez le participant	Dose	Zone traitée
Système d'ablation par micro-onde TATO	....	....	....	Durée : |__|__|hh |__|__|min<br>Conforme à l'abaque :<br>☐ oui ☐ non	|__|__| watts<br>Conforme à l'abaque :<br>☐ oui ☐ non	Diamètre : |__|__| mm<br>Longueur : |__|__| mm
Station TRINITY	....	....	....	Durée : |__|__|hh |__|__|min		

Si accessoire(s), consommable(s)

Désignation	Modèle	N° de série	N° de lot	Nom du fabricant	Date de péremption *(jj/mm/aaaa)*
..........<br>..........	....	....	....	....	|__|__||__|__||_2_|_0_|__|__|

Acronyme : FOSTINE

Référencede la personne se prêtant à la recherche : |__|__|__|-|__|__|__|__|-|__|-|__|

n°centre- n° ordre de sélection- initiale - initiale
nom prénom

PARTIE RESERVEE AU PROMOTEUR
REFERENCE VIGILANCE :
Référence GED : REC-DTYP-0270

....	....	....	....	....	|__|__||__|__||_2_|_0_|__|__|

5.a Procédures et actes ajoutés par la recherche *(ex. : biopsies, IRM, …)* *(barrer l'encadré si procédures et actes non réalisés)*	Date de réalisation *(jj/mm/aaaa)*	Chronologie	
		Avant la survenue de l'EIG	Après la survenue de l'EIG
IRM	|__|__||__|__||_2_|_0_|__|__|	☐	☐
....	|__|__||__|__||_2_|_0_|__|__|	☐	☐

5.b Médicament(s) non expérimental(aux) (ME) ou produit(s) assimilé(s) [préciser le(s)quel(s)] avant la survenue de l'EIG
(barrer l'encadré si traitement non débuté)

Nom commercial (de préférence) ou Dénomination Commune Internationale	Voie(1)	Posologie (préciser l'unité ex : mg/j)	Date de début *(jj/mm/aaaa)*	En cours(2)	Date de fin *(jj/mm/aaaa)*
Préparation à l'intervention					
Ofloxacine	....	....	|__|__| |__|__| |_2_|_0_|__|__|	☐	|__|__| |__|__| |_2_|_0_|__|__|
Sulfamethoxazole/triméthoprime	....	....	|__|__| |__|__| |_2_|_0_|__|__|	☐	|__|__| |__|__| |_2_|_0_|__|__|
Microlax®	....	....	|__|__| |__|__| |_2_|_0_|__|__|	☐	|__|__| |__|__| |_2_|_0_|__|__|
Anesthésie					
....	....	....	|__|__| |__|__| |_2_|_0_|__|__|	☐	|__|__| |__|__| |_2_|_0_|__|__|
Traitement de soutien					
Paracetamol	....	....	|__|__| |__|__| |_2_|_0_|__|__|	☐	|__|__| |__|__| |_2_|_0_|__|__|
Perfalgan	....	....	|__|__| |__|__| |_2_|_0_|__|__|	☐	|__|__| |__|__| |_2_|_0_|__|__|
Acupan®	....	....	|__|__| |__|__| |_2_|_0_|__|__|	☐	|__|__| |__|__| |_2_|_0_|__|__|
Ondansetron	....	....	|__|__| |__|__| |_2_|_0_|__|__|	☐	|__|__| |__|__| |_2_|_0_|__|__|

6. Médicament(s) concomitant(s)au moment de l'EIG, à l'exclusion de ceux utilisés pour traiter l'événement indésirable *(compléter le tableau ci-après et si nécessaire l'annexe relative aux médicaments concomitants ou barrer l'encadré si non applicable)*

➪Annexe jointe au présent formulaire : ☐ Oui ☐ Non

Nom commercial (de préférence) ou Dénomination Commune Internationale	Voie(1)	Posologie (préciser l'unité ex : mg/j)	Dates d'administration *(du jj/mm/aa au jj/mm/aa)*	En cours (2)	Indication	***Action prise*** *0 : poursuite sans modification de la posologie* *1 : arrêt* *2 : diminution de la posologie* *3 : augmentation de la posologie* *4 : ne sais pas*	***Causalité de l'EIG*** *0 : non lié au médicament* *1 : lié au médicament* *2 : ne sais pas*
			du |__|__||__|__| |__|__| au |__|__||__|__| |__|__|	☐			
			du |__|__||__|__| |__|__| au |__|__||__|__| |__|__|	☐			

(1) Voie d'administration : VO=voie orale ; IM=Intramusculaire ; IV=intraveineuse ; SC=sous-cutanée ou autre (à préciser) (2) En cours au moment de la survenue de l'EIG

7. Evènement indésirable grave [EIG]

Diagnostic : ☐ Définitif ☐ Provisoire
....
....
....

Date de survenue des premiers symptômes : |__|__||__|__||_2_|_0_|__|__|
Préciser lesquels :
....
....
....

Organe(s) concerné(s) :
....
....
....
....
....
....
....
....

Acronyme : FOSTINE

Référencede la personne se prêtant à la recherche : |__|__|__|-|__|__|__|__|-|__|-|__|
n°centre- n° ordre de sélection- initiale - initiale
nom prénom

PARTIE RESERVEE AU PROMOTEUR

REFERENCE VIGILANCE :

Référence GED : REC-DTYP-0270

Fait-il suite à un incident technique(3) lié au(x) DM ?
☐Non ☐ Oui Si oui, décrire l'incident :...
....
....
Date de survenue de l'incident : |__|__||__|__||_2_|_0_|__|__|
Des actions techniques ont-elles été prises vis-à-vis du ou des DM ?
☐Non ☐ Oui Si oui, préciser :...
(3) Code de la Santé Publique : articles L. 5212-2, R. 5212-14 à 16

Critères de gravité :
☐ Nécessite ou prolonge l'hospitalisation :*du* |__|__||__|__||_2_|_0_|__|__|
au |__|__||__|__||_2_|_0_|__|__|☐ *en cours*
☐ Décès
☐ Mise en jeu du pronostic vital
☐ Incapacité ou handicap important ou durable
☐ Anomalie ou malformation congénitale
☐ Autre(s) critère(s) médicalement significatif(s), préciser :
....
....

Date d'apparition de l'EIG :
|__|__||__|__||_2_|_0_|__|__|
jjmm aaaa
Heure de survenue :|__||__|hh|__||__|min
☐ *donnée manquante*

Délai entre la date de la dernière utilisation ou de la mise en place du ou des DM ou la date de la procédure/acte associé et la date de survenue de l'EIG :
|__||__| / |__||__| |__||__|
jjhh min

Degré de sévérité:
☐Léger ☐Modéré ☐Sévère

Classification Clavien-Dindo : |__|__|

L'évènement a-t-il conduit à une interruption de l'utilisation du (ou des) DM expérimental (aux) ?
☐ Non ☐ Oui Date : |__|__| |__|__| |_2_|_0_|__|__|☐ Non applicable
L'arrêt de l'utilisation du DM expérimental a été : ○ Provisoire ○ Définitif
Le cas échéant, date de réutilisation du DM expérimental :|__|__| |__|__| |_2_|_0_|__|__|
Récidive de l'EIG après réutilisation :○ Non ○ Oui Date : |__|__| |__|__| |_2_|_0_|__|__|
○ Non applicable

Des mesures symptomatiques ont-elles été prises ?

☐ Non ☐ Oui Date :|__|__||__|__||_2_|_0_|__|__|
Préciser : ....

Evolution de l'événement

☐**Décès** Date :|__|__||__|__||_2_|_0_|__|__|
○ sans relation avec l'EIG jj mm aaaa
○en relation avec l'EIG

☐**Résolu :** Date :|__|__||__|__||_2_|_0_|__|__|
○sans séquelles jj mm aaaa
○avec séquelles, préciser lesquelles :
.... |__|__| |__|__|
hh min

☐**Sujet non encore rétabli**, préciser :
○Etat stable○Amélioration○Aggravation

☐**Evolution inconnue**

8. Autre(s) étiologie(s) envisagée(s)

☐ Non ☐Oui Si oui, préciser :...
....
....

9. Examen(s) complémentaire(s) réalisé(s)

☐ Non☐Oui Si oui, préciser date, nature et résultats : [joindre les bilans anonymisés]..............................
....
....

10. Selon l'investigateur, l'événement indésirable grave est (plusieurs cases possibles)

<u>Lié à la recherche</u> :

☐Oui : ○**au DM de la recherche :le(s)quel(s) ?**

Système d'ablation thermique TATO® ☐Relationcertaine☐ Relation probable ☐ Relation possible ☐Relation improbable (non exclu)

○**au(x) geste(s) de mise en œuvre du ou des DM : traitement par micro-onde par voie transrectale**

☐Relationcertaine☐ Relation probable ☐ Relation possible ☐Relation improbable (non exclu)

○**à la (aux) procédure(s)/acte(s) de la recherche : la/le(s)quel(les) ?**

Préparation à l'intervention ☐Relationcertaine☐ Relation probable ☐ Relation possible ☐Relation improbable (non exclu)

Anesthésie générale ☐Relationcertaine☐ Relation probable ☐ Relation possible ☐Relation improbable (non exclu)

Immobilisation ☐Relationcertaine☐ Relation probable ☐ Relation possible ☐Relation improbable (non exclu)

IRM ☐Relationcertaine☐ Relation probable ☐ Relation possible ☐Relation improbable (non exclu)

☐ Non : ○**à la progression de la maladie faisant l'objet de la recherche:** cancer de la prostate

○**à un (ou plusieurs) médicament(s) concomitant(s) administré(s), le(s)quel(s) :**……………………………………

○**à une maladie intercurrente, laquelle :** ……………………………………

○**autre, préciser :** ……………………………………

Notificateur	Investigateur	Tampon du service :
Nom et fonction :<br>Signature	Nom :<br>Signature	

19.3 Marquages CE

Notified Body n° 0426

ITALCERT

ITALCERT S.r.l.
viale Sarca, 336 - IT 20126 MILANO MI
tel. +39 0266104876 fax +39 0266101479
E-mail italcert@italcert.it

CERTIFICATE n° 294/E-00-00-DM
(in compliance with Annex II of the Directive 93/42/EEC)

ITALCERT

certifies that

Total Quality Assurance System
applied for the design and manufacturing of "Medical Devices" - MD -

by the manufacturer

BIOMEDICAL S.r.l.
via G.B. Lulli, 43 - 50144 FIRENZE (FI) - ITALY

in the headquarters located in

via G.B. Lulli, 43
50144 FIRENZE (FI) - ITALY

complies with the requirements stated in

Directive 93/42/EEC - Annex II (excluding point 4)

and authorizes the same manufacturer to mark

CE **0426**

in compliance with the criteria defined in Annex XII of the Directive 93/42/EEC
the MD reported in Annex 1 of this Certificate

dr. ing. Roberto Cusolito
MANAGING DIRECTOR

First issue date
2015-07-27

Expire date
2020-07-26

This certificate must be published only in integral form and accompanied by its Annex 1

ITALCERT S.r.l.
viale Sarca, 336 - IT 20126 MILANO MI
tel. +39 0266104876 - fax +39 0266101479
E- mail italcert@italcert.it
Notified Body n° 0426

Annex 1 to Certificate n° 294/E-00-00-DM

Active medical devices utilising non-ionizing radiations and accessories

DESCRIPTION	CODE
COMPLETE SYSTEM FOR MICROWAVE THERMAL ABLATION (class IIb)	TATO1
APPLICATORS (class IIa)	TTP xx yy β D(G)LR -αα
STERILE EXTENSION CABLES (class I STERILE)	TTP CA EX S -αα

Milan, 2015-07-27

dr. ing. Roberto Cusolito
Managing Director

KOELIS

KOELIS SAS
DECLARATIONS
N°4601-11-04-02_V2.0

Fabricant : **KOELIS**
5, Avenue du Grand Sablon
38700 LA TRONCHE
FRANCE

Classe du dispositif : I

Directive applicable : Directive 93/42/CE

Désignation du produit concerné : GUIDES REUTILISABLES

Référence du produit concerné : GPK-XXX avec X = chiffre entre 0 et 9

Référence du dossier technique : 4601-10-01

DECLARATION

Je soussigné, M. Antoine LEROY, Président du fabricant KOELIS, déclare et garantit que les dispositifs médicaux cités ci-dessus :

- n'incorporent pas, comme partie intégrante, de substance considérée comme médicament, comme indiqué au point 7.4, 1° alinéa de l'annexe I de la directive 93/42/CE,
- n'incorporent pas, comme partie intégrante, de substance dérivée du sang humain, comme indiqué au point 7.4, 3° alinéa de l'annexe I de la directive 93/42/CE,
- ne sont pas fabriqués à partir de tissus d'origine animale.

Nom: Antoine LEROY.

Fonction: Président

Date: le 06 / [illegible] / 2015

Lieu: Grenoble.

Tampon et signature:

KOELIS SAS
5 av. du Grand Sablon - 38700 La Tronche
Tél. 04 76 63 75 87 - Mail info@koelis.com
SIREN 492 218 375 - APE 7112B
SAS au capital de 902 600 €

Période de validité : 5 ans

KOELIS 5 Avenue du Grand Sablon 38700 La Tronche France Tél : + 33(0)476637587 Fax : + 33(0)476549561
Société par Actions Simplifiée au capital de 902 600€ N°SIRET 492 218 375 000 11 Code APE : 7112B
www.koelis.com

ZERTIFIKAT ◆ CERTIFICATE ◆ 認証証書 ◆ СЕРТИФИКАТ ◆ CERTIFICADO ◆ CERTIFICAT

swiss TS

CERTIFICATE

The certification body of Swiss TS Technical Services AG
hereby confirms that the company

Baitella AG
CH-8050 Zürich

has introduced and applies a management system for

Design, manufacturing and sales of articulated arms with quick central fixation for industrial and medical applications

according to:

**ISO 9001:2008**	**Quality management**
**ISO 13485:2003**	**for medical devices**

	ISO 9001	ISO 13485
Registration number:	**04-314-078**	**04-314-049**
Initial certification:	**11.11.1997**	**11.11.1997**
Recertification:	**22.10.2015**	**22.10.2015**
Valid until:	**10.11.2018**	**10.11.2018**

Heinrich A. Bieler
Head of the certification body

Wallisellen, 29.10.2015
The certification body
of Swiss TS Technical Services AG
A SVTI and TÜV SÜD company

SWISS ACCREDITATION sas.admin.ch SCESm 0013

Certified System swiss TS ISO 9001 - ISO 13485

LNE
Le progrès, une passion à partager

Certification
Médical-Santé

Notified Body N° 0459

ATTESTATION / *CERTIFICATE* **N° 29385 rev. 0**
Délivrée à Paris le 03 juin 2015
Issued in Paris on June 3rd, 2015

ATTESTATION CE / *EC CERTIFICATE*

Approbation du Système Complet d'assurance Qualité/ *Approval of full Quality Assurance System*
ANNEXE II excluant le point 4 Directive 93/42/CEE relative aux dispositifs médicaux
ANNEX II excluding section 4 Directive 93/42/EEC concerning medical devices
Pour les dispositifs de classe III, un certificat CE de conception est requis
For class III devices, a EC design certificate is required

Fabricant / *Manufacturer*

KOELIS
5 avenue du Grand Sablon
38700 LA TRONCHE FRANCE

Catégorie du(des) dispositif(s) / *Device(s) category*

Système d'imagerie échographique destiné au guidage d'interventions et au diagnostic dans le domaine de l'urologie.

Ultrasound imaging system intended to operation guiding and diagnosis in the field of urology.

Voir détails sur addendum / See attachment for additional information

Le LNE/G-MED atteste qu'à l'examen des résultats figurant dans le rapport référencé P115838-E, P115838-Doc, le système d'assurance qualité - pour la conception, la production et le contrôle final - des dispositifs médicaux énumérés ci-dessus est conforme aux exigences de l'annexe II excluant le point 4 de la Directive 93/42/CEE.

LNE/G-MED certifies that, on the basis of the results contained in the file referenced P115838-E, P115838-Doc, the quality system - for design, manufacturing, and final inspection - of medical devices listed here above complies with the requirements of the Directive 93/42/EEC, annex II excluding section 4

La validité du présent certificat est soumise à une vérification périodique ou imprévue
The validity of the certificate is subject to periodic or unexpected verification

Début de validité / *Effective date* : **June 3rd, 2015 (included)**
Valable jusqu'au / *Expiry date* : **December 19th, 2017 (included)**

Laboratoire national de métrologie et d'essais LNE

For the General Director
Laurence DAGALLIER
Deputy Director

GMED_[illegible]

LNE - 29385 rev. 0

19.4 Questionnaire IPSS (questions 1 à 7)

	Jamais	Environ 1 fois sur 5	Environ 1 fois sur 3	1 fois sur 2	2 fois sur 3	Presque toujours
Au cours du dernier mois avec quelle fréquence avez-vous eu la sensation que votre vessie n'était pas complètement vidée ?	0	1	2	3	4	5
Au cours du dernier mois avec quelle fréquence avez-vous eu besoin d'uriner moins de 2 heures après une miction ?	0	1	2	3	4	5
Au cours du dernier mois avec quelle fréquence avez-vous eu une interruption du jet d'urine c'est à dire démarrage de la miction puis arrêt puis redémarrage ?	0	1	2	3	4	5
Au cours du dernier mois après avoir ressenti le besoin d'uriner, avec quelle fréquence avez-vous eu des difficultés à retenir votre miction ?	0	1	2	3	4	5
Au cours du dernier mois avec quelle fréquence avez-vous eu une diminution de la taille et de la force du jet ?	0	1	2	3	4	5
Au cours du dernier mois avec quelle fréquence avez-vous du forcer ou pousser pour commencer à uriner ?	0	1	2	3	4	5
	Jamais	1 fois	2 fois	3 fois	4 fois	5 fois
Au cours du dernier mois écoulé, combien de fois par nuit, en moyenne, vous êtes-vous levé pour uriner (entre le moment de votre coucher le soir et celui de votre lever définitif le matin ?	0	1	2	3	4	5

19.5 Questionnaire IPSS-QDV (score de qualité de vie, question 8)

Si vous deviez vivre le restant de votre vie avec cette manière d'uriner, diriez-vous que vous en seriez :

Très satisfait	Satisfait	Plutôt satisfait	Partagé : ni satisfait si ennuyé	Plutôt ennuyé	Ennuyé	Très ennuyé
0	1	2	3	4	5	6

19.6 Questionnaire IIEF

Au cours des six derniers mois :

A quel point étiez-vous sûr de pouvoir avoir une érection et de la maintenir ?

0 Pas sûr du tout

1 Pas très sûr

2 Moyennement sûr

3 Sûr

4 Très sûr

Lorsque vous avez eu des érections à la suite de stimulations sexuelles, avec quelle fréquence votre pénis a-t'il été suffisamment rigide (dur) pour permettre la pénétration ?

0 Je n'ai pas été stimulé sexuellement

1 Presque jamais ou jamais

2 Rarement (beaucoup moins que la moitié du temps)

3 Quelquefois (environ la moitié du temps)

4 La plupart du temps (beaucoup plus que la moitié du temps)

5 Presque tout le temps ou tout le temps

Pendant vos rapports sexuels, avec quelle fréquence avez-vous pu rester en érection après avoir pénétré votre partenaire (introduction du pénis dans le vagin) ?

0 Je n'ai pas essayé d'avoir de rapports sexuels

1 Presque jamais ou jamais

2 Rarement (beaucoup moins que la moitié du temps)

3 Quelquefois (environ la moitié du temps)

4 La plupart du temps (beaucoup plus que la moitié du temps)

5 Presque tout le temps ou tout le temps

Pendant vos rapports sexuels, à quel point vous a-t'il été difficile de rester en érection jusqu'à la fin de ces rapports ?

0 Je n'ai pas essayé d'avoir de rapports sexuels

1 Extrèmement difficile

2 Très difficile

3 Difficile

4 Un peu difficile

5 Pas difficile

Lorsque vous avez essayé d'avoir des rapports sexuels, avec quelle fréquence en avez-vous été satisfait ?

0 Je n'ai pas essayé d'avoir de rapports sexuels

1 Presque jamais ou jamais

2 Rarement (beaucoup moins que la moitié du temps)

3 Quelquefois (environ la moitié du temps)

4 La plupart du temps (beaucoup plus que la moitié du temps)

5 Presque tout le temps ou tout le temps

19.7 Questionnaire MSHG-EjD

Ces questions sont en rapport avec vos éjaculations au cours du dernier mois

Avec quelle fréquence avez-vous été capable d'éjaculer au cours de vos rapports sexuels ?	Tout le temps (5)	Le plus souvent (4)	Une fois sur deux (3)	Moins d'une fois sur deux (2)	Jamais / Absence d'éjaculation (1)	
Comment considérez-vous la force de votre éjaculation ?	Toujours aussi forte (5)	Un peu moins forte qu'avant (4)	Plutôt moins forte qu'avant (3)	Moins forte qu'avant (2)	Beaucoup moins forte qu'avant (1)	Ne peut pas éjaculer (0)
Comment évaluez-vous la quantité de sperme lorsque vous éjaculez	Toujours autant (5)	Un peu moins qu'avant (4)	Plutôt moins qu'avant (3)	Moins qu'avant (2)	Beaucoup moins qu'avant (1)	Ne peut pas éjaculer (0)
Si vous avez eu des difficultés ou n'avez pas réussi à éjaculer, en avez-vous été gêné ?	Pas de problème avec mes éjaculations (0)	Pas du tout gêné (1)	Un peu gêné (2)	Modérément gêné (3)	Très gêné (4)	Extrêmement gêné (5)

19.8 Abaques

19.8.1 Abaque pour le thérapie focale par ablation micor-onde

KOELIS biomedical

Abaque

pour la thérapie focale par ablation micro-onde.

Objectifs du document

Ce document a pour objet la création d'un abaque permettant de prédire le volume traité par un rayonnement micro-onde appliqué sur un tissu en fonction de la puissance et du temps du rayonnement. Il décrit les méthodes mathématiques utilisées pour analyser les données, présente les données exploitées, précise les résultats des analyses statistiques et présente enfin l'abaque obtenu sous forme de tableaux et sous forme graphique.

Ce travail est issu d'une collaboration entre les sociétés Biomedical et Koelis.

Auteur : Eric GAUDARD (KOELIS)

Table des matières

1. Introduction 69
2. Modèle utilisé 69
3. Régression linéaire 70
4. Applications 70
4.1 Première étude : analyse de sensibilité 70
4.2 Seconde étude : création de l'abaque 74

1. Introduction

Lors d'une thérapie focale par ablation, une énergie (puissance × temps) est appliquée au tissu afin de le nécroser sur un certain volume. Il est indispensable de pouvoir estimer l'énergie à appliquer en fonction du volume de nécrose requis pour le traitement. Nous pouvons identifier 3 moyens de relier un volume cible à une énergie :

- **Résoudre les équations** de tous les phénomènes en jeu (rayonnement, diffusion, convection de la chaleur...). . Problèmes : **1)** Il est à priori difficile de caractériser ces différents phénomènes. **2)** Le temps de calcul.
- **Déterminer une grille** en multipliant les points de mesure, de telle sorte que chaque volume cible ait un couple (puissance, temps) associé. Problèmes : **1)** Pour obtenir une grille raisonnablement résolue (20 × 20 par exemple), il faudrait 400 mesures. **2)** Chacune de ces 400 mesures présenterait une erreur. Si l'erreur d'une mesure exploitée est de 4 mm, on gardera cette erreur de 4 mm lors de l'opération.
- **Déterminer une relation mathématique** à partir des mesures effectuées en utilisant une régression linéaire. Avantages : **1)** Cela permet d'obtenir un couple (puissance, temps) pour tout volume cible. **2)** Un nombre réduit de mesures est suffisant. Cela n'empêche pas de multiplier les points de mesure pour gagner en précision. **3)** L'erreur est lissée en exploitant l'ensemble des mesures. **4)** Cette erreur peut être évaluée. **5)** Des analyses statistiques permettent également de quantifier l'effet de différents paramètres sur les mesures (type de tissu, taille de l'applicateur...).

Nous décrivons dans ce document comment déterminer une relation mathématique f reliant le volume traité V_t à un couple (puissance P, temps T) :

$$V_t = f(P,T).$$

La forme de la fonction f doit être modélisée. Nous pourrions par exemple choisir un modèle polynomial :

$$V_t = a_1PT + a_2P^2T^2 + a_3P^2T + a_4PT^2.$$

Une régression linéaire permettrait, à partir de plusieurs mesures, de déterminer les quatre paramètres a_i et d'obtenir ainsi une relation mathématique entre V_t, P et T. Ce modèle n'est pas le plus adapté ici. Nous proposons d'utiliser un modèle physique plus proche des phénomènes en jeux.

2. Modèle utilisé

La base du modèle proposé repose sur deux hypothèses :

- L'énergie décroit en exponentielle dans le tissu. Tous les phénomènes associés à la propagation de la chaleur dans le tissu (rayonnement, convection, conduction) sont modélisés par un paramètre unique d'atténuation α.
- Il existe une énergie seuil E_s à partir de laquelle le tissu devient nécrosé.

Écrivons $E(x)$ l'énergie du rayonnement à une distance x du point d'émission. Cette variable peut se décomposer comme le produit de la puissance du rayonnement $P(x)$ en ce point et du temps de l'exposition T au rayonnement :

$$E(x) = P(x)T.$$

Nous supposons que la puissance d'amplitude P_0 au point d'émission décroit exponentiellement :

$$P(x) = P_0e^{-\alpha x}.$$

Nous supposons qu'il existe une énergie E_s à partir de laquelle le tissu est nécrosé. Il existe alors, selon ce modèle, un diamètre $x = D$ pour lequel l'énergie $E(x)$ est égale à E_s :

$$TP_0e^{-\alpha D} = E_s,$$

ou encore, en écrivant que $E = TP_0$:

$$Ee^{-\alpha D} = E_s.$$

Ainsi, le tissu est nécrosé pour tout $x < D$.

Lors des essais, nous mesurons l'énergie $E = P_0T$ et le diamètre de nécrose D. Nous souhaitons déterminer les paramètres E_s et α qui caractérisent le tissu. Une régression linéaire est alors utilisée.

3. Régression linéaire

Nous souhaitons écrire la relation entre nos variables sous la forme d'une équation linaire :

$$Y = mX + p.$$

Nous devons pour cela effectuer quelques opérations :

$$e^{-\alpha D} = \frac{E_s}{E}.$$

$$\ln(e^{-\alpha D}) = \ln(\frac{E_s}{E}).$$

$$-\alpha D = \ln(\frac{E_s}{E}).$$

$$-\alpha D = \ln(E_s) - \ln(E).$$

$$\boldsymbol{D = \ln(E)/\alpha - \ln(E_s)/\alpha}.$$

La relation n'est pas polynomiale et une régression linéaire ne peut pas directement être effectuée. Nous allons donc rapporter cette équation logarithmique à une équation linéaire à l'aide d'un changement de variable.

$$Y = D.$$

$$X = \ln(E)\ .$$

$$m = \frac{1}{\alpha}.$$

$$p = -\ln(E_s)/\alpha.$$

Le résultat (a, b) de la régression linéaire est alors utilisé pour calculer (E_s, α) :

$$\boldsymbol{\alpha = \frac{1}{m},}$$

$$\boldsymbol{E_s = e^{-p\alpha}.}$$

Une fois les paramètres (E_s, α) connus, nous pouvons déterminer l'énergie E nécessaire à l'obtention d'une nécrose de diamètre D :

$$\boldsymbol{E = E_s e^{\alpha D}.}$$

4. Applications

La méthode d'analyse présentée plus haut est appliquée sur deux jeux de données issus d'un travail collaboratif entre les sociétés Biomedical et Koelis. Tout d'abord, un premier jeu de données obtenues sur différents types de tissus avec différents applicateurs est analysé dans le but d'évaluer la sensibilité du traitement à ces différents paramètres. Enfin, les données obtenues lors d'une nouvelle étude, avec un seul type de tissu et un type d'applicateur et dans des conditions identiques sont exploitées dans le but de déterminer l'abaque. Les calculs sont effectués avec un langage informatique dédié aux statistiques : **R**.

4.1 Première étude : analyse de sensibilité

Les données sont présentées dans le *Tableau 1*. Ces données ont été effectués avec différents paramètres ce qui permet d'effectuer une analyse de sensibilité à ces paramètres. Le résultat de la régression linéaire entre le diamètre et l'énergie est illustré sur la *Figure 3*. Les deux mêmes graphiques sont présentés avec en haut une coloration des points selon la taille de l'applicateur, en bas une coloration selon le type de viande. Bien que le nombre de points dans chaque catégorie soit faible, on relève que le modèle utilisé semble être correct et confirme la pertinence d'un modèle reliant linéairement D et $X = \ln(P_0 T)$. On note une dispersion assez importante, probablement liée à la faible précision de la mesure de la distance D avec le décimètre. Cette erreur pourrait également être due à un manque de répétabilité de l'opération (conditions des mesures différentes, opérateurs différents, température du tissu différente, etc.). Le *Tableau 2* présente les statistiques sur les résidus. Le résidu moyen entre la valeur mesurée et la valeur estimée est égal à 2.92. Les paramètres évalués pour cette première étude sont $E_s = 4.19$ et $\alpha = 0.15$. On relève de cette première étude que le diamètre du volume de traitement semble peu sensible au type de viande traité et au type d'applicateur. Ce résultat est important, car il confirme l'intérêt de l'utilisation des micro-ondes pour traiter indifféremment différents types de tissus.

Nous avons jusqu'ici estimé le diamètre, et non pas le volume du traitement. Pour obtenir le volume, il faut également estimer la longueur de la nécrose. Si le rayonnement était ponctuel, le volume serait simplement une sphère de diamètre D. Seulement, la source de rayonnement n'est pas ponctuelle. Il s'agit plutôt d'une source linéique. Dans ce cas, il faut noter que nous sommes à priori à l'abri d'interférences, car la longueur d'onde (environ 10 cm pour des micro-ondes ayant une fréquence de 3 GHz par exemple), est bien plus grande que la taille de la source. On peut donc estimer que la longueur augmente simplement de manière linéaire avec le diamètre :

$$L = aD + b,$$

où b est la longueur de la source et a est le paramètre reliant linéairement la longueur et le diamètre. Une nouvelle régression linéaire sur les mesures de L et de D est effectuée pour tester cette hypothèse. Le résultat est présenté sur la *Figure 4*. L'ordonnée à l'origine est estimée à 3.3 mm. La pente a est égale à 1.29. Cette figure montre qu'une telle approximation pourra se montrer efficace pour estimer la longueur de la nécrose, et donc le volume. Pour le calcul du volume, nous supposons que la zone traitée est de forme ellipsoïdale.

Du fait de la présence d'une dispersion non négligeable sur les mesures de cette première étude, une seconde étude a été effectuée. Ces nouvelles mesures seront utilisées pour la construction de l'abaque.

P_0 [W]	T [min]	D [mm]	L [mm]	Applicateur	Viande
**60**	10	31	50	14	Foie de porc
**30**	10	28	40	14	Foie de porc
**15**	20	28	31	14	Foie de bœuf
**30**	10	29	42	14	Foie de porc
**20**	10	26	30	14	Rein de porc
**20**	10	25	30	14	Rein de porc
**20**	15	30	40	14	Rein de porc
**15**	20	30	42	14	Rein de porc
**20**	10	20	30	17	Rein de porc
**20**	10	22	32	14	Rein de porc
**45**	10	40	58	14	Rein de porc
**35**	20	25	50	17	Rein de porc
**40**	10	33	43	17	Rein de porc
**20**	10	29	44	18	Rein de porc
**15**	10	25	35	18	Rein de porc
**10**	5	14	22	18	Rein de porc
**10**	2.5	12	20	18	Rein de porc
**30**	5	20	30	18	Rein de porc
**20**	10	20	30	18	Rein de porc
**20**	5	25	30	14	Rein de porc

Tableau 1 : Données de la première étude.

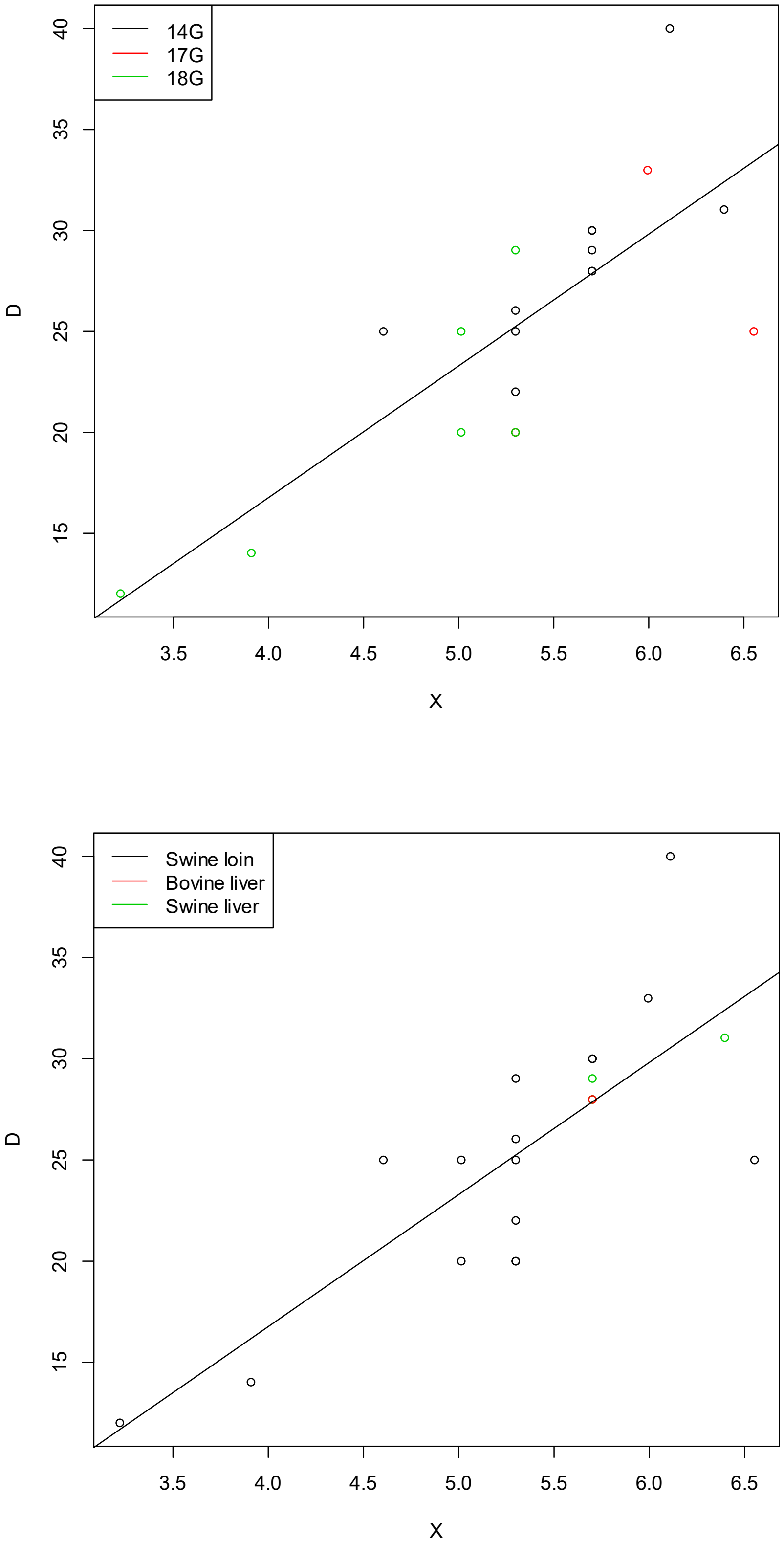

Figure 3 : Régression linaire $D = m\,X + p$ avec $X = ln(P_0\,T)$ en [mm] pour la première étude.

Min	1Q	Médiane	Moyenne	3Q	Max
**0.13**	1.04	2.16	2.92	3.91	9.48

Tableau 2: Statistiques sur les résidus [mm] de l'estimation de D (première étude).

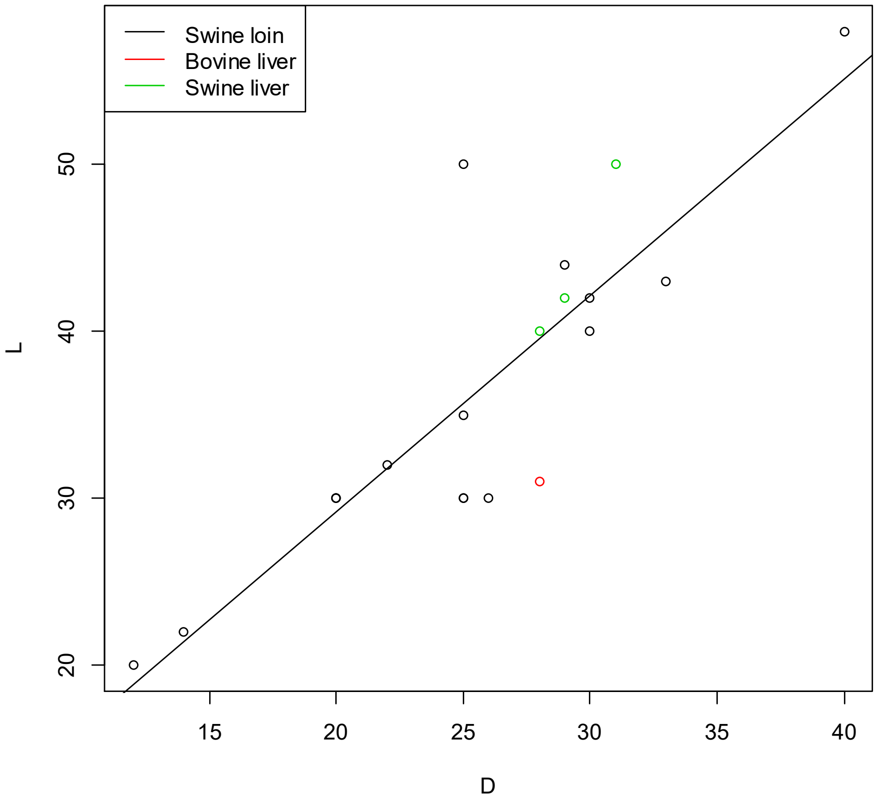

Figure 4 : Recherche d'une relation linéaire entre le diamètre D et la longueur L de la nécrose en [mm]. Résultat : a = 1.29 et p = 3.3 mm.

4.2 Seconde étude : création de l'abaque

Étant donnée la dispersion constatée sur les premières mesures, une seconde étude a été effectuée. Ces nouveaux essais ont été effectués le même jour, dans des conditions de températures identiques, et par la même personne pour minimiser les erreurs de mesure. Un seul type de tissu est ici étudié (du foie de porc). Les données sont résumées dans le *Tableau 3*. Le résultat de la régression linéaire est illustré sur la *Figure 5*. On constate que la loi logarithmique est à nouveau bien respectée. Les résidus sont ici faibles, avec une moyenne de 0.74 mm d'erreur et une erreur maximale de 2.12 mm (voir *Tableau 4*). Les paramètres évalués pour cette seconde étude sont $E_s = 5.8$ et $\alpha = 0.1587$ et sont proches de ceux obtenus lors de la première étude. Une régression linéaire entre le diamètre et la longueur est illustrée sur la *Figure 6*. Là encore, le modèle linéaire se montre pertinent et permet de prédire la longueur associée à un diamètre donné. La

Figure 7 présente l'abaque obtenu à partir de ce second jeu de données et du modèle logarithmique. On peut choisir, à l'aide de ce graphique, un couple (puissance, temps) correspondant à un diamètre cible.

P_0 [W]	T [min]	D [mm]	L [mm]
**20**	5	15	25
**20**	1	8	19
**15**	5	14	23
**10**	2	4.5	12
**10**	5	11	18
**15**	2	10	20
**15**	1	5	12
**10**	1	3	5

Tableau 3 : Données de la seconde étude.

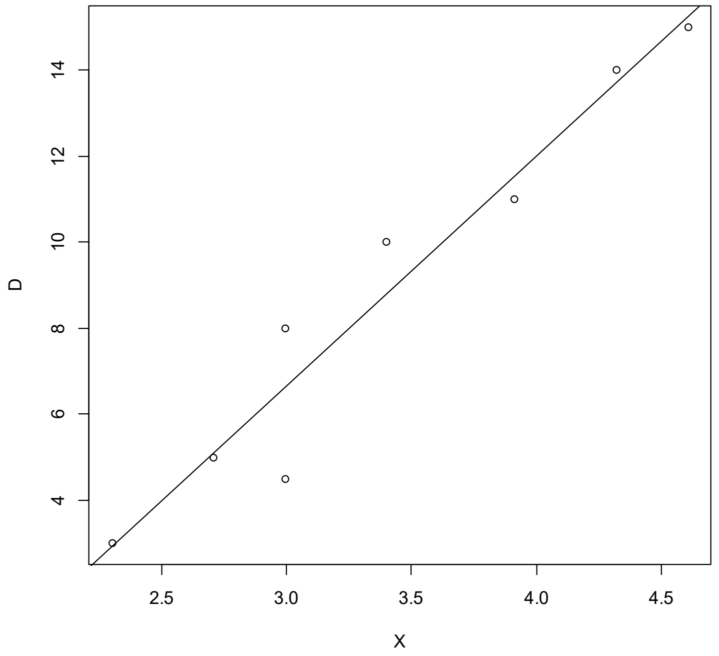

Figure 5 : Régression linaire $D = m\,X + p$ avec $X = ln(P_0\,T)$ en [mm] (seconde étude).

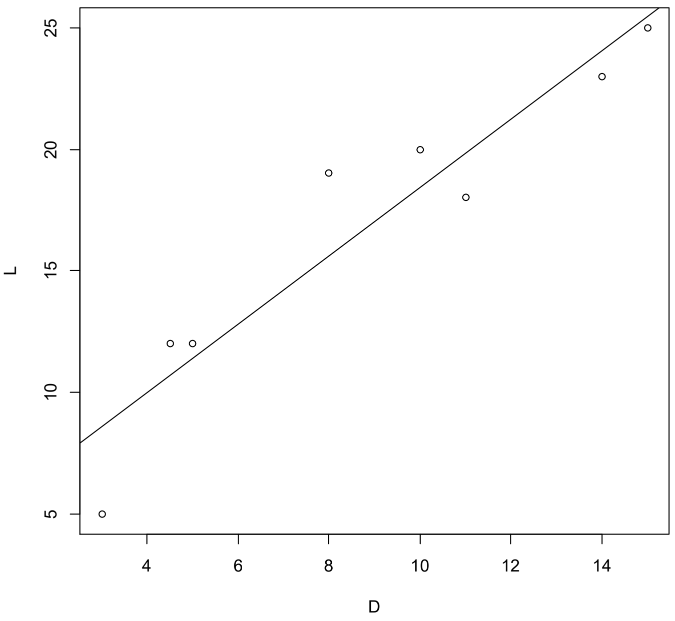

Figure 6 : Recherche d'une relation linéaire entre le diamètre D et la longueur L de la nécrose en [mm] (seconde étude). Résultat : a = 1.4 et b = 4.37

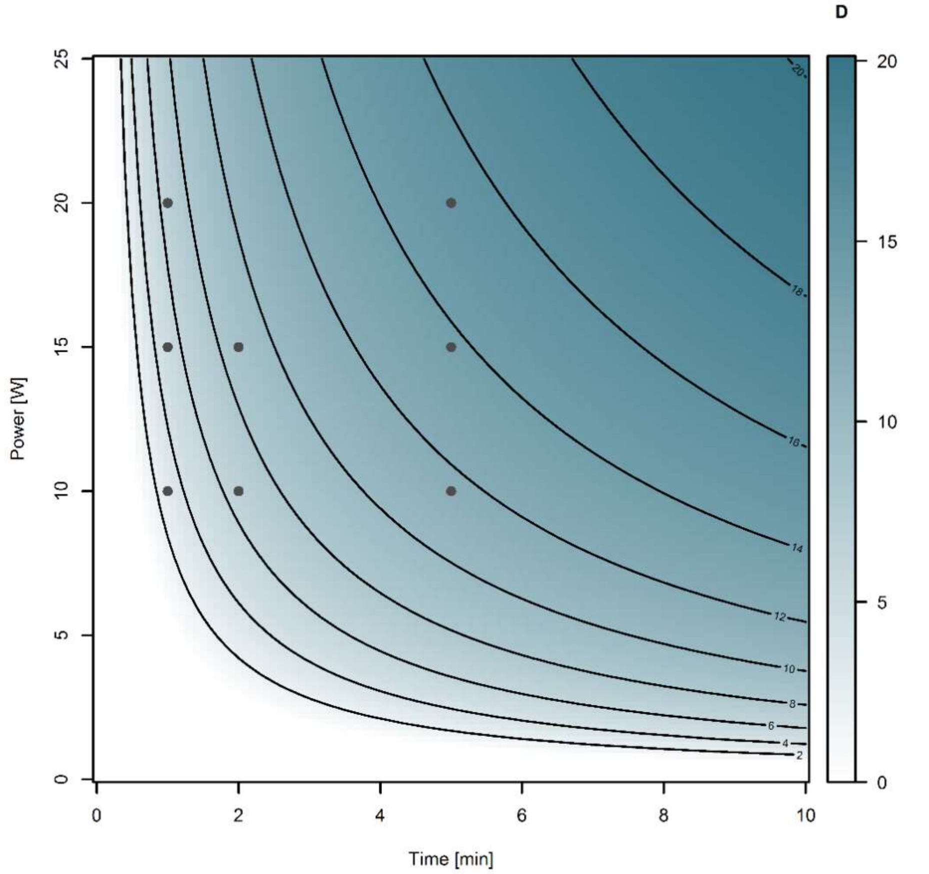

Figure 7 : Abaque obtenu avec les données de la seconde étude. Il s'agit de l'image de l'équation $D = ln(E)/\alpha - ln(E_s)/\alpha$ *avec* $E_s = 5.8$ *et* $\alpha = 0.187$*. Les points représentent les mesures qui ont été utilisées pour obtenir cet abaque.*

Min	1Q	Médiane	Moyenne	3Q	Max
**0.08**	0.2	0.42	0.74	1.25	2.12

Tableau 4 : Statistiques sur les résidus de l'estimation de D en [mm] (seconde étude).

Des abaques sous forme de tableaux sont présentés *Tableau 5* et *Tableau 6*. Le volume de nécrose est déterminé à partir :

- du diamètre de nécrose D issu de la formule logarithmique
- de la longueur L de nécrose déterminée par le modèle linéaire

Enfin, le résultat est présenté sous forme graphique sur la *Figure 8* pour la puissance recommandée par le constructeur (10W), et pour 3 temps d'exposition différents couvrant des volumes cibles adaptés à notre application.

T [min]	1	2	3	4	5	6	7	8	9	10
D [mm]	2.9	6.6	8.8	10.3	11.5	12.5	13.3	14	14.7	15.2
L [mm]	8.5	13.7	16.7	18.9	20.6	21.9	23.1	24.1	25	25.8
V_n [cm^3]	0.04	0.3	0.7	1.1	1.4	1.8	2.1	2.5	2.8	3.1

Tableau 5 : Abaque sous forme de tableau généré à partir des mesures de la seconde étude et du modèle logarithmique, pour une puissance de 10W. Cette puissance est celle recommandée par BioMedical avec un applicateur de 18G.

T [min]	1	2	3	4	5	6	7	8	9	10
D [mm]	5.1	8.8	11	12.5	13.7	14.7	15.5	16.2	16.8	17.4
L [mm]	11.5	16.7	19.8	21.9	23.6	25	26.1	27.1	28	28.8
V_n [cm^3]	0.16	0.68	1.24	1.79	2.32	2.81	3.29	3.73	4.16	4.57

Tableau 6 : Abaque sous forme de tableau généré à partir des mesures de la seconde étude et du modèle logarithmique, pour une puissance de 15W. Cette puissance est la puissance maximale utilisable avec un applicateur de 18G.

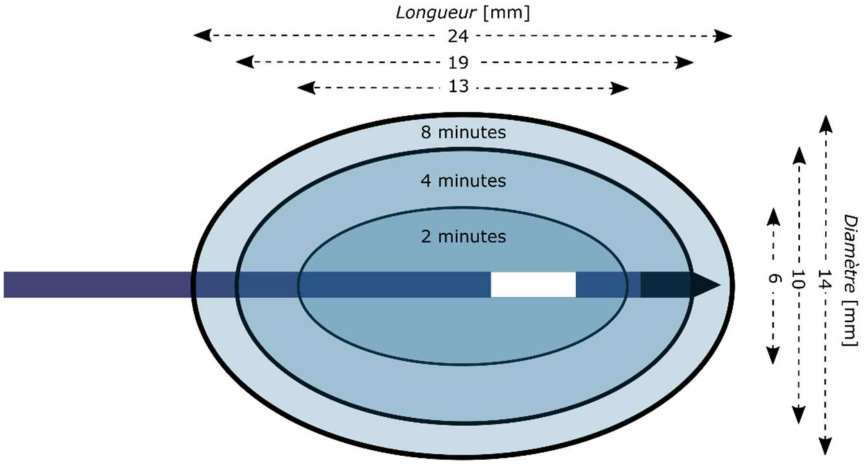

Figure 8: Abaque sous forme graphique (seconde étude) pour une puissance de 10W.

19.8.2 Abaque pour le traitement micro-onde

Abaque FOSTINE

pour le traitement microonde

Ce document synthétise le résultat d'une étude préclinique du traitement microonde sur viande. Cette étude a permis, à travers plus de 100 mesures, d'évaluer l'effet du type de viande, de la température, de la quantité d'eau des tissus, du diamètre de l'applicateur, ou encore de l'utilisation d'un câble d'extension. Cette étude a permis la mise au point d'un modèle prédictif des dimensions de la zone traitée.

L'abaque présenté dans ce document est généré suite à des essais complémentaires permettant d'évaluer l'effet de la quantité d'eau, de la température, et de l'utilisation d'un câble d'extension sur la zone traitée. Les outils statistiques sont les mêmes que ceux utilisés pour la première version de l'abaque (FOSTINE_Abaque_v2.1_EGa.pdf).

L'utilisation d'un câble d'extension, du fait des déperditions en puissance, diminue d'un millimètre environ le diamètre de l'ablation. L'expression analytique de cette déperdition est connue et permet une correction efficace des dimensions prédictives. L'injection d'eau a un effet relativement faible sur les dimensions de l'ablation. Cette variation est indiquée dans l'abaque.

Les présents tableaux ont été générés à partir de 16 mesures sur **rôti de porc** uniquement, (viande qui dans notre étude se rapproche le plus de la prostate), avec un applicateur de **18G**, avec un câble d'extension et les dimensions ont été corrigées pour correspondre à des tissus ayant une température de 37°C. En bon accord avec les précédents abaques obtenus, ces tableaux prenant en compte plus de paramètres (eau, câble, température) sont néanmoins plus précis.

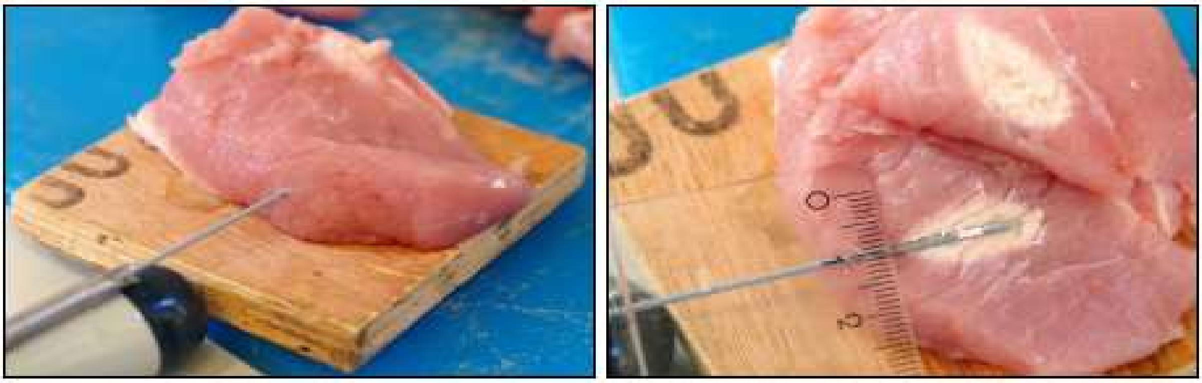

Photographies d'un essai sur rôti de porc. Puissance de 15W. Temps de 1.5 minute. Sans câble d'extension. Température de viande 28°C. D^{in}=9mm ; D^{out}=10mm ; L^{in}=18mm ; L^{out}=19mm.

	Temps				Incertitude
Dimensions	2 min	2.5 min	3 min	3.5 min	
D^{in} [mm]	8.6	9.5	10.3	10.9	± 0.6 mm
D^{out} [mm]	10.1	11.2	12.1	12.9	± 0.8 mm
L^{in} [mm]	16.0	17.0	18.0	18.7	± 1.7 mm
L^{out} [mm]	18.7	19.6	20.3	21.0	± 2.9 mm
Avec eau [mm]	-1.3	-0.7	-0.2	0.2	±1.3 mm

Dimensions prédictives du traitement pour une puissance de **10W.**

	Temps				Incertitude
Dimensions	1.5 min	2 min	2.5 min	3 min	
D^{in} [mm]	9.1	10.3	11.2	12.0	± 0.6 mm
D^{out} [mm]	10.7	12.1	13.3	14.2	± 0.8 mm
L^{in} [mm]	18.2	19.6	20.6	21.6	± 1.7 mm
L^{out} [mm]	21.4	22.5	23.5	24.3	± 2.9 mm
Avec eau [mm]	-1	-0.2	0.4	0.9	±1.3 mm

Dimensions prédictives du traitement pour une puissance de **15W.**

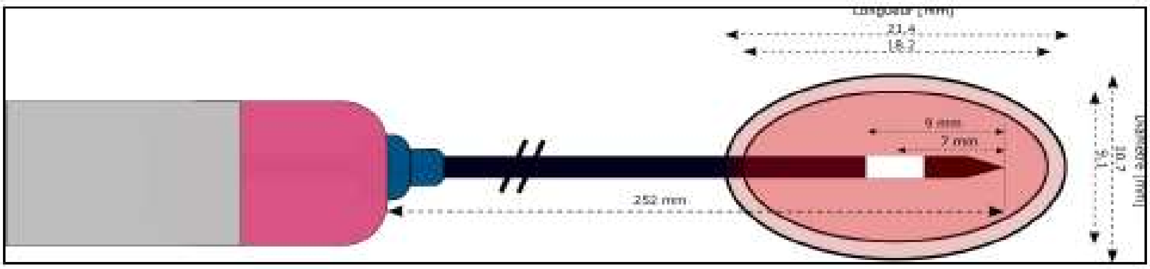

Dimensions de l'applicateur + dimensions de l'ablation pour 15W et 1.5 min.

19.9 Carte patient

ASSISTANCE PUBLIQUE HÔPITAUX DE PARIS

CARTE PATIENT

Merci de garder cette carte en permanence avec vous

Nom :... Prénom : ....

Code d'identification patient : 01-000 __-__ (N° d'inclusion – initiales N-P)

Je participe à la recherche interventionnelle : **FOSTINE**

« Etude évaluant la faisabilité et la tolérance d'un traitement focal transrectal par micro-ondes de la tumeur index des patients ayant un cancer de la prostate à risque faible ou risque intermédiaire bas de progression»

dont le promoteur est l'Assistance Publique – Hôpitaux de Paris

Dans le cadre de mon cancer de la prostate, j'ai reçu un traitement focal par micro-ondes délivrées par voie transrectale le __ __ / __ __ / 2017.

CARTE PATIENT

Merci de garder cette carte en permanence avec vous

Je suis suivi par le Dr Nicolas BARRY DELONGCHAMPS (urologue) à l'Hôpital Cochin, service d'urologie

En cas d'Urgence, contacter le médecin qui vous suit dans le cadre de la recherche

Téléphones. : de 8h à 17h : 01 58 41 27 64
de 17h à 8h : 01 58 41 41 41 (puis demander l'urologue d'astreinte)

FOSTINE_Carte patiente_v1.0 du 14/06/2017_EGT
