# Supplementary material for: Feasibility and safety of targeted focal microwave ablation of the index tumor in patients with low to intermediate risk prostate cancer: Results of the FOSTINE trial
Source: PLoS One. 2021 Jul 14;16(7):e0252040. doi: 10.1371/journal.pone.0252040 (PMC8279354; doi:10.1371/journal.pone.0252040)
Supplement: S4 File — (PDF) [file pone.0252040.s005.pdf]

"Study evaluating the feasibility and tolerance of focal transrectal microwave treatment of the index tumor of patients with prostate cancer at low risk or low intermediate risk of progression"

FOSTINE

INTERVENTIONAL RESEARCH PROTOCOL INVOLVING THE HUMAN PERSON, RELATING TO A MEDICAL DEVICE OR A MEDICAL DEVICE FOR IN VITRO DIAGNOSIS

Version N°3.0 of the 07 MARCH 2018

Ref number : P160301 / N°IDRCB : 2016-A01065-46

Investigator coordinnator : Nicolas BARRY DELONGCHAMPS
Service d'urologie
Hôpital Cochin
Tél 01 58 41 27 64
Courriel : nicolas.barry-delongchamps@aphp.fr

Scientific director : Alexandre SCHULL
Service d'urologie
Hôpital Cochin
Tél : 01 58 41 27 48
Courriel : alexandre.schull@aphp.fr

Promotor : AP-HP et par délégation : Délégation à la Recherche Clinique et à l'Innovation (DRCI)
Hôpital Saint-Louis
1, avenue Claude Vellefaux
Référent projet DRCI-Siege: Karine GOUDE-ORY
Courriel : karine.goude@aphp.fr

Structure in charge of Research :
Unité de Recherche Clinique (URC) Necker Cochin
Pavillon Tarnier, 89 rue d'Assas 75006 Paris
Référent projet DRCI-URC : Clotilde SIMON
Courriel : clotilde.simon@aphp.fr

Délégation à la Recherche Clinique et à l'Innovation (DRCI)
Hôpital Saint Louis 75010 PARIS

Signature page of an interventional research protocol involving the human person relating to a medical device or an in vitro diagnostic medical device

Ref number : P160301

Title : Study evaluating the feasibility and tolerance of focal transrectal microwave treatment of the index tumor of patients with prostate cancer at low risk or low intermediate risk of progression-FOSTINE

Version N°3.0 of the 07 MARCH 2018

The research will be conducted in accordance with the protocol, good practices in force and the laws and regulations in force.

The investigator coordonnator :

Dr Nicolas BARRY DELONGCHAMPS<br>Service d'urologie<br>Hôpital Cochin<br>Paris	Date : ……………/………/……….<br>Signature :
**The Scientific Director** :	
Dr Alexandre SCHULL<br>Service d'urologie<br>Hôpital Cochin<br>Paris	Date : ……………/………/……….<br>Signature :
**The promotor**	
Assistance Publique – Hôpitaux de Paris<br>Délégation à la Recherche Clinique et à l'Innovation (DRCI)<br>Hôpital Saint Louis<br>1 avenue Claude Vellefaux<br>75010 PARIS	Date : ……………/………/……….<br>Signature :

The research received a favorable agreement from the Ile de France III CPP on 04/04/2017 and an authorization from the ANSM on 05/05/2017.

TABLE OF CONTENTS

1 SYNOPSIS 6

2 SCIENTIFIC JUSTIFICATION OF THE RESEARCH 9

2.1 Research hypotheses 9
2.2 Description of knowledge relating to the pathology concerned 9
2.3 Summary of preclinical experiments and clinical trials concerned 13
2.4 Description of the population to be studied and justification for its choice 14
2.5 Name and description of the medical device 14
2.6 Description and justification of the methods of use of the medical device 14
2.7 Summary of foreseeable and known benefits and risks for those undergoing research 15
2.7.1 Foreseeable benefits 15
2.7.2 Foreseeable risks 15

3 GOALS 17

3.1 Main objective 17
3.2 Secondary objectives 17

4 RESEARCH DESIGN 17

4.1 Precise statement of primary and secondary endpoints 17
4.2 Description of the research methodology 18

5 RESEARCH PROGRESS 18

5.1 Selection visit : V-1 18
5.2 V isit inclusion V0 19
5.3 Research follow-up visits 20
5.4 End of research visit : V4 21
5.5 Expected duration of people's participation, description of the chronology and duration of the research. 21
5.6 Summary table of the research chronology 21
5.7 Care-research distinction 22
5.8 Shutdown rules 22

6 ELIGIBILITY CRITERIA 24

6.1 Inclusion criteria 24
6.2 Non-inclusion criteria 24
6.3 Recruitment methods 24

7 MEDICAL DEVICE USED IN RESEARCH AND TREATMENTS, PROCEDURES, STRATEGIES ASSOCIATED WITH THE USE OF THE DEVICE 25

7.1 Description of medical devices 25
7.2 Description of non-experimental treatments 29
7.3 Description of the traceability elements that accompany the investigational drug (s) 29
7.4 Authorized and prohibited treatments (drug, non-drug, surgical), including rescue drugs 29
7.5 Methods of monitoring adherence to treatment 29

8 EFFICIENCY EVALUATION 30

8.1 Description of evaluation parameters 30
8.2 Methods and schedule for measuring, collecting and analyzing parameters for evaluating effectiveness 30

9 COMMITTEES SPECIFIC RESEARCH **31**

9.1 Scientific committee 31
9.2 Steering committee 31
9.3 Critical Events Validation Committee 31

10 SAFETY ASSESSMENT - RISKS AND CONSTRAINTS ADDED BY RESEARCH **31**

10.1 Description of the safety assessment parameters 31
10.2 Methods and schedule for measuring, collecting and analyzing safety assessment parameters 32
Post-intervention surveillance unit : 32
Multiparametric MRI of the prostate : 32
Assessment of sexual tolerance : 32
Assessment of urinary tolerance : 32
10.3 Definitions 32
10.4 Roles of the investigator 34
10.5 Roles of the promoter 37
10.6 Independent Supervisory Committee 40

11 DATA MANAGEMENT **40**

11.1 Data collection methods 40
11.2 Identification of the data collected directly in the FIUs which will be considered as source data 40
11.3 Right of access to source data and documents 41
11.4 Data processing and retention of documents and data 41
11.5 Ownership data 42

12 STATISTICAL ASPECTS **42**

12.1 Assumptions for calculating the number of subjects required and result 42
12.2 Description of the planned statistical methods including the schedule for the planned interim analyzes 43

13 QUALITY CONTROL AND ASSURANCE **43**

13.1 General organization 43
13.2 Quality control 44
13.3 Observation book 44
13.4 Management of non-conformities 45
13.5 Audit / inspections 45
13.6 Responsibilities of the Principal Investigator 45

14 ETHICAL AND LEGAL ASPECTS **45**

14.1 Procedures for informing and obtaining the consent of persons participating in the research 45
14.2 Prohibition for the person to participate in another research or exclusion period provided for at the end of the research, if applicable 46
14.3 Compensation of subjects 46
14.4 Authorization of the premises 46

15 LEGAL OBLIGATIONS **46**

15.1 Role of the promoter 46
15.2 Request for an opinion from the CPP personal protection committee 46
15.3 Authorization request from ANSM 47
15.4 Commitment to comply with the “ Reference Methodology ” MR 001 47
15.5 Research modifications 47
15.6 Final research report 47

16 FINANCING AND INSURANCE **47**

16.1 SOURCE OF FUNDING 47
16.2 INSURANCE........ 47

17 PUBLICATION RULES **47**

17.1 MENTION OF THE AP-HP AFFILIATION FOR PROJECTS PROMOTED BY THE AP-HP 48
17.2 MENTION OF THE AP-HP PROMOTER (DRCI) IN THE ACKNOWLEDGMENTS OF THE MANUSCRIPT........ 48
17.3 MENTION OF THE FUNDER IN THE ACKNOWLEDGMENTS OF THE MANUSCRIPT 48

18 BIBLIOGRAPHY **49**

1 SYNOPSIS

Complete title	Study evaluating the feasibility and tolerance of focal transrectal microwave treatment of the index tumor of patients with prostate cancer at low risk or low intermediate risk of progression
Acronyme	FOSTINE 01 (FOcal Secured Targeted Induced Energy)
Investigator coordinator	Nicolas BARRY DELONGCHAMPS<br>Service d'urologie<br>Hôpital Cochin<br>Tél 01 58 41 27 64<br>Courriel : nicolas.barry-delongchamps@aphp.fr
Promotor	Assistance Publique – Hôpitaux de Paris
Scientific rational	Decrease the risk of tumor progression and need for radical treatment
Primary objective and endpoint	To assess the feasibility of focal transrectal microwave treatment of an index prostate tumor identified by MRI, in patients with prostate cancer at low risk or at low intermediate risk of progression.<br><br>The primary endpoint is complete necrosis of the target volume on multiparametric MRI of the prostate performed 7 days after treatment.
Secondary objectives and endpoints	1 / Evaluate the minimum and maximum necrosis margins outside the index tumor, in the three spatial planes, by multiparametric MRI of the prostate carried out 7 days after treatment.<br><br>2 / Evaluate sexual tolerance after treatment, by self-administered questionnaires IIEF (erectile function) and MSHQ- Ej (ejaculatory function), carried out on D7, 2 and 6 months.<br><br>3 / Evaluate urinary tolerance after treatment with the IPSS and IPSS-QDV self-questionnaire, the cytobacteriological examination of the urine, the maximum urine output, the Prostate Specific Antigen (PSA), and the introduction of any additional treatment aimed at urinary, at D7, 2 and 6 months.<br><br>4 / Evaluate the oncological efficacy after treatment by multiparametric MRI of the prostate and targeted biopsies

	at the level of the treated volume, 6 months after the operation.
Experimental design	Single-center study
Population concerned	Adult men with low risk or low intermediate risk of progression to prostate cancer
Inclusion criteria	- Patient aged 45 to 76 ;<br>- Life expectancy at inclusion of more than 10 years ;<br>- Diagnosis of prostate cancer confirmed by transrectal biopsies of the prostate ;<br>- Classification in the group at low risk or low intermediate risk of progression of D ' Amico , defined by :<br>* a clinical stage T1c or T2a on rectal examination<br>* a maximum biopsy Gleason score of 3 + 4, including on targeted biopsies corresponding to less than 50% grade 4 in the tumor<br>* a serum Prostate Specific Antigen level <15 ng / mL<br>- Demonstration of a tumor area by multiparametric MRI of the prostate, confirmed by targeted transrectal biopsies by MRI-ultrasound image fusion ;<br>- Patient accepting to be included in an active surveillance protocol at the end of the study, in accordance with the recommendations of good practice ;<br>- Patient affiliated to a social security scheme ;<br>- Free, informed and written consent, dated and signed by the patient and the investigator, at the latest on the day of inclusion and before any examination required by the study.
Non-inclusion criteria	- History of prostate surgery ;<br>- History of radiotherapy or pelvic trauma ;<br>- History of documented acute or chronic prostatitis<br>- History of allergy or non-tolerance to Gadolinium salts used in MRI ;<br>- Patient with a contraindication to performing an MRI<br>- Severe urinary symptoms linked to benign prostatic hyperplasia, and defined by an IPSS score> 18 ;<br>- tumor with MRI signs of extra-capsular extension or invasion of the seminal vesicles.<br>- Demonstration of a tumor invasion having a length greater than 3 mm on the systematized biopsies carried out outside the tumor zone visible on multiparametric MRI of the prostate.<br>- Demonstration of a major tumor axis greater than 20 mm on multiparametric MRI of the prostate ;<br>- Demonstration of a distance of less than 5 mm between the tumor circumference and the rectum.<br>- Person placed under judicial protection.
Device under investigation	*Initial phase of development of intra-prostatic microwave focal transrectal treatment under general anesthesia*

Reference treatment	*Radical prostatectomy*
Other medical acts added by research	Multiparametric MRI of the prostate on D7 after the operation
Risks added by research	*Risk D*
Surgical procedure	Eligible patients will be included. The intervention will be organized at the time of the inclusion visit and scheduled within 1 month. The intervention will consist of focal, transrectal, microwave treatment of the index prostate tumor identified by MRI. It will be performed in the operating room, under general anesthesia. Patients will be allowed to return to their homes the day after the operation. A multiparametric MRI of the prostate will be performed 7 days after the operation, then a control visit will take place 2 months and 6 months after the operation. At 6 months, a multiparametric MRI of the prostate will be carried out followed by targeted prostate biopsies in the treated area.
Number of subjects	10
Number of centers	One center
Research duration	duration of inclusion : 12 months<br>duration of participation (treatment + follow-up) : 7 months maximum<br>total duration : 19 months
Number of planned inclusions per center and per month	One inclusion per month
Statistical analysis	To describe the effectiveness of the treatment : the number and percentage of patients with complete necrosis of the target volume 7 days after treatment will be given. The 95% confidence interval (one-sided) will be calculated using the exact Clopper -Pearson method.
Source of funding	*KOELIS Company*
Data and Safety Monitoring Board	Yes

2 SCIENTIFIC JUSTIFICATION OF THE RESEARCH

Screening for prostate cancer has allowed the earlier detection of localized tumors of small volume and low aggressiveness [1]. The management of these forms at low risk or low intermediate risk of progression is now evolving towards a de-escalation of therapy. Radical treatments for prostate cancer (surgery or external radiotherapy), because of their possible significant impact on quality of life, are in fact not suitable for these selected patients. Radical prostatectomy, in particular, is fraught with significant urinary (incontinence) and sexual side effects [2]. Brachytherapy, for its part, causes urinary morbidity, which is transient in most cases, but which can bother the patient for several months [2]. More rarely, we can observe radiation urethritis , a feared complication and difficult to treat, affecting 1 to 2% of patients [3]. Active surveillance has therefore been proposed for these patients, with the prospect of offering radical treatment in the event of progression.

However, an innovative alternative currently under evaluation is to treat the most aggressive tumor focus in a focal way, known as the index tumor, in order to reduce the risk of progression, by avoiding radical treatment. Performing focal treatment involves locating the tumor and focusing the therapeutic agent on the area to be treated with great precision.

We propose to take advantage of our expertise in focal treatment [4], tumor localization by MRI [5, 6] and targeted biopsies [7-9] with image fusion to assess the feasibility of a focal treatment strictly limited to the tumor (or " ultra-focal ") area of cancer by microwave energy delivered by the transrectal route. Patients carrying a single visible tumor on multiparametric MRI would be included in this protocol, an active monitoring is then implemented to detect and treat early recurrence in the treated area or 2 nd prostatic cancer metachronous .

2.1 Research hypotheses

We hypothesize the feasibility and safety of focal treatment of the index tumor, carried out by microwaves delivered transrectally through a needle, in patients with prostate cancer at risk. low or low intermediate risk of progression and whose index tumor is identifiable on multiparametric MRI of the prostate.

2.2 Description of knowledge relating to the pathology concerned

Epidemiological data and screening for prostate cancer

The incidence of prostate cancer has been steadily increasing in France since the early 1990s [10]. In the United States, there has been a "peak" in incidence during the same period, followed by an increase in incidence up to the present day [11]. The hypothesis of a link between the increased incidence and the increasing practice of PSA dosing has been suggested, although it has not been clearly demonstrated. The increase in the prevalence of cancer, itself dependent on environmental factors, is also a possible hypothesis [12].

For several years now, the French association of urology, as well as European and American learned societies, have recommended individual screening for prostate cancer once a year [13]. This screening is intended for men aged 50 to 75, that is to say those with a life expectancy of more than 10 years. It is recommended to start this screening at the age of 45 years in case of family risk (family history of prostate cancer) or ethnic (populations of African

or Caribbean origin). Screening is based on two tools, digital rectal examination (DRE) and total PSA assay. This screening is nevertheless the subject of much controversy, because it involves being supplemented by a series of 12 systematic prostate biopsies which now carry a risk of “over-detection” and therefore of “over-treatment” of so-called cancers. at low risk or low intermediate risk of progression.

Prognostic classification and recommended treatments

It is recommended to distinguish three prognostic groups according to the so-called D'AMICO classification which applies to all therapeutic modalities [14]. The group with a good prognosis, said to be at low risk of progression [clinical stage T1c or T2a, Gleason score 3 + 3, PSA <10ng / ml] can be managed by active surveillance, external or interstitial radiotherapy or resection surgery [15] .] . The low intermediate risk group [clinical stage T2a, Gleason score 3 + 4, PSA <15ng / mL] cannot be managed by active surveillance, but can benefit from all active therapies, in particular the less invasive of between them that is interstitial radiation therapy. While they are also effective, with a specific risk of death of less than 1% at 15 years, none is completely devoid of consequences on the quality of voiding and sexual life [16].

For its part, active surveillance, which requires a clinical reassessment (rectal examination), biological (PSA) and above all regular biopsy, is a source of anxiety [17] and involves a minimal but real risk of infection associated with the biopsy (hospitalization for complication of the order of 3.8% within 60 days after the biopsy). Thus, more than a third of the patients included in the monitoring protocols request to be discharged in the medium term [18] and seek radical treatment.

Development of multiparametric prostate MRI and guidance tools

2.2.1.1 **Multiparametric MRI**

Multiparametric MRI of the prostate, which combines a traditional T2-weighted morphological sequence, a diffusion sequence and a dynamic sequence after intravenous injection of Gadolinium, has shown its high sensitivity, varying from 71 to 86%, to detect and characterize Tumor foci of more than 0.2 cm3 [19-24], in particular in patients with cancer at low risk or low intermediate risk of D ' Amico [8, 9, 22]. The combination of morphological and functional sequences, dominated by diffusion, has indeed enhanced the diagnostic reliability of MRI, as well as its value in detecting the presence of an aggressive tumor.

Currently, multiparametric MRI is performed as part of routine care both to assess the locoregional extension of prostate cancer (search for extra-capsular extension, invasion of seminal vesicles, pelvic lymph node involvement) and to search for tumor target (s). It is recommended by learned societies [13] and performed before biopsies in expert centers, which makes it possible to guide samples from areas suspected of cancer.

The examination procedures are as follows :

The choice of the magnet (1.5 or 3 Tesla) and of the antenna (pelvic alone or combined with an endorectal antenna) depends on the habits of each center. Recommendations for a minimum equipment level have been reported (ESUR, PIRADS v2)

Sequence in T2:
T2 acquisition is most often carried out in 2D mode with, depending on the centers, a pelvic antenna combined with an endorectal antenna or a pelvic antenna alone, provided it has at least 16 channels. Volume 3D acquisition can also be performed.

Broadcast sequence:
An acquisition sequence is performed in echo-planar (EPI), with a section thickness of 2.5-3.5 mm and three values of b (50-500-1000 s / mm²). To superimpose the information provided by each sequence, the sections have the same thickness and centering

Dynamic sequence after Gadolinium injection (dynamic MRI):
Intravenous injection of Gadolinium is given as a bolus at a dose of 0.1 mmol / kg, followed by a bolus of 15 ml of physiological serum. The acquisitions are repeated for 2 to 5 minutes with a frequency (temporal resolution) of less than 15 seconds. They aim to detect an early enhancement of a hypointense visible in T2 or in diffusion (Table 2). The cuts have the same centering and the same thickness as the cuts in the T2-weighted sequence.

The definition of a suspect area is based on a probability score assigned to each region of interest (scale of 1 to 5) on the basis of the recommendations of the European Society of Uro-Radiology and the American College of Radiology (table 1) [23, 24]. An overall score on a scale of 1 to 5 is then assigned which takes into account a different so-called dominant sequence for the peripheral zone and the transition zone. For lesions in the peripheral zone, the dominant sequence is the diffusion sequence which therefore gives the overall score as a rule for determining the level of probability of malignancy. For lesions in the transition zone, the overall score is as a rule that of the T2 sequence, the dominant sequence in this zone.

Table 1: T2 imaging, diffusion and perfusion imaging (dynamic) score recommended by the European Society of Urogenital Radiology (ESUR) and the American College of Radiology (ACR)

T2 spin echo weighted sequence
Peripheral zone (ZP)
1. homogeneous hypersignal .
2. hyposignals triangular , linear
3. poorly limited geographical ranges, not classifiable in categories 2 or 4-5.
4. hyposignal with mass effect of appearance confined to the prostate, with a long axis ≤15mm.
5.similar to 4, but with extra-capsular or major axis extension> 15mm

Transition zone (ZT)
1. Heterogeneous appearance of the ZT without detectable focal anomaly.
2. Encapsulated nodule, in homogeneous or heterogeneous hypointense .
3. Beach hypointense heterogeneous and poorly defined not classifiable in categories 4 or 5.
4. Homogeneous hypointense area , poorly limited, anterior and often lenticular in shape, with a long axis ≤15mm
5. Same aspect as 4, but with involvement of the anterior fibro- muscular stroma or the lateral horn of the ZP, or with a major axis> 15mm.

Diffusion-weighted sequence (ZP and ZT)
1. homogeneous appearance without a drop in the Apparent Diffusion Coefficient (ADC) compared to normal tissue. No increase in signal strength on images obtained with a high diffusion gradient (b-value ≥1400)

2. Diffuse hypointense on ADC mapping with diffuse increase in signal intensity on images obtained at b ≥1400. No focal abnormality, unless triangular or linear in shape
3. beach hypointense moderate on the ADC map and isointense or hyperintense moderate on images obtained b ≥1400c
4. hypointense focal large ≤15mm axis on the ADC map and hyperintense on images obtained b ≥1400,
5. hypointense focal long axis> 15 mm on the ADC map and hyperintense on images obtained b ≥1400,

Contrast imaging after Gadolinium injection. (ZP)

1. focal, early or contemporary enhancement corresponding to a hypointense visible on the T2-weighted or diffusion-weighted sequence. *

0. no early enhancement or diffuse enhancement not corresponding to a focal anomaly on the T2-weighted and / or diffusion-weighted sequence, or focal enhancement in a hyperplastic-looking nodule on the T2-weighted sequence.

Table 2: PIRADS score applied to the peripheral zone. *Score 1 : significant injury very unlikely Score 2 : significant injury unlikely*
Score 3 : significant lesion that cannot be ruled out Score 4 : probable significant lesion
Score 5 : significant injury very likely

Diffusion	T2	Infusion	global
Score 1	indifferent*	indifferent	Score 1
Score 2	indifferent	indifferent	Score 2
Score 3	indifferent	Negative (0)<br>Positive (1)	Score 3<br>Score 4
Score 4	indifferent	indifferent	Score 4
Score 5	indifferent	indifferent	Score 5

* : whatever the score (from 1 to 5)

Table 3: PIRADS score applied to the transition zone
Score *1 : significant injury very unlikely Score 2 : significant injury unlikely*
Score 3 : significant lesion that cannot be ruled out Score 4 : probable significant lesion
Score 5 : significant injury very likely

T2	Perfusion	Diffusion	Global PIRADS
Score 1	indifferent	indifferent	Score 1
Score 2	indifferent	indifferent	Score 2
Score 3	indifferent	Score 3<br>Score 4	Score 3<br>Score 4
Score 4	indifferent	indifferent	Score 4
Score 5	indifferent	indifferent	Score 5

2.2.1.2 **Guidance tools : the Koelis Trinity system**

The development of image fusion techniques between MRI and ultrasound makes it possible to target suspicious areas visible on multiparametric MRI using ultrasound. Several systems have been marketed, including the Trinity® system (Koelis , Grenoble, France), which has been clinically evaluated and has demonstrated its reliability to guide a prostate biopsy [7-9, 25-28.] With a average accuracy of about 3mm to hit a target of 0.5cc. [29].

Currently, this CE certified system is used as part of routine care to perform targeted prostate biopsies. Its use slightly increases the duration of the samples without significantly modifying the classic biopsy protocol. The technique learning curve is accessible to any practitioner trained in ultrasound-guided prostate biopsies. The image fusion procedure is detailed in the user manual provided in the appendix.

In summary, multiparametric MRI of the prostate, as well as the performance of targeted biopsies guided by MRI-ultrasound image fusion, currently make it possible to improve the characterization of prostate cancer, by specifying with better reliability the score of Gleason of cancer, its volume and location.

2.3 Summary of preclinical experiments and clinical trials concerned

Focal treatment remains currently under evaluation, its implementation in fact posing three questions that are still imperfectly resolved: how to precisely define the area to be treated, how to focus the therapeutic agent on this target and finally how to safely monitor the patient. rest of the gland after treatment, given the possible existence of undetected infra-clinical tumor foci [30].

To date, several modes of tissue ablation have been tested, within the framework of zonal prostate or hemi-ablation treatments. Most of them use ultrasound guidance. Cryotherapy [31, 32] via the tranperineal route and focused ultrasound via the external transabdominal route [33-35] have been used in a few expert centers. The short published series have yielded interesting results [30, 34]. More recently, dynamic phototherapy of the prostate by the tranperineal route , using a photosensitizer (Tookad) injected intravenously, has been tested in patients with prostate cancer at low risk of progression, defined by the results of a classic protocol of 10-12 systematic biopsies. Several phase II studies have suggested the feasibility and good urinary and sexual tolerance of this approach [36, 37]. A multicenter phase III international study, comparing this technique to active surveillance, in which we participated, has just been completed. The results are in the process of being published.

However, the absence of image registration between ultrasound and MRI during application of the treatment introduces worrying uncertainty on the precision of the guidance. In addition, the prostate is an extremely mobile organ and the simple insertion of an ultrasound probe and even more of a needle causes movements of up to a centimeter, leading to major inaccuracy in targeting. In fact, the imperfect delineation of the target area means that the focal treatments currently carried out consist of an ablation of the entire right or left part of the gland (hemi-ablation).
To increase the precision of the ablative procedure, a few teams have proposed performing focal treatments under real-time MRI guidance via the tranperineal [38] or transrectal route [39], with good short-term functional and oncological results. Nevertheless, the high precision of these techniques is obtained to the detriment of an examination duration of several hours, limiting the distribution of these treatments.

We propose to overcome these obstacles by using the commercial image fusion system TRINITY (Koelis , Grenoble, France).

1) The definition of the target area will be based on the results of multiparametric MRI imaging (T2, Diffusion, Contrast injection), the MRI images then being transferred to the TRINITY system for biopsies. Regions of interest will then be delimited on the MRI and registered with the echographic volume acquired with a 3D probe, this system will make it possible, during biopsies, to archive the spatial location of the needle paths in relation to the region of interest, and therefore of the index tumor to be treated.

2) In addition, the precision of the system to locate a needle in the prostate volume [29] will be used to place in the heart of the target by transrectal route, the applicator of the microwave source. This applicator is in the form of an 18 gauge needle, the diameter of which is identical to that of the needles used for performing prostate biopsies as part of routine care.

2.4 Description of the population to be studied and justification for its choice

The population included will be made up of men with a life expectancy of more than 10 years, and with localized prostate cancer at low risk or low intermediate risk of progression according to the definition of D ' Amico (detailed in the chapter 2.2.2). In addition, only tumors without infra-clinical extra-prostatic extension and on multiparametric MRI will be included.

These eligibility criteria define this population that can derive the optimal benefit from focal treatment, as considered by an international consensus conference [40].

2.5 Name and description of the medical device

The treatment under study is ***focal microwave ablation*** . This treatment will be delivered by an 18 gauge applicator (TATOpro ® 18G from Biomedical SRL) introduced under ultrasound guidance via the transrectal route, and connected to a generator (TATO ® from Biomedical). The technical characteristics and user guide of the applicator and generator are provided in the appendix.

The guidance of this treatment needle will be achieved by ultrasound-MRI image fusion using the TRINITY® system (Koelis , Grenoble, France), the characteristics of which are detailed in the appendix.

During the treatment, the microwave applicator will be maintained, integrally with the ultrasound probe, by a mechanical arm, the characteristics of which are detailed in chapter 7. This arm makes it possible to maintain the position and orientation of the ultrasound. applicator when the generator emits the waves.

2.6 Description and justification of the methods of use of the medical device

The administration of microwaves is detailed in chapter 7. The duration of administration and the power delivered by the generator can be adjusted. These parameters will be adapted to the target volume to be treated, according to a pre-established chart provided in the appendix.

2.7 Summary of foreseeable and known benefits and risks for those undergoing research

2.7.1 Foreseeable benefits

The predictable individual benefits relate to the focal treatment of the index tumor of prostate cancer. As explained in chapter 2.2.2., The treatments recommended in patients with prostate cancer at low risk of progression range from active surveillance to total prostatectomy.

If it is positioned in relation to support the less aggressive (active surveillance), the benefit is oncological. The study of cohorts of patients under active surveillance suggests that about a third of patients will have cancer progression, most often during the first 2-3 years [41]. Furthermore, it is generally accepted that the index tumor is the cause of cancer progression [13]. Focally treating the index tumor should therefore eliminate the risk of tumor progression incurred with active surveillance, and reduce the risk of having to resort to radical prostatectomy and the morbidity to which it exposes.

If we position ourselves in relation to the most aggressive treatment (total prostatectomy), the expected benefit is a major reduction in the morbidity induced. In fact, total prostatectomy is the cause of urinary incontinence in one third of cases and erectile dysfunction in more than half of patients [13]. Treat focally the index tumor preserves the rest of the gland, neurovascular strips and striated urethral sphincter.

2.7.2 Foreseeable risks

The foreseeable risks of research are linked on the one hand to the transrectal approach, and on the other hand to the use of microwaves. There is no risk when using the TRINITY guidance and navigation module.

2.7.2.1 Urinary and sexual functional risk

The foreseeable risks of a focal ablative treatment of the prostate are linked to the diffusion of energy outside the gland, in the rectum, in the urethra, as well as in the vascular nerve strips. The published series of focal treatment by cryotherapy, focused ultrasound, or dynamic phototherapy have suggested the good tolerance of these treatments, even though they were not treatments limited to the index tumor but more voluminous tissue ablations, encompassing the index tumor and going as far as hemi-ablation [34, 36, 37].

Feijo et al [34] reported the early functional results of focused ultrasound hemiablation in 71 patients. At 3 months, all the patients were continent, and erectile function was maintained in 11 of the 21 patients with erectile function considered to be normal preoperatively . The authors reported the occurrence of grade 3 complications in the Clavien classification in 2.8% of patients.

A combined analysis of the results of phase II studies evaluating prostatic hemi-ablation by dynamic phototherapy after intravenous injection of Tookad is available : Azzouzi et al [36, 37]

reported functional urinary and sexual results at 6 months in 117 patients. . Urinary scores remained stable over time. The erectile score (IIEF5) decreased slightly, but not significantly.

To our knowledge, only one series on focal treatment limited to the index tumor has been published : Lepor et al [39] reported in 25 patients the functional results at 3 months of a focal treatment of the index tumor by interstitial laser delivered. transrectally under real-time MRI guidance. Urinary and sexual functional scores were unchanged from the preoperative period.

In the proposed research , we predict functional suites, linked to energy diffusion, comparable to those reported by Lepor et al [39], and therefore a non-significant risk. According to the microwave treatment charts, the energy diffusion is reproducible, the comet effect negligible, and the tissue necrosis zone generated is therefore predictable at a given treatment power and duration. The thermal effect is therefore at most equivalent to that generated by an interstitial laser such as that used in the study by Lepor et al [39]. Finally, real-time control of the position of the applicator will allow precise measurement of the distance of the treatment area from the rectum.

2.7.2.2 **Infectious risk**

In the proposed research, the risk of infection is linked to the transrectal approach. This risk is widely evaluated in the literature in patients subjected to prostate biopsies by the transrectal route, more particularly in patients without cancer and in the event of repeated biopsy during surveillance. A Canadian registry study was performed in 37,190 patients who had prostate biopsies. This study showed a rate of hospitalization for complication within 30 days after the act of 1.9% [42]. The rate of severe infectious complications and acute retention of urine reported after prostate biopsies performed by the transrectal route is less than 1%. It is limited by the prescription of prophylactic antibiotic therapy prior to the procedure and which can continue between 24 and 72 hours [13, 43].

**Complications**	**Frequency (%)**
Hemospermia	37.4
Urethrorrhagia > 1 day	14.5
Fever	0.8
Sepsis	0.3
Rectorragie	2.2
Acute urine retention	0.2
Prostatitis	1
Epididymitis	0.7

Table 1 : Complications reported after systematized ultrasound-guided prostate biopsies via the transrectal route [15]

Lepor et al. [39], do not report a case of infection after focal ablation by the transrectal route under MRI guidance.

In the proposed research , we therefore estimate that the risk associated with the transrectal approach is minimal. Compared to the transperineal route , the trans-rectal route is considered

to be less invasive and painful. The foreseeable risk of using the trans-rectal route in the proposed research is therefore identical to that of prostate biopsies, used in routine care for more than 30 years. The microwave applicator has in fact the same diameter as the needle used for prostate biopsies (18 gauges). The mucosal lesion generated on the puncture path will therefore be of the same order.

2.7.2.3 **Benefit / Risk Report**

Given the expected benefits and the foreseeable risks, the treatment under study seems to be halfway between active surveillance and radical prostatectomy, these two forms of treatment being accepted in clinical practice in the population subjected to the procedure. proposed research.

3 GOALS

3.1 Main objective

To assess the feasibility of focal transrectal ablative microwave treatment of an index prostate tumor identified by multiparametric MRI, in patients with prostate cancer at low risk of progression according to the Amico classification .

3.2 Secondary objectives

1 / Evaluate the degree of extension of focal transrectal microwave treatment of the prostate index tumor ;

2 / Evaluate the sexual tolerance of focal transrectal microwave treatment of the prostate index tumor ;

3 / Evaluate the urinary tolerance of focal transrectal microwave treatment of the prostate index tumor ;

4 / Evaluate the carcinological evolution at 6 months after focal transrectal microwave treatment of the prostate index tumor.

4 RESEARCH DESIGN

4.1 Precise statement of primary and secondary endpoints

Primary endpoint

The primary endpoint is complete necrosis of the target volume defined on MRI before treatment. This necrosis will be evaluated on a multiparametric MRI performed 7 days after treatment.

Secondary endpoints

1 / The degree of extension of the treatment will be defined on the MRI on D7 after treatment by the length (in millimeters) of the minimum and maximum necrosis margins outside the target volume, in the three planes of space
2 / Sexual tolerance will be assessed by the IIEF (erectile function) and MSHQ- Ej (ejaculatory function) self-questionnaires (provided in the appendix), carried out on D7, 2 and 6 months.

3 / Urinary tolerance will be assessed by the IPSS and IPSS-QDV self-questionnaires (provided in the appendix), the cytobacteriological examination of the urine, the maximum urine output, the Prostate Specific Antigen (PSA), and the introduction of any treatment additional for urinary purposes, on D7, 2 and 6 months.

4 / The carcinological evolution will be evaluated by multiparametric MRI of the prostate and targeted biopsies at the level of the treated volume, 6 months after the operation.

4.2 Description of the research methodology

Experimental plan

This research is an interventional, open and non-comparative study

Number of participating centers

The research is monocentric . The urology department of Cochin Hospital will participate in the research and be responsible for recruiting patients.

Identification of subjects

As part of this research, the subjects will be identified as follows :
n ° center (3 digit positions) - No. order of selection of the person in the center (4-digit) - Initial name - name initial
This reference is unique and will be kept for the duration of the research.

5 RESEARCH PROGRESS

Before any examination or act related to the research, the investigator obtains the free, informed and written consent of the person who is performing the research or of his legal representative, if applicable.

5.1 Selection visit : V-1

The selection visit takes place between 2 *months* and at the latest *3 days* before the inclusion visit.
This visit takes place in consultation.

During this visit, the investigator:
- Perform a clinical examination including a rectal examination, and specifying the presence or not of a suspicious prostate nodule.

- Check the PSA level, measured as part of routine care and dating less than 3 months.
- Check that multiparametric MRI of the prostate, less than 2 months old and carried out as part of routine care, has located a suspicious target corresponding to the tumor, specifying its exact location, including its relationship to the distal sphincter and the prostatic urethra as well as its major axis. The absence of extra-capsular extension and to the seminal vesicles of the tumor, as well as the absence of suspicious pelvic lymphadenopathy.
- Check the results of targeted prostate biopsies performed as part of routine care : these biopsies must have been directed to the suspicious area described in multiparametric MRI of the prostate by MRI-ultrasound image fusion using the Koelis system Grenoble, France).
- Check the absence of significant tumor (UCL definition) on systematic biopsies of normal looking sextants on MRI
- Check the sterility of urine on the cytobacteriological examination carried out a few days before as part of routine care.
- Check the eligibility criteria and then inform the patient of the research.
- Provide the patient with the research information sheet, and inform the patient of the 3-day reflection period before the possibility of inclusion in the study. This deadline must be respected before signing the informed consent sheet.

**Persons whose consent is sought**	**Who informs and collects the consent of the person**	**When is the person informed**	**When the person's consent is obtained**
The person who is suitable for research	• *the investigator (urologist)*<br>• *his representative*	*Selection visit and inclusion visit*	*After a 3-day cooling-off period*

5.2 Inclusion visit V0

The inclusion visit takes place at the earliest 3 days after the selection visit, and at the latest 2 months after.
This visit takes place in consultation.

During this visit, the investigator:
- Check the inclusion and non-inclusion criteria ;
- Provide the patient with additional information regarding the research ;
- Have the patient sign the informed consent form ;
- Perform a clinical examination including at least a digital rectal examination;
- Perform a flow measurement with measurement of the maximum flow ;
- Will have the patient complete the IIEF, MSHQ- Ej , IPSS and IPSS- QDV self-questionnaires ;
- Organize the intervention visit, which must be scheduled within 1 month, during hospitalization in the urology department of Cochin hospital :
    - Preoperative blood test, as performed as part of routine care
    - Anesthesia consultation

5.3 Follow-up visits

Intervention visit : V1

The intervention visit takes place within 1 month after the inclusion visit. The operation is performed during hospitalization in the urology department of Cochin hospital.
During this visit, the study treatment will be carried out, as described in chapter 7 of the protocol.

Visit the 7 th day post-intervention : V2

This visit will be carried out in consultation, by a urologist, 7 days after the intervention visit. An interval of 5 additional days will be tolerated for the realization of this visit.

During this visit, the doctor :

Perform a clinical examination, including at least :

Temperature measurement ;

A digital rectal examination ;

Check for the absence of any adverse event ;

Check the urine for sterility on the cytobacteriological examination of the urine carried out three days earlier ;

Perform a flow measurement with measurement of the maximum flow ;

Inform the patient of the result of the multiparametric MRI of the prostate carried out seven days after the intervention visit;

Retrieve the IIEF, MSHQ- Ej , IPSS and IPSS- QDV self-questionnaires previously completed by the patient, and provide them with the self-questionnaires to be completed for the next follow-up visit.

Inform the patient of the continuation of the management, and of the next follow-up visit

2-month visit : V3

This visit will be carried out in consultation, by a urologist, 2 months after the intervention visit. An interval of more or less 15 days will be tolerated for the realization of this visit.

During this visit, the doctor :

Perform a clinical examination, including at least : a digital rectal examination ;

Check for the absence of any adverse event ;

Perform a flow measurement with measurement of the maximum flow ;

Take a blood sample to measure the level of Prostate Specific Antigen (PSA) ;

Retrieve the IIEF, MSHQ- Ej , IPSS and IPSS- QDV self-questionnaires previously completed by the patient, and provide them with the self-questionnaires to be completed for the next follow-up visit.

Inform the patient of the continuation of the management, and of the next follow-up visit

At the end of the visit, the investigator will schedule the performance of a multiparametric MRI of the prostate and targeted biopsies at 6 months, as planned as part of routine care in the event of active surveillance.

5.4 End of research visit : V4

This visit will be carried out in consultation, by a urologist, 6 months after the intervention visit. An interval of more or less 15 days will be tolerated for the realization of this visit.

During this visit, the doctor :

Perform a clinical examination, including at least :

A digital rectal examination ;

Check for the absence of any adverse event ;

Perform a flow measurement with measurement of the maximum flow ;

Take a blood sample to measure the level of Prostate Specific Antigen (PSA) ;

Inform the patient of the result of the multiparametric MRI of the prostate and the targeted biopsies taken a few days previously.

Retrieve the IIEF, MSHQ- Ej , IPSS and IPSS- QDV self-questionnaires previously completed by the patient.

Inform the patient of the end of the research and explain the rest of the treatment.

The methods of medical care for people planned at the end of the research are those relating to the active surveillance of prostate cancer at low risk or low intermediate risk of progression, as specified in the recommendations for good practice [Salomon].

5.5 Expected duration of patients participation, description of the chronology and duration of the research.

Maximum time between selection and inclusion	2 months
Length of the inclusion period	12 months
Duration of participation of subjects, including :	7 months maximum
• Duration of treatment :	2 to 20 minutes
• Duration of follow-up :	6 months +/- 15 days
Total search time :	19 months

5.6 Summary table of the research chronology

	V-1	*V0*	*V1*	*V2*	*V3*	*V4*
Actions	*D-60 - D-3 days (selection)*	*D0 (**inclusion)***	*D0 +/- 1 month (intervention)*	7- day follow-up	2-month follow-up	6-month follow-up (end of study)
Information sheet	*X*					
Informed consent		*X*				
Check eligibility criteria	*X*	*X*				
Rectal touch	*X*	*X*		*X*	*X*	*X*
PSA	*X*				*X*	*X*
Flowmetry		*X*		*X*	*X*	*X*
Self-questionnaires		*X*		*X*	*X*	*X*

Multiparametric MRI of the prostate	*X*			*X + 2 days if necessary*		*X*
Targeted prostate biopsies	*X*					*X*
*Preoperative assessment **		*X*				
Dispensing of treatment			*X*			
Adverse events			*X*	*X*	*X*	*X*

* *Preoperative* assessment *:* blood assessment comprising a blood count, a hemostasis assessment, a double determination of blood group and Rhesus, a blood ionogram, a serum creatinine assay ; urine test including cytobacteriological examination of the urine.

5.7 Distinction between research and standard of care

TABLE : Distinction between acts related to " standard of care " and acts added by " research "

Acts, procedures and treatments carried out in the context of research	Acts, procedures and treatments related to care	Acts, procedures and treatments added by research
**Treatments**		*Microwave transrectal treatment*
**Consultations**	*Consultation at 3 and 6 months Including a digital rectal examination and self-questionnaires*	*Additional consultations V0, V2, V3, (at inclusion, at D7 and 2 months)*
**Blood test**	*PSA at 3 and 6 months*	
**MRI of the prostate**	*Multiparametric MRI at inclusion and at 6 months*	*Multiparametric MRI on D7*
**Biopsies**	*Baseline and 6 month prostate biopsies*	

5.8 Shutdown rules

Criteria and modalities for premature termination of research treatment

5.8.1.1 Different situations

- Temporary discontinuation of treatment, the investigator must document the reason for the discontinuation and its resumption in the subject's source file and the CRF
- Premature discontinuation of treatment, but the subject remains in the research, until the end of his participation, the investigator must document the reason
- Premature discontinuation of treatment and cessation of participation in research.

The investigator should :

- Document the reason (s)
- Collect the evaluation criteria when stopping participation in the research, if the subject agrees
- Continue to monitor the subject, especially in the event of a serious adverse reaction

5.8.1.2 Criteria and modalities for premature termination of research

- Any subject can stop participating in the research at any time and for any reason.
- The investigator may temporarily or permanently discontinue a subject's participation in research for any reason having an impact on his safety or which would serve the subject's best interests.
  - Subject lost to sight : we do not know what has become of the subject. The investigator must make every effort to reconnect with the subject (and trace it in the source file) in order to know at least if the subject is alive or dead.

In the event of premature termination of the research of a subject, or of withdrawal of consent, the data concerning him may be used in the absence of opposition from the latter when signing the consent.

The observation book must list the different reasons for stopping participation in the research:

- ☐ Inefficiency
- ☐ Adverse reaction
- ☐ Other medical problem
- ☐ Subject's personal reason
- ☐ Explicit withdrawal of consent

Follow-up of subjects following premature discontinuation of treatment

Stopping a subject's participation will not change their usual management of their disease.
The occurrence of a serious adverse event will be notified by the investigator to the sponsor and will be followed up for 12 months following premature discontinuation of treatment (to be adapted according to research). In the event of premature discontinuation of treatment following the occurrence of a serious adverse event, a notification of a serious adverse event will be sent by fax (01 44 84 17 99) to the sponsor. The serious side effect will be monitored until it resolves.

Stop some or all of the search

The AP-HP promoter or the Competent Authority (ANSM) may prematurely interrupt all or part of the research, temporarily or permanently, in the following situations:

- in first, in case of unexpected serious adverse reactions (SUSARs) requiring a reassessment of the risk / benefit of research.
- of the same, unforeseen events, new product information, upon which the objectives of the research or clinical program are unlikely to be met, may lead the AP-HP promoter or the Competent Authority (ANSM) to interrupt prematurely looking.
- the promoter AP-HP reserves the right to definitively suspend the inclusions, at any time, if it turns out that the inclusion objectives have not been achieved.

In all cases of stopping a research, the subjects included in the research must be followed until the end of their participation, as foreseen by the protocol.

In the event of premature termination of the research, the decision and the justification are sent by the promoter AP-HP within 15 days to the Competent Authority (ANSM) and to the CPP.

6 ELIGIBILITY CRITERIA

6.1 Inclusion criteria

- Patient aged 45 to 76 yo;
- Life expectancy at inclusion of more than 10 years ;
- Diagnosis of prostate cancer confirmed by transrectal biopsies of the prostate ;
- Classification in the group at low risk or low intermediate risk of progression of D ' Amico , defined by :
* a clinical stage T1c or T2a on rectal examination
* a maximum biopsy Gleason score of 3 + 4, including on targeted biopsies corresponding to less than 50% grade 4 in the tumor
* a serum Prostate Specific Antigen level <15 ng / mL
- Demonstration of a tumor area by multiparametric MRI of the prostate, confirmed by targeted transrectal biopsies by MRI-ultrasound image fusion ;
- Patient accepting to be included in an active surveillance protocol at the end of the study, in accordance with the recommendations of good practice ;
- Patient affiliated to a social security system ;
- Free, informed and written consent, dated and signed by the patient and the investigator, at the latest on the day of inclusion and before any examination required by the study.

6.2 Non-inclusion criteria

- History of prostate surgery ;
- History of radiotherapy or pelvic trauma ;
- History of documented acute or chronic prostatitis
- History of allergy or non-tolerance to Gadolinium salts used in MRI;
- Patient with a contraindication to performing an MRI
- Severe urinary symptoms linked to benign prostatic hyperplasia, and defined by an IPSS score> 18 ;
- tumor with MRI signs of extra-capsular extension or invasion of the seminal vesicles.
- Demonstration of a tumor invasion having a length greater than 3 mm on the systematized biopsies carried out outside the tumor zone visible on multiparametric MRI of the prostate.
- Demonstration of a major tumor axis greater than 20 mm on multiparametric MRI of the prostate ;
- Demonstration of a distance of less than 5 mm between the tumor circumference and the rectum.
- Person placed under judicial protection.

6.3 Recruitment methods

Patient recruitment will be carried out in hospital consultation, within the urology department of Cochin hospital. Any potential inclusion in the study will first be discussed and then validated during the multidisciplinary consultation meeting of the urology department.

10 patients will have to be recruited sequentially during the inclusion period, which is well below the recruiting capacity of the urology department of Cochin hospital. In fact, around 12 potentially eligible patients are recruited every month.

	Number of subjects
Total number of subjects selected	24
Number of centers	1
Inclusion period (months)	12
Number of topics to include	10
**Number of subjects / month to include**	**1**

7 MEDICAL DEVICE USED IN RESEARCH AND TREATMENTS, PROCEDURES, STRATEGIES ASSOCIATED WITH THE USE OF THE DEVICE

7.1 Description of medical devices

The treatment under study is focal microwave thermo-ablation of a prostate tumor, performed transrectally, and guided by MRI-ultrasound image fusion, under general anesthesia.

Experimented device the subject of research

The medical device under investigation is a TATO microwave thermal ablation system from the manufacturer Biomedical . The applicator used is TATOpro 18G specially adapted for the treatment of the prostate.
Reference: TATO1
Serial number: BMEM03aaxx

Intended destination of the device

The use of the device in the context of research is as provided for in the device user manual :
" The TATO thermal ablation system is used for the percutaneous coagulation, laparoscopic , and intraoperative soft tissue (liver, lung, kidney and prostate), including partial or complete removal of inoperable tumors. This is an extreme hyperthermia procedure aimed at destroying deep tumor masses through the application of electromagnetic energy via a very small needle, between 18 and 11 G, called "applicator", inserted in the patient's body until it reaches the tumor mass, usually by ultrasound guidance. "

Terms of use of the device

All the instructions can be found in the attached user manual.
• Microwave radiation from the system may be the source of electromagnetic interference with other medical devices. In such cases, it may be necessary to move the devices away.
• The applicator must be connected to the generator before it is inserted into the patient.
• Microwave energy should only be activated when the applicator is fully inserted into the target area.
• The applicator is disposable and single use.
• At the end of the procedure, the applicator can remain warm. Therefore, do not touch the applicator after use, and do not bring it near flammable materials.

Training methods for using the device

Training in the handling, storage and use of the TATO device will be carried out in the PR1 operating room at Cochin hospital, in the operating room which will be used for research, before the installation visit. A Biomedical engineer assisted by a technician will train the entire team, including Dr Nicolas Barry Delongchamps and Alexandre Schull , IBODE staff and operating theater supervisory staff (Mr Melton Momperousse , senior manager).
This training will include in particular:
Sterilization of the microwave applicator ;
The start-up of the microwave generator ;
The settings of the power and duration of emission of microwave energy ;
The procedure for urgently stopping the emission of microwave energy.

Description of other medical devices used for research purposes

The following medical devices will be used to support the microwave system that is the subject of the research:

The KOELIS imaging and guidance system, which includes :
- The TRINITY Station
- PROMAP software
- An endocavity ultrasound probe
- A reusable KOELIS instrument holder guide

- Finally, the ultrasound probe and its guide will be held by an articulated arm of FISSO during the procedure in order to avoid any movement during the emission of microwaves.

Preparation for the intervention

7.1.1.1 Anesthesia consultation

Each patient included will have an anesthesia consultation for the operation, scheduled in the urology department of Cochin hospital at least 3 days before the surgery. During this consultation, the anesthetist will check the preoperative workup prescribed during the inclusion visit (chapter 5.6) and perform an electrocardiogram. The operation will be performed under general anesthesia.

7.1.1.2 Antibiotic and rectal enema

In accordance with the recommendations of the European Association of Urology [Naber], antibiotic prophylaxis with ofloxacin should be started 2 hours before the operation and continued for 48 hours, at a dosage of 200 mg morning and evening. In the event of allergy, antibiotic prophylaxis with Trimetoprime-Sulfametoxazole (800 mg / 160 mg) will be prescribed at a dosage of two tablets morning and evening for 48 hours.

A rectal enema will be performed the day before as well as 2 hours before the operation (one dose of Microlax).

Patient setup

The patient will be installed in the supine position, and in a gynecological position.

Look first

The approach is transrectal and ultrasound guided, as for a prostate biopsy . An evacuating enema (Microlax) is first performed the day before and the morning of the procedure. Just before treatment, a rectal antiseptic enema is performed with betadine physiological serum ,

then, after perineal disinfection, the endorectal ultrasound probe is introduced under visual and ultrasound control.

Guidance methods

7.1.1.3 Instrument holder guide

As for a transrectal ultrasound-guided prostate biopsy, a puncture guide is attached to the ultrasound probe to guide the microwave applicator along the axis of the probe (appendix).

7.1.1.4 Identification, location and delimitation of the target area

The Trinity system, described in the appendix (appendix), allows MRI-ultrasound image fusion. The tumor detected and localized by multiparametric MRI of the prostate before treatment, as part of routine care, can thus be contoured and targeted during treatment.

The MRI-ultrasound image fusion is performed as follows :

At the start of the examination, an acquisition of the prostate ultrasound volume is performed with a motorized 3D probe and recorded in the Trinity system. The 3D MRI volume, previously loaded in the Trinity station, is then readjusted to the ultrasound volume. Once the images have been registered, one or more region (s) of interest are placed to cover, in the three spatial planes, the surface of the tumor located by MRI and previously confirmed by guided biopsies. The registration of the volumes makes it possible to display the contours of the tumor indifferently on the ultrasound or MRI volume.

7.1.1.5 Targeting the region of interest

Trinity is a system capable of accurately placing, under ultrasound guidance, a needle into a target located by MRI and registered in the ultrasound volume. The technologies used by this system are proven for prostate biopsies, and will be exploited here for the placement of a microwave applicator. The aim is first of all under real-time 2D ultrasound control. In order to check the position that the applicator would have in the event of insertion (virtual position), a 3D ultrasound is then performed. It allows you to visualize the virtual position of the applicator as well as the target (s) in a 3D view. Several 3D acquisitions may be necessary in order to match the virtual position of the applicator with the position of the targets, and this before the insertion of the applicator. Once the probe is correctly placed, its position is fixed using the articulated arm. The applicator can then be inserted into the prostate. The depth of the applicator into the prostate can be monitored in real time via 2D ultrasound. Its correct position in the center of the target is verified before treatment by a final acquisition of a 3D ultrasound volume.

If necessary, the procedure can be repeated until the applicator is correctly placed. When this is the case, the needle position is saved and can be viewed later. Treatment of the target area can then be started.

Microwave delivery procedures

7.1.1.6 Microwave generator

The TATO microwave generator is a solution for local tissue ablation (necrosis). The advantage of microwave ablation is that the propagation of radiation and therefore the volume of the treated area is not very sensitive to the type of tissue treated, which allows greater control of the ablation.

7.1.1.7 **Microwave applicator**
The applicators will be received by the Cochin Hospital pharmacy and given to the surgeon against a prescription.

7.1.1.8 **Energy dose and duration of treatment**
Measurements were carried out with the manufacturer of the microwave system to produce an abacus making it possible to predict the dimensions of the volume treated as a function of the treatment parameters (the power P, and the time T). Elliptical in shape, the treated volume is represented by its diameter D and its length L.
A logarithmic law relates D to the emitted energy (E = PxT), while L is a linear relation of D :

Where is a parameter related to the attenuation of microwave radiation, is the threshold energy from which the treatment is effective, and are the linear coefficients increasing D in L.
Below is an illustration of the microwave chart obtained by experiment and statistical analysis, for a power set at 10W, and for 3 treatment times : 2, 4, 8 minutes, these values being compatible with l object of the study. For these parameters the minor axis of the treatment area increases from 6mm to 14mm, the major axis from 13mm to 24mm (Figure 1). The Figure 2 illustrates a continuous representation of the minor axis D treated according to the power and time.

End of the intervention

Immediately after the end of the microwave treatment, the endo -rectal probe is withdrawn, the patient installed in strict supine position, then awakened according to the same procedures as standard care.

Postoperative care

Postoperative care is equivalent to that of a prostate biopsy as it may be performed under general anesthesia, as part of routine care.

> **Immediate postoperative** : The patient is taken to a post-interventional monitoring unit.
* It remains there for the time necessary for monitoring.
* He is then escorted by the SSPI caregiver back to his room.
* Continuity of monitoring and care is provided by the nurse and the nursing assistant in the hospital ward.
* A snack is offered to the patient two hours after the operation.
* The spontaneous resumption of urination is monitored, and the pain evaluated by visual analogue scale.

> **The day after the operation,** the patient is authorized to return home if his state of health allows it, with:
* The discharge authorization signed by the doctor
* The service phone number if needed
* Discharge orders, including the continuation of the antibiotic prophylaxis for 24 hours and level 1 analgesics (paracetamol) if necessary.

* The follow-up prostate MRI appointment on D7 and the consultation appointment for the V3 visit.

7.2 Description of non-experimental treatments

Antibioprophylaxis

Antibiotic prophylaxis with Ofloxacin , 200 mg morning and evening for 48 hours or, in the event of allergy to Ofloxacin , with Trimetoprime-Sulfametoxazole (800 mg / 160 mg) morning and evening for 48 hours, will be started 2 hours before the intervention.

Rectal enema

A rectal enema by Microlax will be performed the day before the operation, then the same day of the operation 2 to 4 hours before.

General anaesthesia

General anesthesia will be performed with propofol , combined with a low dose of sufentanil as an analgesic. Ventilation will be ensured by means of a laryngeal mask and anesthesia maintained with either propofol in TCI (intravenous anesthesia with objective concentration), or by inhalation with sevoflurane .

Supportive treatments

Supportive treatments planned during hospitalization include level 1 or 2 analgesics or antiemetics if needed, intravenously or orally :

- Paracetamol 500 mg orally or Perfalgan 1 g IV
- Nefopam (Acupan) 20 mg / 2 mL
- Ondansetron 8 mg IV

7.3 Description of the traceability elements that accompany the investigational drug (s)

The microwave energy treatment is performed intraoperatively. The monitoring of the cumulative duration of treatment and the cumulative quantity of energy delivered are followed in real time during the intervention thanks to the generator's treatment monitor. These two parameters are noted in the observation book.

The references of the microwave applicator (single use) used for the treatment are noted in the observation book

7.4 Authorized and prohibited treatments (drug, non-drug, surgical), including rescue drugs

No medical, non-medical or surgical treatment is prohibited during the research period.

7.5 Methods of monitoring adherence to treatment

No method of monitoring compliance is necessary within the framework of this research protocol.

8 EFFICIENCY EVALUATION

8.1 Description of evaluation parameters

Primary endpoint

The main endpoint is the degree of necrosis of the index tumor, assessed by multiparametric MRI of the prostate performed on D7 (+2 days if necessary) after treatment, in the Radiology A department of Cochin hospital.

Complete necrosis is defined by the absence of enhancement on dynamic contrast MRI in the target area delimited just before treatment on multiparametric MRI.

Secondary criteria

1 / The minimum and maximum necrosis margins outside the target volume delimited on the preoperative MRI will be measured by comparing the multiparametric MRI of the prostate carried out 7 days after the treatment with the multiparametric MRI of the prostate carried out before the treatment.

2 / Sexual tolerance will be assessed by the IIEF (erectile function) and MSHQ- Ej (ejaculatory function) self-questionnaires (provided in the appendix). These self-questionnaires will be completed by the patient during the follow-up visits on D7, 2 and 6 months after the intervention.

3 / Urinary tolerance will be assessed by the IPSS and IPSS-QDV self-questionnaires (provided in the appendix), the cytobacteriological examination of the urine, the maximum urine output, the total serum Prostate Specific Antigen (PSA), and the introduction of any additional treatment for urinary purposes, on D7, 2 and 6 months after the operation.

4 / The carcinological evolution will be evaluated by multiparametric MRI of the prostate and targeted biopsies at the level of the treated volume, 6 months after the operation.

8.2 Methods and schedule for measuring, collecting and analyzing parameters for evaluating effectiveness

All of the evaluation criteria will be reported in the research observation book.

The evaluation of the primary endpoint will be carried out by Dr Alexandre Schull , radiologist and research investigator, by comparative study of preoperative and postoperative prostate MRIs on D7 of treatment.

The evaluation of the precision of the treatment will be carried out by Dr Alexandre Schull , at the same time as the main endpoint.

The evaluation of sexual and urinary tolerance will be carried out under the direction of Dr Nicolas Barry Delongchamps . The self-questionnaires will be completed directly by the patient. Urinary and plasma samples will be taken in the urology consultation at Cochin hospital, and analyzed at the central laboratory at Cochin hospital (cytobacteriological examination of urine and total serum PSA assay). The measurement of the maximum urinary flow will be carried out within the consultation of the urology department of the Cochin hospital.

The multiparametric MRI performed 6 months after the operation will be scheduled in the same department as that performed at screening and at 2 months, and analyzed by Dr Alexandre Schull .

The control prostate biopsies, carried out 6 months after the operation, will be performed in the radiology department of Cochin hospital, under the direction of Dr Alexandre Schull , and will be analyzed in blind pathology of the MRI result.

All MRI scans of each patient will be anonymized and then analyzed blind by a second radiologist, Dr Jean-Paul Abecassis . For each patient, MRI scans will be analyzed in a random order of the examination chronology. The concordance of the measurement of the evolution of the volume of necrosis by the two radiologists will be studied.

9 COMMITTEES SPECIFIC RESEARCH

9.1 Scientific committee

No scientific committee will be set up under this protocol.

9.2 Steering committee

The members of the steering committee are the following:

- Dr Nicolas BARRY-DELONGCHAMPS, urology department in Cochin
- Dr Alexandre SCHULL, radiology department in Cochin
- Dr Hendy ABDOUL, Clinical research unit in Cochin
- Karine GOUDE-ORY, Delegation for Clinical Research and Innovation (DRCI) Saint-Louis Hospital

Role:

- define the general organization of research, coordinate information, initially determine the methodology and monitor the progress of the research.
- propose procedures to be followed during the research, taking note of the recommendations of the independent monitoring committee. The DRCI promoter remains the decision maker.

It will meet according to the needs of the study.

9.3 Critical Events Validation Committee

As this study does not pose any particular risk to the subjects included, the committee will not be formed.

10 SAFETY ASSESSMENT - RISKS AND CONSTRAINTS ADDED BY RESEARCH

10.1 Description of the safety assessment parameters

Cardio-respiratory constants intraoperatively and immediately postoperatively during the first 2 hours following the patient's awakening.

The anatomical extension of the intra-prostatic necrosis caused by the treatment under study, in particular the radiological appearance of the adjacent anatomical structures.

The sexual tolerance of the treatment, in particular on the erectile and ejaculatory level.

Urinary tolerance of the treatment on the plane of infection and functionally.

10.2 Methods and schedule for measuring, collecting and analyzing safety assessment parameters

Post-intervention surveillance unit :

All treated patients will be monitored intraoperatively according to the best practice recommendations of the French Anesthesia and Resuscitation Society. Immediately postoperatively, patients will be monitored in a post-interventional monitoring unit for two hours, with continuous evaluation of their cardio-respiratory constants.

This type of monitoring is not a constraint added by research.

Multiparametric MRI of the prostate :

MRI will assess the extent of postoperative intra-prostatic necrosis and ensure that there is no involvement of peri-prostatic anatomical structures. It will be performed 7 days after the operation.

Assessment of sexual tolerance :

Sexual tolerance will be assessed by the IIEF (erectile function) and MSHQ- Ej (ejaculatory function) self-questionnaires (provided in the appendix). These self-questionnaires will be completed by the patient during the follow-up visits on D7, 2 and 6 months after the intervention.

Assessment of urinary tolerance :

Urinary tolerance will be assessed before discharge from hospital by monitoring spontaneous postoperative resumption of voiding.

A cytobacteriological examination of the urine will rule out any postoperative bacteriuria. It will be implemented for the visit to the 7 th postoperative day.

The urinary symptoms will then be evaluated by the IPSS and IPSS-QDV self-questionnaires (provided in the appendix) and the measurement of the urine flow during the follow-up visits on D7, 2 and 6 months after the operation.

10.3 Definitions

According to article R1123-46 of the Public Health Code :

- **Adverse event (AE)**

Any harmful manifestation occurring in a person who lends itself to research involving the human person, whether or not this manifestation is linked to the research or to the product on which this research relates.

- **Adverse reaction to a medical device (MD) or an *in vitro* diagnostic medical device (IVDD)**

Any harmful and unwanted reaction to a medical device or any incident which could have caused this reaction if appropriate action had not been taken, in a person who is amenable to investigation or in the user of the medical device or any effect linked to a failure or alteration

of an *in vitro* diagnostic medical device and harmful to the health of a person who is suitable for research.

- **Serious adverse event or effect**

Any event or undesirable effect which leads to death, endangers the life of the person undergoing the research, necessitates hospitalization or prolongation of hospitalization, causes significant or lasting incapacity or handicap, or results in by a congenital anomaly or malformation, and with regard to the drug, regardless of the dose administered.

- **Unexpected side effect**

Any undesirable effect whose nature, severity or evolution does not match the information relating to the products, acts, practices and methods used during the research.

<u>According to article R5212-15 of the Public Health Code</u> :

- **Incident**
  - · Harmful and unintended reaction occurring when using a medical device for its intended purpose;
  - · Harmful and unwanted reaction resulting from use of a medical device that does not comply with the manufacturer's instructions;
  - · Any malfunction or alteration of the characteristics or performance of a medical device;
  - · Any erroneous indication, omission and insufficiency in the instruction manual, user manual or maintenance manual.

<u>According to article R.1123-46 of the Public Health Code and the notice to the promoters of clinical trials on medical devices and *in vitro* diagnostic medical devices (ANSM) :</u>

- **New fact**

Any new data that may lead to a reassessment of the ratio of benefits and risks of the research or of the product being researched, to modifications in the use of this product, in the conduct of research, or in documents relating to the research. research, or to suspend or interrupt or modify the research protocol or similar research. For trials involving the first administration or use of a health product in people without any medical conditions: any serious adverse reaction.

Examples :
a) any clinically significant increase in the frequency of occurrence of an expected serious adverse reaction;
b) suspected unexpected serious adverse reactions occurring in participants who have completed the trial and which are reported by the investigator to the sponsor, as well as any follow-up reports;
c) any new fact concerning the conduct of the clinical trial or the use of the medical device, when this new fact is likely to endanger the safety of the participants.
By way of example:

- a serious adverse event likely to be linked to the investigations and diagnostic procedures of the test and which could modify the course of this test,
- a significant risk for the trial population, for example a lack of efficacy of the medical device used in the treatment of a life-threatening disease,
- significant results from a completed preclinical study that may call into question the risk assessment in relation to the expected benefit (such as a biomechanics study),
- early termination or temporary interruption for safety reasons of a test conducted with the same medical device in another country,

- an unexpected serious adverse event linked to a non-experimental health product necessary for the performance of the trial (eg: "challenge agents", rescue treatment)

d) the recommendations of the Independent Oversight Committee (CSI), if applicable, if they are relevant for the safety of persons,

e) any unexpected serious adverse event transmitted to the sponsor by another sponsor of a clinical trial on the same medical device in a third country.

10.4 Roles of the investigator

The investigator must **assess its seriousness for each adverse event** and record all serious and non-serious adverse events in the observation log (CRF) .

The investigator must **document** the serious adverse events as well as possible and, if possible, provide the definitive medical diagnosis.

The investigator should **assess the intensity** of the adverse events using general terms :

- Light : tolerated by the patient, not interfering with his daily activities
- Moderate : uncomfortable enough to interfere with daily activities
- Severe : which prevents daily activities

Likewise, each adverse event will be classified according to the Clavien Dindo classification , commonly used in the evaluation of surgical complications.

The investigator must **assess the causal link of** serious adverse events with the experimental medical device, and / or its implementation and / or the acts / procedures added by the research and with other possible treatments.

The method used by the investigator, based on the WHO method (WHO Uppsala Monitoring Center), is based on the following 4 causal terms :

- Certain
- Probable / plausible
- Possible
- Unlikely

Their definition is presented in the following table (extract from WHO-UMC causality categories , version of 04/17/2012) .

TABLE: WHO-UMC causality categories (extract)

**Causality term**	Assessment criteria *
**Certain**	· Event or laboratory test abnormality, with plausible time relationship to drug intake **<br>· Can not be Explained by disease or other drugs<br>· Response to withdrawal plausible (pharmacologically, Pathologically)<br>· Event definitive pharmacologically or phenomenologically (ie an objective and specific medical disorder gold reconnu pharmacological phenomenon)<br>· Rechallenge Satisfactory , if Necessary
**Likely / Likely**	· Event or laboratory test abnormality, with reasonable time relationship to drug intake<br>· Unlikely to be Attributed To disease or other drugs<br>· Response to withdrawal Clinically reasonable<br>· Rechallenge not required
**Possible**	· Event or laboratory test abnormality, with reasonable time relationship to drug intake<br>· Could aussi be Explained by disease or other drugs<br>· On Information drug withdrawal May be lacking or unclear
**Unlikely**	· Event or laboratory test abnormality, with a time to drug intake<br>· That Makes a relationship unlikely (but not impossible)<br>· Disease or other drugs Provide plausible Explanations

* All points should be reasonably complied with

** Or study procedures

The investigator will specify whether the serious adverse event follows an incident related to the experimental medical device and / or its implementation .

The investigator having knowledge of an incident must report it to the promoter without delay upon becoming aware of it. He fills out the incident or risk of incident reporting form which can be downloaded from the ANSM website and sends it by fax without delay as soon as he becomes aware of the Vigilance sector of the DRCI (AP-HP) on 01 44 84 17 99.

10.4.1 Serious adverse events requiring immediate notification by the investigator to the sponsor

In accordance with article R.1223-49, the investigator notifies the sponsor without delay from the day on which he becomes aware of all the serious adverse events that have occurred during a research mentioned in 1 ◦of article L .1121-1, with the exception of those listed in the protocol (see section 10.4.2.3) and, where applicable, in the brochure for the investigator as not requiring immediate notification (extract from l article R.1123-49 of the Public Health Code). The investigator notifies these events within an appropriate timeframe, taking into account the specificities of the research and the serious adverse event as well as any indications appearing in the protocol or the brochure for the investigator. This notification is the subject of a written report and is followed by additional detailed written reports.

A serious adverse event has one of the following criteria :

1- event that leads to death,
2- event that endangers the life of the person who lends itself to the research,
3- event that requires hospitalization or prolongation of hospitalization,
4- event which causes a significant or lasting incapacity or handicap,
5- event which results in a congenital anomaly or malformation.

10.4.2 Specific features of the protocol

10.4.2.1 Other events requiring immediate notification by the investigator to the sponsor

- Postoperative adverse events of grade ≥ 3b according to the Clavien-Dindo classification
- Any SAE following an incident in the microwave delivery procedures (eg : energy dose and duration of treatment not in accordance with the chart)
- Secondary cancers

These adverse events must be notified to the sponsor by the investigator without delay from the day on which he becomes aware of them , under the same terms and conditions as the serious adverse events (see section 10.4.4).

10.4.2.2 Serious adverse events not requiring immediate notification by the investigator to the sponsor

These serious adverse events are only collected in the “ adverse event ” part of the observation log.

- Natural and usual course of the pathology :

- hospitalization scheduled for the follow-up of the pathology studied,
- hospitalization for routine treatment or monitoring of the pathology studied not associated with a deterioration of the subject's condition,
- worsening of the pathology studied : carcinological evolution requiring radical treatment, as proposed in the context of current care

- Special circumstances
  - prolongation of hospitalization in a post-intervention surveillance unit beyond the 3-hour period provided for in the protocol
  - postoperative complications of grade <3b according to the Clavien-Dindo classification
  - hospitalization for a pre-existing pathology
  - hospitalization for medical or surgical treatment scheduled before the research
  - admission for social or administrative reasons
  - emergency room visit (<12 hours)

- Adverse events likely to be related to treatments / acts prescribed in the context of care during research monitoring

  The investigator, like any health professional, must notify these events to the applicable health vigilance. Examples : Regional Health Agency, establishment quality department, Regional Pharmacovigilance Center, local medical device vigilance correspondent (ANSM), etc.

- Adverse events occurring at a distance from the act of implementing the experimental medical device. The time limit from which the causal link between the occurrence of the SAE and the act of implementing the experimental medical device can be excluded is 30 days. This delay is commonly accepted after surgery.

10.4.3 Period for prompt notification of SAEs by the investigator to the sponsor

The investigator should promptly notify the sponsor of the serious adverse events as defined in the relevant section:

- to from the date of intervention (date of application of microwave)
- during the entire monitoring period the participant under research,
- without time limit, when the EIG is likely to be due to the medical device and / or to acts / procedures / examinations specific to research.

10.4.4 Procedures and deadlines for notification to the promoter

The initial SAE notification is the subject of a written report signed by the investigator using a specific SAE notification form provided for this purpose in the observation book.

Each item of this document must be completed by the investigator to allow the sponsor to perform a relevant analysis.

The initial notification of a serious adverse event to the sponsor must be followed quickly by a (or more) detailed additional report (s), written (s) making it possible to follow the evolution of the case in vigilance or complete the information.

As far as possible, the investigator will transmit any document that may be useful to the sponsor (medical reports, laboratory results, results of additional examinations, etc.). These documents must be made anonymous. In addition, they must be completed with the following information : acronym of the research, number and initials of the participant.

Any adverse event will be monitored until it is completely resolved (stabilization at a level deemed acceptable by the investigator or return to the previous state) even if the participant has left the research.

The initial notification, EIG monitoring reports and any other document will be sent to the promoter represented by its Vigilance sector exclusively by fax to **01 44 84 17 99** .

In the case of a search with e-CRF:
- the investigator completes the EIG notification form in the e-CRF, validates it, prints it, signs it and then sends it by fax.
- in case of impossibility of connection to the e-CRF, the investigator will complete, sign and send the EIG notification form inserted in appendix 2. As soon as the connection is re-established, he will regularize by completing the notification form EIG of the e-CRF.

The investigator must respond to any request for additional information from the sponsor.
For any questions relating to the notification of an adverse event, you can contact the Vigilance sector by email : vigilance.drc@aphp.fr

10.5 Roles of the promoter

The promoter represented by its Vigilance sector continuously assesses the safety of each medical device, throughout the research.

10.5.1 Analysis and reporting of serious adverse events

The promoter assesses:
- the **seriousness** of all adverse events reported to it,
- their **causal link** with each experimental medical device and / or its implementation action and / or specific acts / procedures / examinations added by research and with other possible treatments. All serious adverse events for which the investigator and / or the sponsor considers that a causal relationship with the investigational medical device can be reasonably expected are considered to be suspected serious adverse reactions.
- the **expected or unexpected nature** of the side effects.
  Any serious adverse reaction the nature, severity or course of which does not match the information given in the instructions for use when the medical device is the subject of a CE marking or in the brochure for the investigator when the product is not authorized, is considered unexpected.
  The assessment of the expected / unexpected nature of a serious adverse reaction is carried out by the sponsor represented by its Vigilance sector on the basis of the information described below (see section 10.5.1.1) **.**

Reference safety information

❖ For serious adverse events likely to be related to the experimental medical device and / or its implementation and expected , please refer to the " TATO ® from Biomedical " instructions for use .

The expected serious adverse events are related to the thermal effect of microwaves, leading to circumscribed necrosis around the applicator. The extension of the necrosis zone can be linked either to an imprecision of the guidance, or to too wide diffusion of the thermal effect of the microwaves.

❖ An extension of the area of necrosis beyond the target area can lead, within a period of a few hours to around 30 days :

- a necrosis of the rectal wall can cause :
  - a recto-prostatic or recto-urethral fistula,
  - acute prostatitis,
  - mild, moderate or massive rectal bleeding with acute hemorrhagic syndrome,
- an erectile dysfunction linked to the achievement of neurovascular strips
- the problems related to voiding urethral damage, and characterized by urinary urgency, urinary frequency, and / or voiding burns,
- a mild to severe hematuria, with risk of acute haemorrhagic syndrome.

The risk of extension of the necrosis zone will nevertheless be precisely evaluated on the postoperative MRI performed on D7.

❖ Adverse events linked to acts / procedures / examinations specific to research :

- Preparation for the operation : refer to the SPCs Oflocet ® , Bactrim ® and Microlax ® / Normacol ®

- Immobilization: postoperative thromboembolic complications

- General anaesthesia :
  - postoperative pneumonia linked to tracheal intubation,
  - anaphylactic shock secondary to anesthetic drugs : refer to the SPCs for Propofol ® or Sevorane ®

- Supportive treatment : refer to the SPCs for Acupan ®, paracetamol and ondansetron administered.

- MRI : allergy or intolerance to Gadolinium injected intravenously (refer to SPC Gadolinium), claustrophobia

The promoter declares:

- any suspicion of an unexpected serious adverse reaction due to an investigational medical device under investigation (SUSAR)
- all serious undesirable events likely to be linked to the act of implementing the experimental medical device being the subject of the research.
- any incident leading to the occurrence of a serious adverse event.

NB : the sponsor declares any incident that led to the occurrence of a serious adverse event to the ANSM (mailbox dedicated to the transmission of data relating to clinical trials of MDs, MDs DIV and ANSM material vigilance unit) .

The sponsor declares any suspicion of an unexpected serious adverse reaction (SUSAR) and any serious adverse event that may be linked to the act of implementing the medical device that has occurred in France and outside the national territory concerned, within the regulatory deadlines, to the " National Agency for the Safety of Medicines and Health Products (ANSM):

- The initial declaration must be made without delay from the day on which the sponsor became aware of the case of an unexpected serious adverse effect and of a serious adverse event that may be linked to the act of implementing the medical device that led to the death or life-threatening and within 15 days from the day on which the sponsor became aware of it for the case of other unexpected serious adverse effects and serious adverse event that may be linked to the act of implementing the medical device ;

- In the event of an unexpected serious adverse reaction or serious adverse event that may be related to the act of implementing the medical device resulting in death or endangering life, the relevant additional information is notified to the National Agency for drug and health product safety within eight days.

- In other cases of unexpected serious adverse reactions and serious adverse events that may be related to the act of implementing the medical device, the relevant additional information is sent to the National Agency for the Safety of Medicines and Health Products in a period of fifteen days.

The sponsor informs all the investigators concerned of any data that could have an unfavorable impact on the safety of the people who are involved in the research.

10.5.2 Analysis and declaration of other safety data

According to article R.1123-46 of the Public Health Code and the notice to the promoters of clinical trials on medical devices and *in vitro* diagnostic medical devices (ANSM), a new fact is defined by any new data that may lead to a re-evaluation of the ratio of the benefits and risks of the research or of the product being researched, to modifications in the use of this product, in the conduct of research, or in documents relating to research , or to suspend or interrupt or modify the research protocol or similar research. For trials involving the first administration or use of a health product in people without any medical conditions: any serious adverse reaction.

The promoter informs without delay from the day on which he becomes aware of it the competent authority and the committee for the protection of persons of the new facts and, if necessary, of the urgent safety measures taken .

Following the initial declaration relating to a new fact, the promoter sends the competent authorities in the form of a follow-up report on the new fact, any relevant additional information relating to this new fact within a maximum period of 15 days from the when he has this information.

If a suspicion of an unexpected serious adverse reaction (SUSAR) or a serious adverse event that may be linked to the procedure for implementing the MD meets the definition of a new fact , the corresponding event must be the subject of a double declaration by the promoter, according to the terms and deadlines previously mentioned.

10.5.3 Annual safety report

The sponsor must establish once a year throughout the duration of the clinical trial an annual safety report (RAS or annual safety report - [ASR]) including in particular:
- an analysis of the safety of people who lend themselves to research,
- a list of all suspected serious adverse reactions and a list of serious adverse events that may be related to the act of implementing the medical device, which occurred during the period covered by the report,
- summary tables of all the serious undesirable effects that have occurred since the start of the research.

The report is sent to the ANSM and the CPP within 60 days after the anniversary date corresponding to the date of the first patient included in the research.

10.6 Independent Supervisory Committee

An Independent Supervisory Committee (CSI) is planned as part of this research. Its main mission is to be a safety data monitoring committee. A preliminary meeting of the CSI is planned before the first inclusion of the first topic.
The members of the CSI are :
- Urologist (to be expected)
- Dr. Cédric LAOUENAN, Department of Biostatistical Epidemiology and Clinical Research, Bichat Hospital, Paris
- Dr. Caroline ESCOURROU, radiology, liberal doctor, Paris

All of the missions as well as the precise operating methods of the CSI are described in the CSI charter of the study.

The CSI has an advisory function, the promoter remains the decision maker.

11 DATA MANAGEMENT

11.1 Data collection methods

All the information required by the protocol must be provided in the observation notebook and an explanation given by the investigator for each missing data.
The data will have to be reported in the electronic observation notebooks as they are obtained, whether they are clinical or para-clinical data .
Erroneous data detected in the observation notebooks will be the subject of a request for correction. With the electronic observation book, the modifications will be traced by the computer tool.

11.2 Identification of the data collected directly in the FIUs which will be considered as source data

This will mainly be the following data:
- data relating to the patient: date of birth (month / year), age and initials, surname and first name.

- data concerning the various examinations carried out in the context of research (multiparametric MRI results, digital rectal examination, biological assessment)
- data collected from patient questionnaires (IIEF, MSHQ- Ej , IPSS and IPSS-QDV)
- adverse events collected

11.3 Right of access to source data and documents

Data access

In accordance with GCP :
- the promoter is responsible for obtaining the agreement of all the parties involved in the research in order to guarantee direct access to all the places where the research is carried out, to the source data, to the source documents and to the reports in a purpose of quality control and audit by the promoter or inspection by the competent authority,
- the investigators will make available to the people responsible for monitoring, quality control, audit or inspection of research involving the human person, the documents and individual data strictly necessary for this control, in accordance with the legislative provisions and regulations in force (Articles L.1121-3 and R.5121-13 of the Public Health Code).

Source documents

The source documents being defined as any document or original object allowing to prove the existence or the accuracy of a data or a fact recorded during the research will be kept according to the regulations in force by the investigator or by the hospital if it is a hospital medical record.

The data considered as source data will be the medical file, consultation reports, biological results, radiological images and their report.

Confidentiality of data

The persons in charge of the quality control of research involving the human person (article L.1121-3 of the public health code), will take all the necessary precautions to ensure the confidentiality of information relating to research, people who lend themselves to it and in particular as regards their identity and the results obtained.
These people, like the investigators themselves, are subject to professional secrecy (under the conditions defined by articles 226-13 and 226-14 of the penal code).
During the research involving the human person or at its end, the data collected on the people who lend themselves to it and transmitted to the promoter by the investigators (or any other specialized interveners) will be made non- identifying .
They must not in any case show the names of the persons concerned or their address in clear. Only the initials of the first and last name will be recorded, accompanied by a research-specific coded number indicating the order of inclusion of the subjects.
The promoter will ensure that each person who takes part in the research has given his written consent for access to the individual data concerning him and strictly necessary for the quality control of the research.

11.4 Data processing and retention of documents and data

Identification of the person responsible and the place (s) of the management of the data processing (s)

The data entry will be carried out by the investigators on an electronic medium via an internet browser.
Data analysis will be performed by a biostatistician from URC Paris Descartes Cochin Necker.

Data entry

Data entry will be carried out electronically via an internet browser.

Data processing (CNIL) in France

This research falls within the framework of the “ Reference Methodology for the processing of personal data carried out within the framework of intervention research involving the human person ” (modified MR-001). The AP-HP, research promoter, has signed a commitment to comply with this “ Reference Methodology ”.

Archiving

The documents specific to research involving the human person relating to a medical device or an in vitro diagnostic medical device will be archived by the investigator and the sponsor for a period of 15 years after the end of the research.

This indexed archiving includes in particular :

- A sealed envelope for the investigator containing a copy of all information notes and consent forms signed by all persons at the center who participated in the research ;
- A sealed envelope for the sponsor containing a copy of all information notes and consent forms signed by all the people at the center who participated in the research;
- The " research " workbooks for the Investigator and the promoter comprising :
    - the successive versions of the protocol (identified by the version number and date), its annexes
    - the authorizations ANSM and opinions of the PPC
    - the correspondence of letters,
    - the inclusion list or register,
    - the specific annexes looking
    - the final research report.
- The data collection documents

11.5 Ownership data

The AP-HP is the owner of the data and no use or transmission to a third party may be made without its prior consent.

12 STATISTICAL ASPECTS

12.1 Assumptions for calculating the number of subjects required and result

A phase II study design was used to calculate the number of subjects considering that a complete necrosis rate of 99% would be desirable. If this rate were 60% or less the intervention would be considered ineffective.

Consistent with these assumptions and with an alpha risk of 0.1 and a beta risk of 0.1, a total of 5 patients should be required. In order to be able to study the tolerance of the intervention, an additional number of 5 patients will be provided, ie a total of 10 patients to be included.

12.2 Description of the planned statistical methods including the schedule for the planned interim analyzes

The analysis will be carried out using R software (http://cran.r-project.org). Statistical analysis will be carried out at the end of the collection, entry and consistency check of the data. No interim analysis is foreseen in this protocol.

For the analysis of the primary endpoint, the number and percentage of patients with complete necrosis of the target volume 7 days after treatment will be given. The 95% confidence interval (one-sided) will be calculated using the exact Clopper -Pearson method.

For secondary analyzes :
The minimum and maximum necrosis margins will be given for each of the 10 patients (a median and an interquartile range will possibly be calculated). The scores obtained on the IIEF (erectile function), MSHQ- Ej (ejaculatory function) IPSS and IPSS- QDV self-questionnaires at D7 2 and 6 months will be given for each of the 10 patients (a median and an interquartile range will possibly be calculated). Maximum urine output and total serum Prostate Specific Antigen (PSA) will be reported for each patient (a median and interquartile range will optionally be calculated).
The result of the cytobacteriological examination of the urine and the introduction of any additional urinary treatment on D7, 2 and 6 months after the operation will be specified for each patient. A percentage accompanied by its 95% confidence interval will be given for each of the two variables.

13 QUALITY CONTROL AND ASSURANCE

Each intervention research project involving the human person supported by AP-HP is classified according to the predicted risk incurred by the people involved in the research thanks to the classification of intervention research involving the human person with AP-HP promotion .

13.1 General organization

The promoter must ensure the safety and respect of the people who have agreed to participate in the research. It must put in place a quality assurance system allowing the best possible monitoring of the progress of research in the investigating centers.

To this end, the promoter mandates Clinical Research Associates (ARC) whose main mission is to carry out regular follow-up visits to the research sites after having carried out the opening visits.
The objectives of research monitoring, as defined in Good Clinical Practices, (GCP § 5.18.1) are to verify that:

- the rights, safety and protection of people who lend themselves to research are satisfied,
- the data reported is accurate, complete and consistent with the source documents,
- the research is carried out in accordance with the protocol in force, the GCPs and the legislative and regulatory provisions in force.

Center opening strategy

The opening strategy of the centers set up for this research is determined thanks to the adapted monitoring plan. Before any inclusion, the recruiting center will be opened by an on-site installation visit, for the implementation of the protocol and acquaintance with the various stakeholders in research involving the human person .

Extent of center monitoring

In the case of this **D-** risk research, the choice of an appropriate monitoring level was weighted according to the complexity, impact and budget of the research. To this end, the promoter, in agreement with the coordinating investigator, determined the logistical and impact score which made it possible to obtain the level of monitoring to be implemented on the research : **high** level **.**

These different levels are defined in the monitoring charter for Research Involving the Human Person.

13.2 Quality control

A Clinical Research Associate (ARC) appointed by the sponsor will ensure the proper performance of the research, the collection of the data generated in writing, their documentation, recording and report, in accordance with the Standard Operating Procedures applied in within the DRCI and in accordance with Good Clinical Practices as well as the legislative and regulatory provisions in force.

The investigator and the members of his team agree to make themselves available during Quality Control visits carried out at regular intervals by the Clinical Research Associate. During these visits, the following will be reviewed:

- written consent ;
- compliance with the research protocol and the procedures defined therein ;
- quality of the data collected in the observation notebook: accuracy, missing data, consistency of the data with the "source" documents (medical files, appointment books, originals of laboratory results, etc.) ;
- management of the treatments used.

13.3 Observation book

All the information required by the protocol must be recorded in the observation notebooks. Data should be collected as and when it is obtained, and recorded in these notebooks explicitly. Each missing item must be coded.

This electronic observation book will be set up in the center using an Internet data collection medium. A help document for the use of this tool will be provided to investigators.

The investigator's filling in the observation book via the Internet thus enables the ARC to quickly and remotely visualize the data. The investigator is responsible for the accuracy, quality and relevance of all data entered. In addition, when entered, this data is immediately verified through consistency checks. As such, it must validate any change in value in the CRF. These modifications are subject to an audit trail . A justification can optionally be included as a comment. A paper printout will be requested at the end of the study, authenticated (dated and signed) by the investigator. A copy of the authenticated document intended for the sponsor must be archived by the investigator.

13.4 Management of non-conformities

Any event occurring following non-compliance with the protocol, standard operating procedures, good clinical practices or legislative and regulatory provisions in force by an investigator or any other person involved in the conduct of the research must be the subject of a declaration of non-compliance to the promoter.

These non-conformities will be managed in accordance with the promoter's procedures.

13.5 Audit / inspections

The investigators undertake to accept the quality assurance audits carried out by the sponsor as well as the inspections carried out by the competent authorities. All data, documents and reports may be subject to audits and regulatory inspections without the possibility of medical confidentiality .

An audit can be carried out at any time by people appointed by the promoter and independent of the research managers. Its objective is to ensure the quality of research, the validity of its results and compliance with the law and regulations in force.

Those who direct and supervise research agree to abide by the requirements of the sponsor and the competent authority with respect to an audit or inspection of research.

The audit may apply to all stages of research, from the development of the protocol to the publication of results and the classification of data used or produced in the context of the research.

13.6 Responsibilities of the Principal Investigator

Before starting the research, each investigator will provide the representative of the research sponsor with his personal, dated and signed curriculum vitae, including his RPPS number. The CV must include previous participations in research and training related to clinical research.

Each investigator will undertake to respect the obligations of the law and to carry out the research according to the GCP, respecting the terms of the declaration of Helsinki in force.

The principal investigator of each participating center will sign a responsibility agreement (DRCI type document) which will be given to the promoter's representative.
The investigators and their collaborators will sign a delegation of functions form specifying the role of each.

14 ETHICAL AND LEGAL ASPECTS

14.1 Procedures for informing and obtaining the consent of persons participating in the research

In accordance with article L1122-1-1 of the Public Health Code, no intervention research involving the human person may be carried out on a person without their free and informed consent, collected in writing after the information has been delivered to them. provided for in article L. 1122-1 of the same code.

A **3-day** cooling-off period is left to the person between the time they are informed and the time they sign the consent form.

The free, informed and written consent of the person is obtained by the investigator, or by a doctor who represents him before the inclusion of the person in the research and before the performance of any act required by intervention research involving the human person. .

The information note and a copy of the consent form dated and signed by the person involved in the research as well as by the investigator or the doctor representing him are given to the person prior to his participation in the research.

In addition, the investigator will specify in the person's medical file his participation in the research, the procedures for obtaining his consent as well as the procedures for issuing the information with a view to collecting it. He keeps the original copy of the personal consent form, dated and signed.

14.2 Prohibition for the person to participate in another research or exclusion period provided for at the end of the research, if applicable

The exclusion period defined in the context of this research is 12 months, a period necessary for complete healing of prostate tissue.
During this period, the subject cannot participate in another intervention research protocol involving the human person. However, subjects can participate in other non-interventional research.

14.3 Compensation of subjects

No compensation is provided for patients to compensate for constraints related to research.

14.4 Authorization of the premises

The research is carried out in care services on people presenting a clinical condition for which the services are competent and require acts usually performed in the context of their activities. Therefore, it is not necessary to have a specific location authorization for the search.

15 LEGAL OBLIGATIONS

15.1 Role of the promoter

The Public Assistance Hospitals of Paris (AP-HP) is the promoter of this research and by delegation the Delegation for Clinical Research and Innovation (DRCI) carries out its missions, in accordance with article L.1121-1. of the public health code. THE Assistance Publique - Hôpitaux de Paris reserves the right to interrupt the search at any time for medical or administrative reasons; in this event, a notification will be provided to the investigator

15.2 Request for an opinion from the CPP personal protection committee

The AP-HP as promoter obtains for intervention research involving the human person relating to a medical device or an in vitro diagnostic medical device, prior to its implementation, the favorable opinion of the concerned CPP, within the framework of of its powers and in accordance with the laws and regulations in force.

15.3 Authorization request from ANSM

The AP-HP as promoter obtains for intervention research involving the human person relating to a medical device or an in vitro diagnostic medical device, prior to its implementation, the authorization of the ANSM, within the framework of of its powers and in accordance with the laws and regulations in force.

15.4 Commitment to comply with the “ Reference Methodology ” MR 001

The AP-HP, research promoter, has signed a commitment to comply with this “ Reference Methodology”.

15.5 Research modifications

Any substantial modification made to the protocol by the coordinating investigator must be sent to the sponsor for approval. After this agreement, the promoter must obtain prior to its implementation a favorable opinion from the CPP and an authorization from the ANSM within the framework of their respective competences.
The information notice and the consent form may be revised if necessary, in particular in the event of a substantial modification of the research or the occurrence of adverse effects.

15.6 Final research report

The final report of the intervention research involving the human person mentioned in article R1123-60 of the CSP is drawn up and signed by the promoter and the investigator. A summary of the report drawn up according to the reference plan of the competent authority must be sent to the competent authority within one year, after the end of the research, corresponding to the end of the participation of the last person who is willing.

16 FINANCING AND INSURANCE

16.1 Source of funding

This research is funded by the company KOELIS and is promoted by the APHP (DRCI).

16.2 Insurance

The Promoter takes out insurance for the entire duration of the research guaranteeing its own civil liability as well as that of any doctor involved in carrying out the research. It also provides full compensation for the harmful consequences of research for the person who lends itself to it and their beneficiaries, unless it is proven that the damage is not attributable to its fault or that of any party involved, without that may be opposed the act of a third party or the voluntary withdrawal of the person who had initially consented to participate in the research.

Assistance Publique-Hôpitaux de Paris (AP-HP) has taken out insurance from the company HDI-GERLING through BIOMEDIC-INSURE for the duration of the research, guaranteeing its civil liability as well as that of any intervening party. (doctor or staff involved in carrying out the research), in accordance with article L.1121-10 of the CSP.

17 PUBLICATION RULES

The APHP will be obligatorily mentioned in the affiliations of the author (s) of the publications which will result from this research and the AP-HP (DRCI) will be mentioned as promoter.

17.1 Mention of the AP-HP affiliation for projects promoted by the AP-HP

The AP-HP institution will appear under the acronym “AP-HP” first in the address followed precisely by: AP-HP, hospital, department, town, postal code, France.
- If an author has several affiliations, the order in which the institutions are cited (AP-HP, University, INSERM, etc.) does not matter
- However, if the research is funded through an internal call for tenders from AP-HP, the first affiliation should be “ AP-HP ”
- Each of these affiliations must be identified by an address separated by a semicolon
- The AP-HP institution must appear under the acronym " **AP-HP** " first in the address followed precisely by: **AP-HP** , hospital, department, town, postal code, France

17.2 Mention of the AP-HP promoter (DRCI) in the acknowledgments of the manuscript

The research promoter (Assistance Publique - Hôpitaux de Paris) will be mentioned in the " acknowledgments " of the manuscripts (" The sponsor was Assistance Publique - Hôpitaux de Paris (Délégation à la Recherche Clinique et à l'Innovation (DRCI)) ")

17.3 Mention of the funder in the acknowledgments of the manuscript

The research funder (KOELIS) will be mentioned in the “ acknowledgments ” of the manuscripts (“The study was funded by a grant from KOELIS, La Tronche , France”).

This research is registered on the site http://clinicaltrials.gov/ under number *NCT03023345* .

18 BIBLIOGRAPHY

1) Noldus J, Graefen M, Haese A, Henke RP, Hammerer P, HulandH . Stage migration in clinically localized prostate cancer. EurUrol 2000 ; 38 : 74-8.

2) Pardo Y, Guedea F, Aguilo F, et al. Quality-of-life impact of primary treatments for localized prostate cancer in patients without hormonal treatment. Journal of clinical oncology: official journal of the American Society of Clinical Oncology 2010; 28: 4687-96.

3) Bottomley D, Ash D, Al- Qaisieh B *, et al.* Side effects of permanent I125 prostate seed implants in 667 patients treated in Leeds. Radiotherapy and oncology: journal of the European Society for Therapeutic Radiology and Oncology 2007; 82: 46-9.

4) Cornud F, Khoury G, Bouazza N, Beuvon F, Peyromaure M, Flam T, Zerbib M, Legmann P, Delongchamps NB. Tumor target volume for focal therapy of prostate cancer-does multiparametric magnetic resonance imaging allow for a reliable estimation? J Urol. 2014 May ; 191 (5): 1272-9.

5) Delongchamps NB, Rouanne M, Flam T, Beuvon F, Liberatore M, Zerbib M, et al. Multiparametric magnetic resonance imaging for the detection and localization of prostate cancer: combination of T2-weighted, dynamic contrast enhanced and diffusion-weighted imaging. BJU Int 2011; 107: 1411- 8 .

6) Delongchamps NB, Beuvon F, Eiss D, Flam T, Muradyan N, Zerbib M, et al. Multiparametric MRI is helpful to predict tumor focality , stage, and size in patients diagnosed with unilateral low-risk prostate cancer. Prostate Cancer Prostatic Dis 2011; 14: 232- 7 .

7) Lanz C, Cornud F, Beuvon F, Lefèvre A, Legmann P, Zerbib M, Delongchamps NB. Gleason score determination with TRUS-MRI fusion guided prostate biopsies: are we gaining in accuracy? J Urol. 2015 Jul 9. pii : S0022-5347 (15) 04338-4. doi : 10.1016 / j.juro.2015.07.021.

8) Delongchamps NB, Lefèvre A, Bouazza N, Beuvon F, Legman P, Cornud F. Detection of significant prostate cancer with magnetic resonance targeted biopsies - should transrectal ultrasound-magnetic resonance imaging fusion guided biopsies alone be a standard of care? J Urol. 2015 Apr ; 193 (4): 1198-204.

9) Delongchamps NB, Peyromaure M, Schull A, Beuvon F, Bouazza N, Flam T, Zerbib M, Muradyan N, Legman P, Cornud F. Prebiopsy magnetic resonance imaging and prostate cancer detection: comparison of random and targeted biopsies. J Urol. 2013; 189: 493-9.

10) National Cancer Institute. Dynamics of the evolution of mortality rates for the main cancers in France. http://www.e-cancer.fr/toutes-les-actualites/81/4703-la-baisse-de-la-mortalite-par-cancers-saccelere-en-france .

11) Jemal A, Siegel R, Xu J, Ward E. Cancer statistics, 2010. CA Cancer J Clin 2010; 60: 277-300.

12) Delongchamps NB, Singh A, Haas GP. The role of prevalence in the diagnosis of prostate cancer. Cancer Control 2006; 13: 158-68.

13) Salomon L, Bastide C, Beuzeboc P, Cormier L, Fromont G, Hennequin C, Mongiat -Artus P, Peyromaure M, Ploussard G, Renard- Penna R, Rozet F, Azria D, Coloby P, Molinié V, Ravery V , Rebillard X, Richaud P, Villers A, Soulié M; CCAFU members. [CCAFU Recommendations 2013: Prostate cancer]. Prog Urol . 2013 Nov; 23 Suppl 2: S 69-101.

14) D'Amico AV, Whittington R, Malkowicz SB, et al. Biochemical outcome after radical prostatectomy, external beam radiation therapy, or interstitial radiation therapy for clinically localized prostate cancer. JAMA: the journal of the American Medical Association 1998; 280: 969-74.

15) Heidenreich A, Bellmunt J, Bolla M, et al. WATER guidelines on prostate cancer. Part 1: screening, diagnosis, and treatment of clinically localized disease. European urology 2011 ; 59: 61-71.

16) Parker C, Muston D, Melia J, Moss S, Dearnaley D. A model of the natural history of screen-detected prostate cancer, and the effect of radical treatment on overall survival. British journal of cancer 2006 ; 94: 1361-8.

17) Pickles T, Ruether JD, Weir L, Carlson L, Jakulj F. Psychosocial barriers to active surveillance for the management of early prostate cancer and a strategy for increased acceptance. BJU International 2007 ; 100: 544-51.

18) Latini DM, Hart SL, Knight SJ *, et al.* The relationship between anxiety and time to treatment for patients with prostate cancer on surveillance . The Journal of urology 2007 ; 178: 826-31; discussion 831-2.

19) Turkbey B, Mani H, Shah V, RastinehadAR , Bernardo M, Pohida T, et al. Multiparametric 3T prostate magnetic resonance imaging to detect cancer: histopathological correlation using prostatectomy specimens processed in customized magnetic resonance imaging based molds. J Urol 2011 ; 186: 1818-24.

20) Park BK, Park JW, Park SY, Kim CK, Lee HM, Jeon SS, et al. Prospective evaluation of 3-T MRI performed before initial transrectal ultrasound-guided prostate biopsy in patients with high prostate-specific antigen and no previous biopsy. AJR Am J Roentgenol 2011 ; 197: W876-81.

21) Haffner J, Lemaitre L, Puech P, Haber GP, Leroy X, Jones JS, et al. Role of magnetic resonance imaging before initial biopsy: comparison of magnetic resonance imaging-targeted and systematic biopsy for significant prostate cancer detection. BJU Int 2011; 108: 171- 8 .

22) Villers A, Puech P, Mouton D, Leroy X, Ballereau C, Lemaitre L. Dynamic contrast enhanced, pelvic phased array magnetic resonance imaging of localized prostate cancer for predicting tumor volume: correlation with radical prostatectomy findings. J Urol 2006 ; 176: 2432-7 .

23) Dickinson L, Ahmed HU, Allen C, Barentsz JO, Carey B, Futterer JJ, Heijmink SW, Hoskin PJ, Kirkham A, Padhani AR, Persad R, Puech P, Punwani S, Sohaib AS, Tombal

B, Villers A, van der Meulen J, Emberton M. Magnetic resonance imaging for the detection, location , and characterization of prostate cancer: recommendations from a European consensus meeting. Eur Urol. 2011; 59: 477-94.

24) Portalez D, Mozer P, Cornud F, Renard-Penna R, Misrai V, ThoulouzanM, Malavaud B. Validation of the European society of urogenital radiology scoringsystem for prostate cancer diagnosis on multiparametric magnetic resonanceimaging in a cohort of repeat biopsy patients. Eur Urol. 2012; 62: 986-96.

25) Natarajan S, Marks LS, Margolis DJA, Huang J, Macairan ML, Lieu P, et al. Clinical application of a 3D ultrasound-guided prostate biopsy system. UrolOncol 2011 ; 29: 334-42.

26) Baumann M, Mozer P, Daanen V, Troccaz J. Prostate biopsy tracking with deformation estimation. Med Image Anal. 2012 Apr ; 16 (3): 562-76. Epub 2011 May 17.

27) Audenet F, Rouprêt M, Mozer P. [Evolution of modern imaging and development of targeted prostatic biopsies for the diagnosis of prostate cancer]. Prog Urol. 2011 May ; 21Suppl 3: S93-5.

28) Mozer P, Baumann M, Chevreau G, Moreau-Gaudry A, Bart S, Renard- Penna R, et al. Mapping of transrectalultrasonographicprostate biopsies: quality control and learning curve assessmentby image processing. J Ultrasound Med 2009; 28: 455-60.

29) Ukimura O, Desai OM, Palmer S, Valencerina S , Gross M, Abreu AL, AronM , Gill IS. 3-Dimensional Elastic Registration System of Prostate BiopsyLocation by Real-Time 3-Dimensional TransrectalUltrasoundGuidanceWith Magnetic Resonance / Transrectal UltrasoundImage Fusion. J Urol 2012; 187: 1088-1086.

30) Lecornet E, Moore C, Ahmed HU, Emberton M. Focal therapy for prostate cancer: fact or fiction? Urologic oncology 2010; 28: 550-6.

31) Onik G. The male lumpectomy: rationale for a cancer targeted approach for prostate cryoablation . A review. Technology in cancer research & treatment 2004; 3: 365-70.

32) Bahn D, de Castro Abreu AL, Gill IS *, et al.* Focal Cryotherapy for Clinically Unilateral, Low-Intermediate Risk Prostate Cancer in 73 Men with a Median Follow-Up of 3.7 Years. European urology 2012.

33) Ahmed HU, Hindley RG, Dickinson L *, et al.* Focal therapy for localized unifocal and multifocal prostate cancer: a prospective development study. The lancet oncology 2012.

34) Feijoo ER, Sivaraman A, Barret E, Sanchez-Salas R, Galiano M, Rozet F, Prapotnich D, Cathala N, Mombet A, Cathelineau X. Focal High-intensity Focused Ultrasound Targeted Hemiablation for Unilateral Prostate Cancer: A Prospective Evaluation of Oncologic and Functional Outcomes. Eur Urol. 2015 Jul 8. pee : S0302-2838 (15) 00521-7. doi : 10.1016 / j.eururo.2015.06.018.

35) Ghai S, Louis AS, Van Vliet M, Lindner U, Haider MA, Hlasny E, Spensieri P, Van Der Kwast TH, McCluskey SA, Kucharczyk W, Trachtenberg J. Real-Time MRI-Guided Focused Ultrasound for Focal Therapy of Locally Confined Low-Risk Prostate Cancer:

Feasibility and Preliminary Outcomes. AJR Am J Roentgenol . 2015 ; 205 (2): W177-84. doi : 10.2214 / AJR.14.13098.

36) Azzouzi AR, Barret E, Bennet J, Moore C, Taneja S, Muir G, Villers A, Coleman J, Allen C, Scherz A, Emberton M. TOOKAD® Soluble focal therapy: pooled analysis of three phase II studies assessing the minimally invasive ablation of localized prostate cancer. World J Urol. 2015; 33: 945-53.

37) Azzouzi AR, Barret E, Moore CM, Villers A, Allen C, Scherz A, Muir G, de Wildt M, Barber NJ, Lebdai S, Emberton M. TOOKAD (®) Soluble vascular-targeted photodynamic (VTP) therapy: determination of optimal treatment conditions and assessment of effects in patients with localized prostate cancer. BJU Int. 2013; 112: 766-74.

38) Lindner U, Lawrentschuk N, Weersink RA, Davidson SR, Raz O, Hlasny E, Langer DL, Gertner MR, Van der Kwast T, Haider MA, Trachtenberg J. Focal laser ablation for prostate cancer followed by radical prostatectomy: validation of focal therapy and imaging accuracy. Eur Urol. 2010 Jun ; 57 (6): 1111-4.

39) Lepor H, Llukani E, Sperling D, Fütterer JJ. Complications, Recovery, and Early Functional Outcomes and Oncologic Control Following In-bore Focal Laser Ablation of Prostate Cancer. Eur Urol. 2015 May 12. pii : S0302-2838 (15) 00331-0. doi : 10.1016 / j.eururo.2015.04.029.

40) van den Bos W, Muller BG, Ahmed H, Bangma CH, Barret E, Crouzet S, Eggener SE, Gill IS, Joniau S, Kovacs G, Pahernik S, de la Rosette JJ, Rouvière O, Salomon G, Ward JF , Scardino PT. Focal therapy in prostate cancer: international multidisciplinary consensus on trial design. Eur Urol. 2014; 65: 1078-83.

41) Cary KC, Cowan JE, Sanford M, Shinohara K, Perez N, *et al.* Predictors of pathologic progression on biopsy among men on active surveillance for localized prostate cancer : the value of the pattern of surveillance biopsies. *Eur Urol* 2014; 66: 337-42.

42) Nam RK, Saskin R, Lee Y, Liu Y, Law C, Klotz LH, et al. Increasing hospital admission rates for urological complications after transrectal ultrasound guided prostate biopsy. J Urol 2010; 183: 963- 8 .

43) Naber KG, Bishop MC, Bjerklund -Johansen TE, Botto H, Cek M, Grabe M, Lobel B, Palou J, Tenke P. Guidelines on the management of urinary and male genital tract infections. European association of urology 2006
