# Supplementary material for: Feasibility and safety of targeted focal microwave ablation of the index tumor in patients with low to intermediate risk prostate cancer: Results of the FOSTINE trial
Source: PLoS One. 2021 Jul 14;16(7):e0252040. doi: 10.1371/journal.pone.0252040 (PMC8279354; doi:10.1371/journal.pone.0252040)
Supplement: Attachment — Submitted filename: Answer to reviewers_FINAL.docx [file pone.0252040.s006.docx]

Rebbutal letter

The authors would like to thank the reviewers for their comments and suggestions. All comments have been addressed, and corrections were made accordingly.

**Reviewer #1:**

The manuscript entitled ‘Feasibility and safety of targeted focal microwave ablation of the index tumor in patients with low to intermediate risk prostate cancer: results of the FOSTINE trial’ with the aim to assess the feasibility, safety and precision of organ-based tracking (OBT)-fusion targeted focal microwave ablation (FMA), in patients with low to intermediate risk prostate cancer.

Comments

Abstract & Results
Line 36, Line 127, Line 132, Line 177, word IQR to be stated in the text.

This has been corrected accordingly.

Patients and Methods
Sample size calculation
Line 113-117, 1 or 2 tailed test to be stated. Any reason why alpha 0.10 was chosen?

With a risk alpha for two-tailed tests of 0.1 and beta of 0.1, a total number of 10 patients was considered sufficient.

Common practice in single-arm phase II trial is to set both alpha and beta equal to 0.1.

Statistical analysis
Line 118, statistical analysis section and the statistical test(s) to be stated.

A Wilcoxon signed-rank test was used to compare the data from the questionnaires, with a risk alpha of 0.05. This sentence was added in the Material and Method section (line 133-134).

Line 119, the sentence ‘Proportions were reported as means (sd) or medians [IQR], when appropriate.’ requires revision.

Continuous data were reported as means (sd) or medians [IQR] (if the data is non-normal), while categorical data were summarized as counts and percentages.

We corrected the sentence (line 130-131).

Results
Baseline characteristics of the patients to be provided.

The baseline characteristics are provided in the text, as well as in table 1 and fig 2: “Median [IQR] age was 64 [61-72], median PSA was of 5 [4.3-8.1] ng/mL, and median prostate volume of 50 [40-55] mL. Seven and 3 patients had a low and intermediate D’Amico risk cancer, respectively. Three patients had multifocal disease, with evidence of non MRI-visible secondary tumor foci of Gleason 3+3 cancer on systematic biopsies. The 7 other patients had no evidence of cancer on systematic biopsies. Index tumor characteristics are reported in table 1 and fig 2. Baseline median [IQR] IPSS, IIEF-5, and MSHQ-EjD were of 8.5 [4-13], 18 [13-23], and 10 [7-13], respectively.”

Table 1 provides the baseline histological characteristics of the index tumor, and fig 2 reports the location of the index tumors.

Line 156, the sentence Median [Q1-Q3] largest dimension of necrosis was of 17.5 [16-22] mm’ requires revision.

We corrected and changed [Q1-Q3] to [IQR].

Line 160-164, ensure the data are presented in Table 2.

The data are presented in Table 2 (new table 3 in the revised version). In order to improve readability, we changed “first patient” to “patient number 5” and “second patient” to “patient number 8”

Table 3, the statistical test to be denoted in the table footnote. Decimal point for p value to be standardized. Effect size, 95% CI could be explored/presented. Table alignment could be improved.

P value has been corrected accordingly, with a decimal point. Table alignment has been improved, and the statistic method used has been described.

Line 191-192, the number of patients at various time period to be clearly denoted/stated in the table footnote.

We added the number of patients at the different time periods in Table 3 (new table 4 in the revised version).

Data were collected at various time point but statistical analyses were performed between baseline and 6-month. Nonetheless, due to 'small' sample size, the statistical analysis to be treated with cautious.

We completely agree on this point. The small sample size may not allow a relevant clinical demonstration. However, it may suggest that this treatment may not have a significant impact the sexual and urinary function.

Figure 1, the time period, n to be stated. The number of patients assessed/evaluated for each measure to be illustrated in the figure or to be stated in the method/results section.

We added the time period in the follow-up section of the flow chart.

List of references could be improved e.g. spacing of page number.

Changes have been made accordingly.

**Reviewer #2:**

1. For the three patients with multifocal disease, what is the maximal core length on non-targeted biopsies and what is the number of systematic biopsies involved out of 10-12 cores ? Did these patients have inferior PSA response at 6 months?

These 3 patients were patients number 4, 5 and 7:

Patient 4 had 2/10 systematic cores involved with 4 and 5 mm of cancer Gleason 3+3. Baseline PSA was 8.2 ng/mL. It was 7.8 at 2 months follow-up, and 9.4 at 6 months follow-up. PSA response was not favorable. Six months biopsies did show Gleason 3+4 disease on systematic cores and around the treated area.

Patient 5 had a single systematic core involved with 5 mm of Gleason 3+3 cancer. This patient experienced an immediate failure of focal microwaves and was treated with radical prostatectomy.

Patient 7 had 2/10 systematic cores involved with 3 and 1 mm of cancer Gleason 3+3. His PSA response was not favorable (baseline PSA of 4.4, increased at 5.4 at 2 months, and 7.1 at 6 months). Six months biopsies did not show evidence of cancer on targeted cores (treated area), but Gleason 3+4 disease on systematic cores.

Clearly, these 3 patients were not good candidates for active surveillance or any kind of focal therapy. This raises again the issue of staging and characterization. This point is discussed in the manuscript (discussion section, lines 283-290)

2. What is the rationale of using 1 or 2 treatments? Is it for coverage of a larger tumor or larger margin?

The rationale of performing a second ablation is indeed to increase tumor coverage. In the two patients in whom we performed 2 contiguous ablations (patients 5 and 8), however, we failed to cover entirely the index tumor, as shown on MRI 7 days after ablation. These patients had large tumors (15 and 20 mm of the longest axis), and the software we were using at that time did not allow to visualize the ablated area. It was therefore difficult to find precisely the optimal location where to apply the second ablation.

3. The authors described the treated area is based on pre-clinical predictive ablation chart. How did the post-treatment largest dimension of necrosis area on T1-contrast sequence compare with the predictive ablation chart in each of the 10 patients? Please show the largest dimension of the necrosis and also the expected dimension.

We used 3 different settings: 15W during 3 min, 15W during 2 min, and 10W during 2 minutes (reported in the manuscript lines 157-159). For each setting, we were anticipating a volume of necrosis, based on the ablation charts we had developed.

We added a table (new table 2) comparing, for each patient, the largest dimension of necrosis measured on MRI with that of our predictive ablation chart (line 171-175).

New table 2:

| **N° Patient** | **Ablation power (in W)** | **Ablation time (in min)** | **Largest dimension of ablation on preclinical charts (in mm)** | **Largest dimension of necrosis on day 7 DCE MRI (in mm)** | **Difference (in mm)** |
| --- | --- | --- | --- | --- | --- |
| 1 | 10 | 2 | 17,00 | 17,74 | 0,74 |
| 2 | 15 | 2 | 20,60 | 18,65 | -1,95 |
| 3 | 15 | 2 | 20,60 | 19,48 | -1,12 |
| 4 | 10 | 2 | 17,00 | 16,50 | -0,50 |
| 6 | 15 | 2 | 20,60 | 19,20 | -1,40 |
| 7 | 15 | 3 | 22,60 | 19,95 | -2,65 |
| 9 | 15 | 2 | 20,60 | 21,10 | 0,50 |
| 10 | 15 | 3 | 22,60 | 21,04 | -1,56 |

Correlation could not be performed in patients 5 and 8, because these patients underwent multiple ablations.

4. What is the reason that 2 patients had only 25-40% of the necrosis of the index tumor? Was it due to treatment power (inadequate treatment) or accuracy of targeting (necrosis size adequate BUT not overlapping well with tumor area)?

In these 2 patients, as commented in the answer to question 2, the index tumor was large and we had to perform 2 contiguous ablations. Our hypothesis is that we failed to target precisely the second ablation. Necrosis size was acceptable but not overlapping well with tumor area.

5. For the patient with radical prostatectomy performed showing pT3a Gleason 3+3, what was the size of the residual tumor and also the size of the treated area?

This question is very interesting and relevant. Unfortunately, we could not measure precisely the size of the treated area because we could not see it precisely. We think that this may be due to shrinkage of the ablated tissue during the 3 months period between focal treatment and radical prostatectomy. On the specimen, the size of the index tumor was 21 mm X 10 mm on 5 consecutive sections.

6. Was the radical prostatectomy difficult after microwave treatment?

The surgery was uneventful. We did not experience any difficulty in dissection, nor observed any differences as compared to other patients. We added the word “unremarkable” (line 182).

7. Microwave treatment in liver would lead to shrinkage of treated area. Would the extent of necrosis be under-estimating the actual treated area ?

We indeed think that there is shrinkage of the treated area. Unpublished results suggest that shrinkage may occur shortly after ablation, and lead to underestimation of the treated area.

In our study, we decided to perform MRI seven days after treatment. Shrinkage may already have occurred during the first week after ablation. We did not perform any MRI before 7 days, and did not monitor the necrosis area on consecutive MRIs.

On the MRIs performed at 6 months, we could not observe any necrosis because of complete shrinkage.

8. What is the reason that 4 out of 8 patients with 100% necrosis of index tumor be having residual tumor? Would it be underestimation of tumor by MRI +/- lack of margin coverage?

These are our 2 main hypotheses, as discussed in the manuscript (lines 272-287). In this pilot study, safety was our priority, and we did not apply any margins. We think that these margins are of major importance.

9. In Figure 2, there are 4 tumors that are in peripheral zone. Are they more than 5mm from rectum during treatment? Would close proximity to rectum be the cause of potential undertreatment and local recurrence? Can you add the Patient number to the tumor locations in Figure 2?

During treatment, we were measuring the distance from the rectum using ultrasound. No patient had less than 5 mm distance between the the tumor and the rectum wall. However, patients 1 and 4 had no more than 5 mm distance between index tumor and rectum wall. Patient 1 had an index tumor located in the posterior pz, at 5 mm from the rectum wall. This patient showed residual tumor around the treated area on the 6 months biopsies (reported in table 3 of the revised version).

We modified Figure 2 in order to show patient number and tumor location. Index tumors have been represented with a 1:1 scale on a prostate with 4 cm height (antero-posterior axis), 6 cm width (lateral axis).

10. Can you show the pre and post treatment MRI films of the 4 tumors in peripheral zone?

The 4 tumors in peripheral zone correspond to patients 1, 2, 4 and 6. Figure 3 shows the pre and postoperative MRI image of patient 2. As suggested by the reviewer, we added in Figure 3 the pre and post treatment MRI images of the 3 other patients with an index tumor located in the pz.

11. There are 3 cases with pre-op systematic biopsies showing no Gleason 3+4 cancer but post-op 6 month biopsy showing Gleason 3+4. So, the pre-op MRI missed 3 out of 10 clinically significant cancer, which was much higher than the reported figure (around 90% sensitivity). Would this be due to quality of MRI or just a matter of inadequate systematic sampling (10-12 cores) via transrectal biopsy?

We think it may be due to both, although we are confident with the MRIs performed by the co-investigators. These significant tumors were harboring only 10 to 15% of grade 4 cancer, which may also explain why they were not visible.

12. Line 251: Can you add margin suggestion in consensus statement on focal therapy?

A consensus meeting published in European Urology in 2015 suggested a margin of 5 mm. We added a sentence and the reference (new reference 22): Donaldson IA, Alonzi R, Barratt D, Barret E, Berge V, Bott S, et al. Focal therapy: patients, interventions, and outcomes--a report from a consensus meeting. Eur Urol. 2015; 67: 771-7. (line 275-277)

13. What intra-prostatic margin would you suggest for microwave ablation for prostate tumors?

Because there are no evidence-based recommendation, it is difficult to have a global suggestion on the matter. As discussed in the manuscript (line 272-283), we would suggest a safety intra-prostatic margin of at least 3 mm, on a case-by-case basis, depending also on the location and the volume of the index tumor. More studies are needed to evaluate the length of the margin to be applied.

14. Minor amendments:

a. Please change IPSS-QDV to QOL in English.

We apologize for this mistake. It has been corrected.

b. Line 239: recal -> recall

It has also been corrected.

c. Please update reference 2.

This reference has been corrected accordingly (new reference number 4).

**Reviewer #3:**

This manuscript reported that the “first” experiment of OBT-targeted FMA for the treatment of patient with low to intermediate risk prostate cancer. The main concern was safety of this technique in the patients, which is very significative for the focal therapy in the low to intermediate risk prostate cancer patients.
However, there are some points for improvement in this manuscript.
There are many grammatical and linguistic errors throughout the manuscript. The authors should let the entire manuscript be reviewed by a professional English-speaking writer.

Thank you for this suggestion. An English-speaking writer has reviewed the entire manuscript.

Title

Please explain the word “FOSTINE”.

FOSTINE stands for “FOcal Secured Targeted INduced Energy”

Abstract

Line 33, the word “at day 7” should be changed into “one week after ablation” or “7 days after ablation”, which is more precise.

We corrected this wording all along the manuscript

Line 33, Please adjust the sentence “Secondary endpoints were adverse events, urinary and sexual functional outcomes, assessed with IPSS, IIEF5 and MSHQ-EjD-SF, and oncological outcomes, assessed with PSA at 2 and 6 months, and re-biopsy at 6 month” to make more clear in the presentation.

The sentence was corrected and changed to a more understandable phrasing (line 33-35).

Introduction

Line 56, Please explain the word “Index tumor”, and give the theory and significances about ablation on the index lesion of prostate cancer.

Thank you for this suggestion. We added 3 sentences of explanation (line 51-56), as well as two references:

Noguchi M, Stamey TA, McNeal JE, Nolley R. Prognostic factors for multifocal prostate cancer in radical prostatectomy specimens: lack of significance of secondary cancers. J Urol. 2003; 170: 459-63.

Wise AM, Stamey TA, McNeal JE, Clayton JL. Morphologic and clinical significance of multifocal prostate cancers in radical prostatectomy specimens. Urology. 2002; 60: 264-9.

Line 58 Through you gave the reference about the “organ-based tracking (OBT) MRI-ultrasound fusion”, may be it is more appropriate to provide a brief explanation and explanation about the OBT MRI-ultrasound fusion.

Thank you for this suggestion. We added explanations (line 63-66).

Patients and Method

Line 79 Why the age in the inclusion criteria is between 45 and 76 years?

We decided not to include patients older than 76 years old. The potential benefit of an active treatment is not demonstrated in old patients. Also, these patients are more fragile, and may have been more at risk for the evaluation of this new technology. The benefit/risk ratio was therefore not favorable.

Line 78-85 Whether the coagulation function, infection status and cardio-pulmonary function in the inclusion and exclusion criteria.

The presence of untreated bacteriuria 48h before surgery was an exclusion criteria.

Coagulation function and cardio-pulmonary status were not part of exclusion criteria.

Contraindication to general anesthesia was an exclusion criteria.

We added these exclusion criteria in the manuscript (line 92-93).

Line 92-93 The expression that “(2) The urinary and sexual outcomes, assessed with IPSS, IIEF5 and MSHQ-EjD-SF, self-questionnaires, respectively” will be more precise.

We corrected this sentence accordingly (line 100).

Line 97-103 Please describe the preoperative preparation, such as bowel preparation and so on.

All patients received prophylactic preoperative antibiotics using oral fluoroquinolones, and a bowel preparation. This was added in the manuscript (line 105-106)

Please add images of fusion and puncture or ablation process.

We added images of fusion and ablation process of patient number 3 (new figure 4, legends added line 320-326).

Line 108-109 How to calculate the proportion? By volume or by length?

We corrected the sentence accordingly.

Line 113-117 How to calculate the sample? What formula or software is used?

R software was used to calculate the sample size with the ph2single() function (clinfun package).

Line 119 Please detailed description that the data for application of means (sd) or medians [IQR].

We corrected accordingly in the manuscript (line 130-131).

Results

Line 127 “The median age was 64 [61-72] years”.
The number should be described consistently, such as “seven and 3” or “7” and so on.

We corrected accordingly (line 140-141).

Line 135 and Line 168 Please change the table caption into a complete sentence.

We corrected accordingly

Line 136 Please draw the diagram properly. This table is very terrible, which is confusion.

We apologize for this. Corrections were made accordingly.

Line 145-147 How to judge the ablation necrosis cover the tumor mass during the ablation?

During the procedure, we could not visualize the tumor or the ablation process. We could not therefore evaluate the coverage of the tumor. We could only rely on OBT MRI- ultrasound fusion (showing the location of the tumor on the station in a real-time fashion), and on the preclinical ablation charts (indicating a size of ablation for each setting of the microwave generator).

Line 149 Please try to explain the cause of spontaneous micturition in the discussion. Does any patient had hematuresis symptoms?

None of the patients had intra-operative urethral catheterization, and all of them voided spontaneously a few hours after surgery. This result suggests that the ablation did not induce any important edema. None of the patients reported hematuria after the procedure. A sentence was added in the Discussion section (lines 238-241).

Line 143-151 How to protect the urethra during ablation? Whether the indwelling catheter was needed during the ablation process? Whether to take antibiotics after ablation?

None of the patients required an indwelling catheter (line 107). None of the patients had any antibiotics other than a preoperative prophylaxis with oral fluoroquinolone (line 107).

We think that the prostatic urethra does not need any protection during ablation. The microwave effect seems to have sharp limits. None of the 10 patients had a tumor close to the urethra. However, our hypothesis is that tumors close to the urethra may also be treated without increased morbidity. This last hypothesis is out of the range of this pilot study, and needs confirmation with further trials.

Line 169 Please draw the table properly.

We apologize. Changes have been made accordingly.

Discussion

Line 219 The first sentence will be more appropriate if changed into “The results of this study was satisfactory”.

Changes have been made accordingly (line 238).

Line 219-220 What does mean for the sentence that “First, no patient experienced adverse events nor was altered sexual or urinary function reported during the 6-month follow-up, confirming the safety of this technique.”

Safety was a very important concern. This pilot study suggested the good tolerance profile of the technique. We changed the phrasing to make this sentence more understandable (lines 238-241).

Line 234-235 What does mean for the “but oriented from the right to the left.”

We changed the sentence to: “The second patient had a smaller tumor, but with its largest axis oriented transversely” (line 255).

Line 246 In term of oncological outcome, dose any changes was detected from image other than PAS changes.

We did not detect any suspicious changes on MRI images at 6 months.

Line 287-289 Why do the index tumor lesions vary in size from the figure? Should they be scaled according to actual size?

The figure 2 has been corrected and reshaped in order to be more comprehensive, and the size has been scaled according to tumor largest axis.
