# Supplementary material for: Feasibility and safety of targeted focal microwave ablation of the index tumor in patients with low to intermediate risk prostate cancer: Results of the FOSTINE trial
Source: PLoS One. 2021 Jul 14;16(7):e0252040. doi: 10.1371/journal.pone.0252040 (PMC8279354; doi:10.1371/journal.pone.0252040)
Supplement: Attachment — Submitted filename: ANSWER TO REVIEWERS_second revision.docx [file pone.0252040.s007.docx]

ANSWER TO REVIEWERS

The authors would like to thank again the reviewers for their comments and suggestions. All points were addressed, and changes were made accordingly in the manuscript.

Reviewer #1: Minor comment(s)

Line 127 - two-tailed test to be placed in the statement.

We specified that it was a two-tailed test (line 128).

Table 4 - at least 2 decimal points for p value to be presented. The reason for different 'n' in the follow up to be described in the results.

P values were corrected accordingly. We also added a statement explaining the reason for the different numbers of patients included in the analysis (lines 204-207).

For the demographic characteristics, apart from age, were there any other information could be displayed to describe the patients i.e ethnicity etc.

Unfortunately, we did not include any other demographic characteristic in our CRF.

In the abstract, it was mentioned that IPSS, IIEF-5 and MSHQ-EjD-SF were not statistically different at baseline, 7 days, 2 and 6 months but there was no statistical test performed to look into the difference between the time points except baseline and 6 months. This requires revision.

We apologize for this. The abstract was revised accordingly (line 44).

Reviewer #2: Thank you for addressing all the comments. The figures clearly showed the tumor locations and the table showed the ablation dimensions well.

Reviewer #3: Dear Author:

Thank you for revising your paper. The paper was better than last time in the understanding the meaning of the text and the format. However, there are some small nibs for improvement in this manuscript.

1. Where is reference 1 in the text?

Thank you for raising this point. We corrected (line 54).

2. The table in the paper should be in third line in format, which including the table top line, column line, and bottom line, but without the vertical line. Obviously, the table 1 and table 2 did not conform to this format. Besides, there were two headers in table 1, which is confusion.

We corrected the format of the tables

3. Which sequences are included in multi-parameter MRI? Please give a detailed explanation in this paper.

Multi-parametric pMRI includes T2 and diffusion weighted imaging, as well as dynamic contrast-enhanced imaging (interesting especially for the evaluation of necrosis 7 days after ablation). We added a sentence in the manuscript (lines 115 and 116).

4. The author did not answer the question that how to calculate the proportion of necrosis to the index tumor in the mpMRI 7 days after ablation? By volume or by length? Please describe this in the method.

In the absence of complete necrosis covering of the index tumor, the proportion of necrosis extent was visually estimated, according to the surface leaved untreated on each MRI image. Two radiologists estimated the images, blinded to the patients characteristics. We added the description in the method section (line 120-121).

5. Did these ten patients in this pilot clinical trial take antibiotics after ablation? Please add the information to the text.

None of the patients received any antibiotics after surgery. We added the information in the text (line 165 in the result section).

6. It is pleasure for the results that there was no urethral injury in all the patients. Could you provide the distance between the lesion and the urethra? I think this can offer certain guidance to the clinical application in the future.

Unfortunately, the length between the index tumors and urethra was not recorded in our CRF. The prostatic urethra is included in the parenchyma of the gland, and ablation does not impact long-term functional outcomes. The immediate risks are related to hematuria, but usually mild, and spontaneously resolute after a few days. In our study, as mentioned in the result section, no patient reported any pain or hematuria after ablation.

7. In regard to the question that whether to take antibiotics after ablation?

We do not consider that there is a need to take antibiotic after microwave focal ablation. The different protocols of focal ablation using cryotherapy, HIFU, interstitial laser or electroporation do not recommend the use of postoperative antibiotics.Reviewer #1: Minor comment(s)

Line 127 - two-tailed test to be placed in the statement.

Table 4 - at least 2 decimal points for p value to be presented. The reason for different 'n' in the follow up to be described in the results.

For the demographic characteristics, apart from age, were there any other information could be displayed to describe the patients i.e ethnicity etc.

In the abstract, it was mentioned that IPSS, IIEF-5 and MSHQ-EjD-SF were not statistically different at baseline, 7 days, 2 and 6 months but there was no statistical test performed to look into the difference between the time points except baseline and 6 months. This requires revision.

8. Reviewer #2: Thank you for addressing all the comments. The figures clearly showed the tumor locations and the table showed the ablation dimensions well.
9. Reviewer #3: Dear Author:
   Thank you for revising your paper. The paper was better than last time in the understanding the meaning of the text and the format. However, there are some small nibs for improvement in this manuscript.
   1. Where is reference 1 in the text?
   2. The table in the paper should be in third line in format, which including the table top line, column line, and bottom line, but without the vertical line. Obviously, the table 1 and table 2 did not conform to this format. Besides, there were two headers in table 1, which is confusion.
   3. Which sequences are included in multi-parameter MRI? Please give a detailed explanation in this paper.
   4. The author did not answer the question that how to calculate the proportion of necrosis to the index tumor in the mpMRI 7 days after ablation? By volume or by length? Please describe this in the method.
   5. Did these ten patients in this pilot clinical trial take antibiotics after ablation? Please add the information to the text.
   6. It is pleasure for the results that there was no urethral injury in all the patients. Could you provide the distance between the lesion and the urethra? I think this can offer certain guidance to the clinical application in the future.
   7. In regard to the question that whether to take antibiotics after ablation?
10. Reviewer #1: Minor comment(s)

    Line 127 - two-tailed test to be placed in the statement.

    Table 4 - at least 2 decimal points for p value to be presented. The reason for different 'n' in the follow up to be described in the results.

    For the demographic characteristics, apart from age, were there any other information could be displayed to describe the patients i.e ethnicity etc.

    In the abstract, it was mentioned that IPSS, IIEF-5 and MSHQ-EjD-SF were not statistically different at baseline, 7 days, 2 and 6 months but there was no statistical test performed to look into the difference between the time points except baseline and 6 months. This requires revision.
11. Reviewer #2: Thank you for addressing all the comments. The figures clearly showed the tumor locations and the table showed the ablation dimensions well.
12. Reviewer #3: Dear Author:
    Thank you for revising your paper. The paper was better than last time in the understanding the meaning of the text and the format. However, there are some small nibs for improvement in this manuscript.
    1. Where is reference 1 in the text?
    2. The table in the paper should be in third line in format, which including the table top line, column line, and bottom line, but without the vertical line. Obviously, the table 1 and table 2 did not conform to this format. Besides, there were two headers in table 1, which is confusion.
    3. Which sequences are included in multi-parameter MRI? Please give a detailed explanation in this paper.
    4. The author did not answer the question that how to calculate the proportion of necrosis to the index tumor in the mpMRI 7 days after ablation? By volume or by length? Please describe this in the method.
    5. Did these ten patients in this pilot clinical trial take antibiotics after ablation? Please add the information to the text.
    6. It is pleasure for the results that there was no urethral injury in all the patients. Could you provide the distance between the lesion and the urethra? I think this can offer certain guidance to the clinical application in the future.
    7. In regard to the question that whether to take antibiotics after ablation?
